# Supplementary material for: Dupilumab Alters Both the Bacterial and Fungal Skin Microbiomes of Patients with Atopic Dermatitis
Source: Microorganisms. 2024 Jan 22;12(1):224. doi: 10.3390/microorganisms12010224 (PMC10820602; doi:10.3390/microorganisms12010224)

Figure S1. Fungal microbiome of the lesional site of 30 patients with atopic dermatitis after administration of dupilumab. Information on 10 healthy individuals is also included. Genera with a relative abundance >2% are shown.

Patients with atopic dermatitis

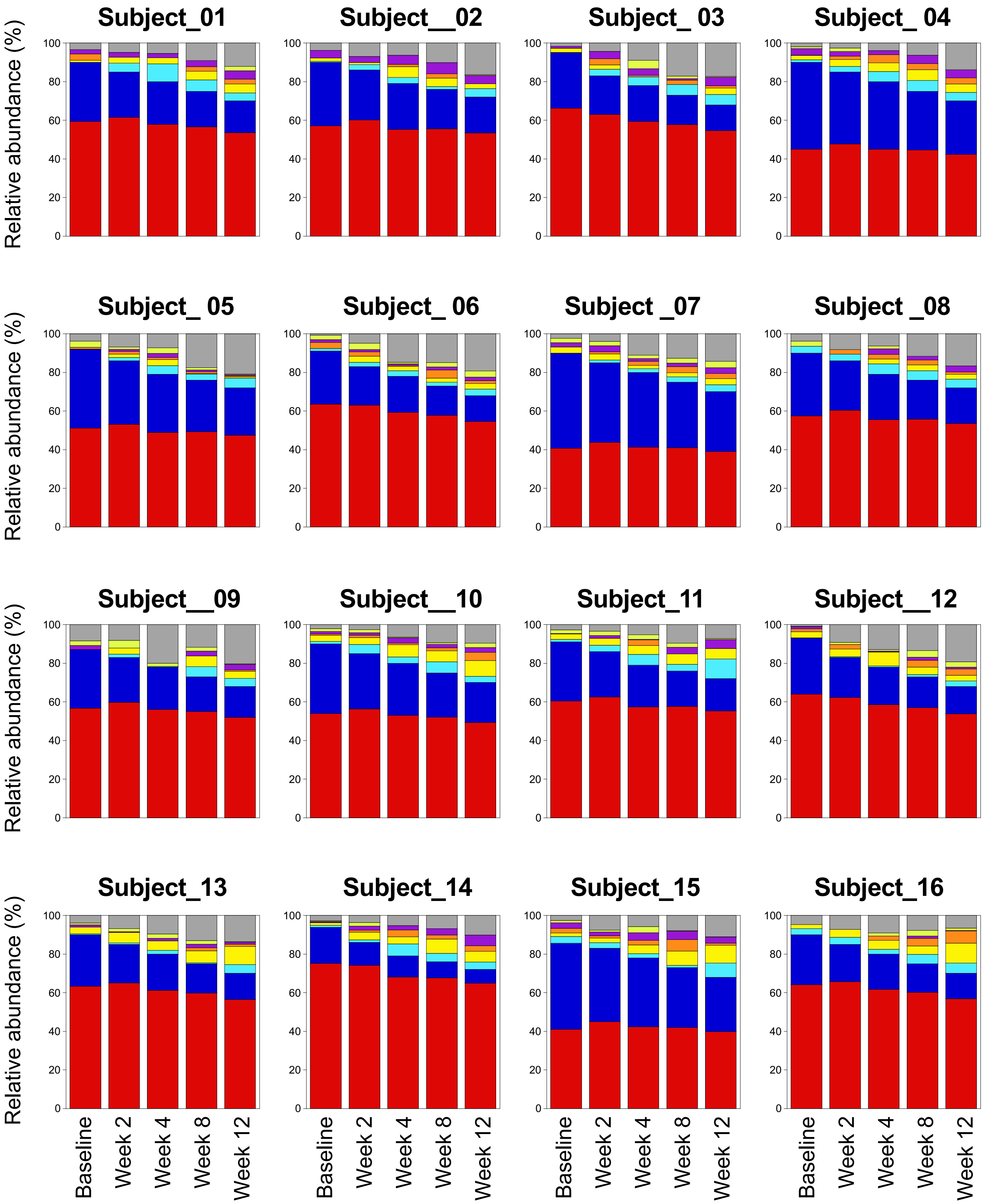

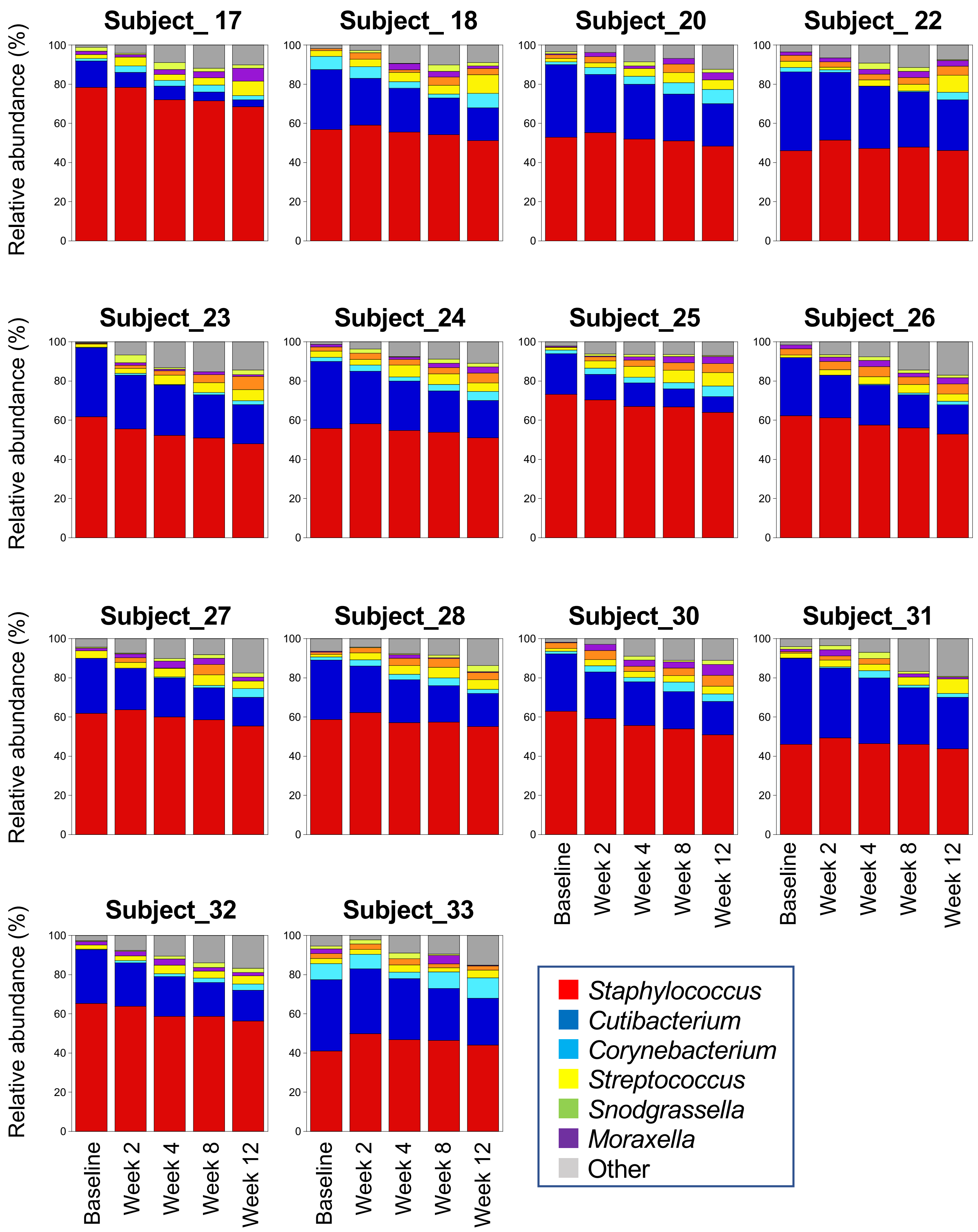
Subject_ 17
Subject_ 18
Subject_20
Subject_ 22
Subject_23
Subject_24
Subject_25
Subject_26
Subject_27
Subject_28
Subject_30
Subject_31
Subject_32
Subject_33
Relative abundance (%)
0
20
40
60
80
100
Baseline
Week 2
Week 4
Week 8
Week 12
Staphylococcus
Cutibacterium
Corynebacterium
Streptococcus
Snodgrassella
Moraxella
Other

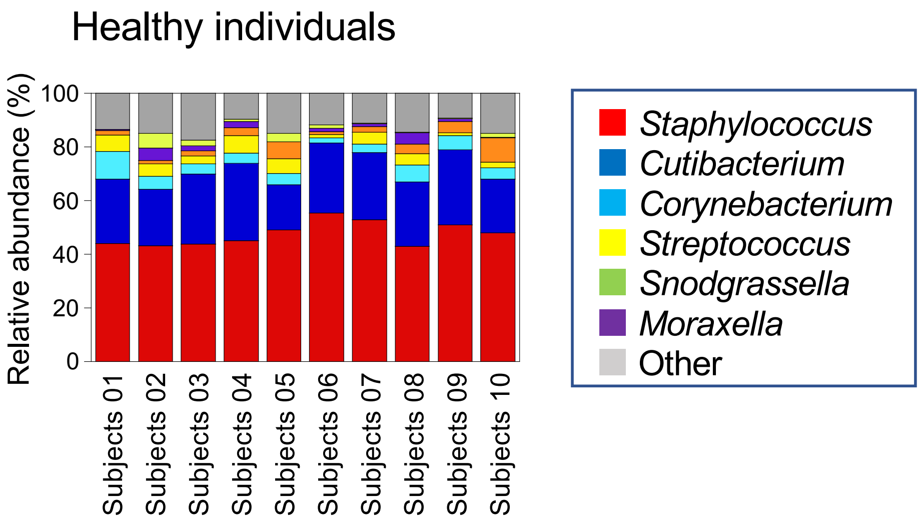
Healthy individuals
Relative abundance (%)
100
80
60
40
20
0
Subjects 01
Subjects 02
Subjects 03
Subjects 04
Subjects 05
Subjects 06
Subjects 07
Subjects 08
Subjects 09
Subjects 10
Staphylococcus
Cutibacterium
Corynebacterium
Streptococcus
Snodgrassella
Moraxella
Other

Figure S2. Bacterial microbiome of the lesional site of 30 patients with atopic dermatitis after administration of dupilumab. Information on 10 healthy individuals is also included. Genera with a relative abundance >3% are shown.

Patients with atopic dermatitis

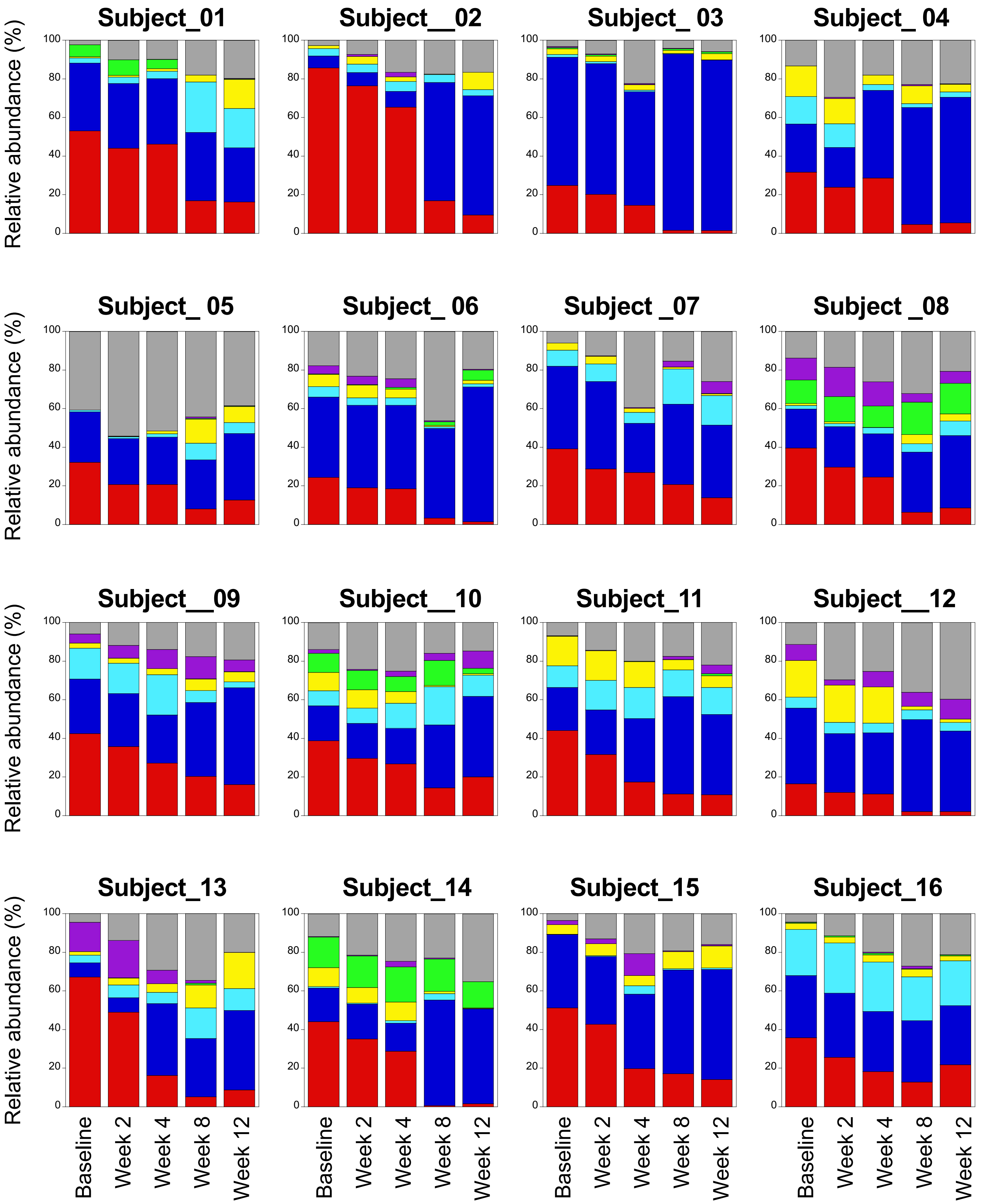

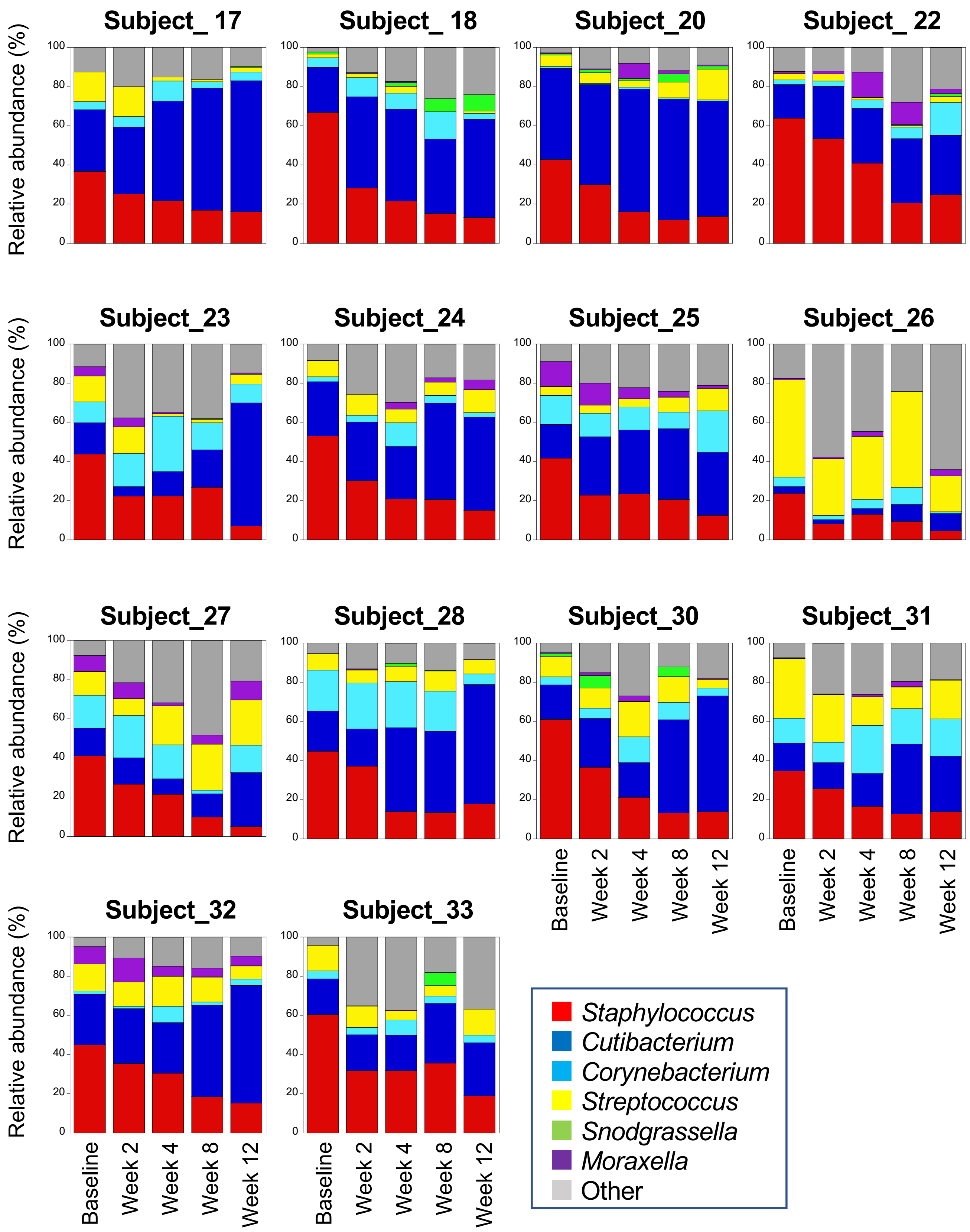
Subject_ 17
Subject_ 18
Subject_20
Subject_ 22
Subject_23
Subject_24
Subject_25
Subject_26
Subject_27
Subject_28
Subject_30
Subject_31
Subject_32
Subject_33
Relative abundance (%)
Baseline
Week 2
Week 4
Week 8
Week 12
Staphylococcus
Cutibacterium
Corynebacterium
Streptococcus
Snodgrassella
Moraxella
Other

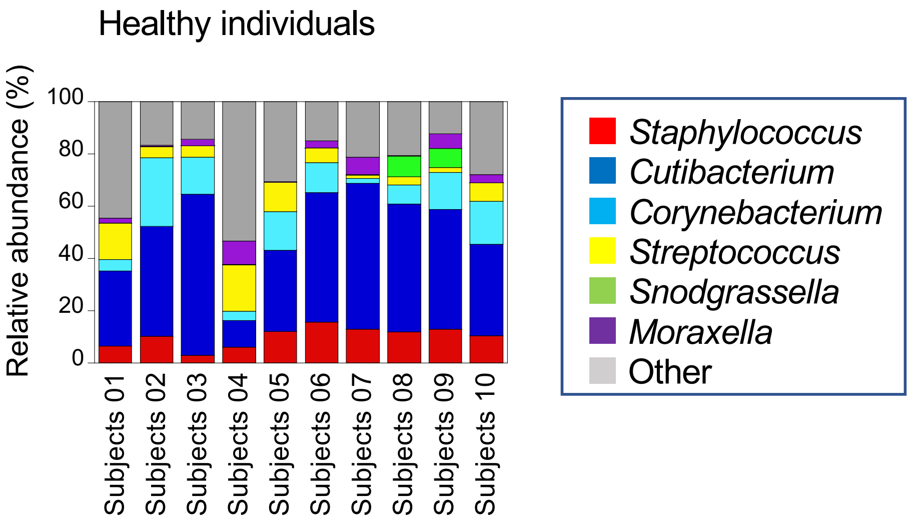

Healthy individuals
Relative abundance (%)
100
80
60
40
20
0
Subjects 01
Subjects 02
Subjects 03
Subjects 04
Subjects 05
Subjects 06
Subjects 07
Subjects 08
Subjects 09
Subjects 10
Staphylococcus
Cutibacterium
Corynebacterium
Streptococcus
Snodgrassella
Moraxella
Other

Figure S3. Correlation of relative abundance between genera *Staphylococcus* and *Cutibacterium*. A good correlation with *r* = 0.5 was observed. Data were calculated from all sampling points (n = 150).

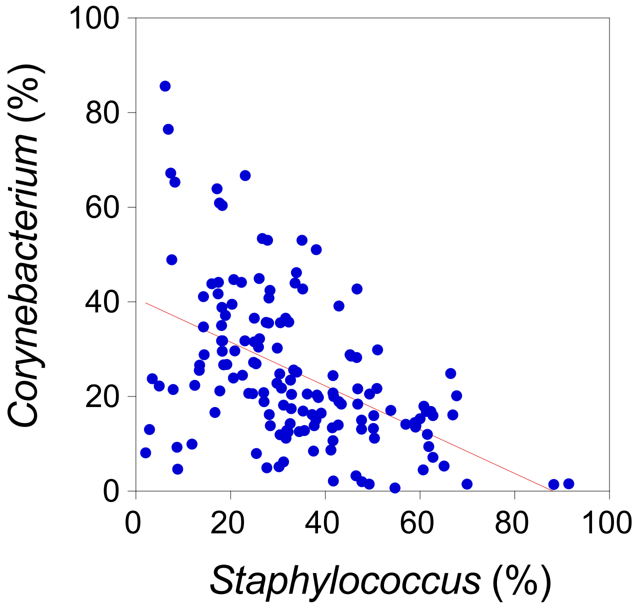

Figure S4. Comparison of colonization levels by *Malassezia* or *Staphylococcus aureus* between patients who achieved EASI-75, and those who did not. NS, not significant; Mann–Whitney U test. EASI, Eczema Area and Severity Index.

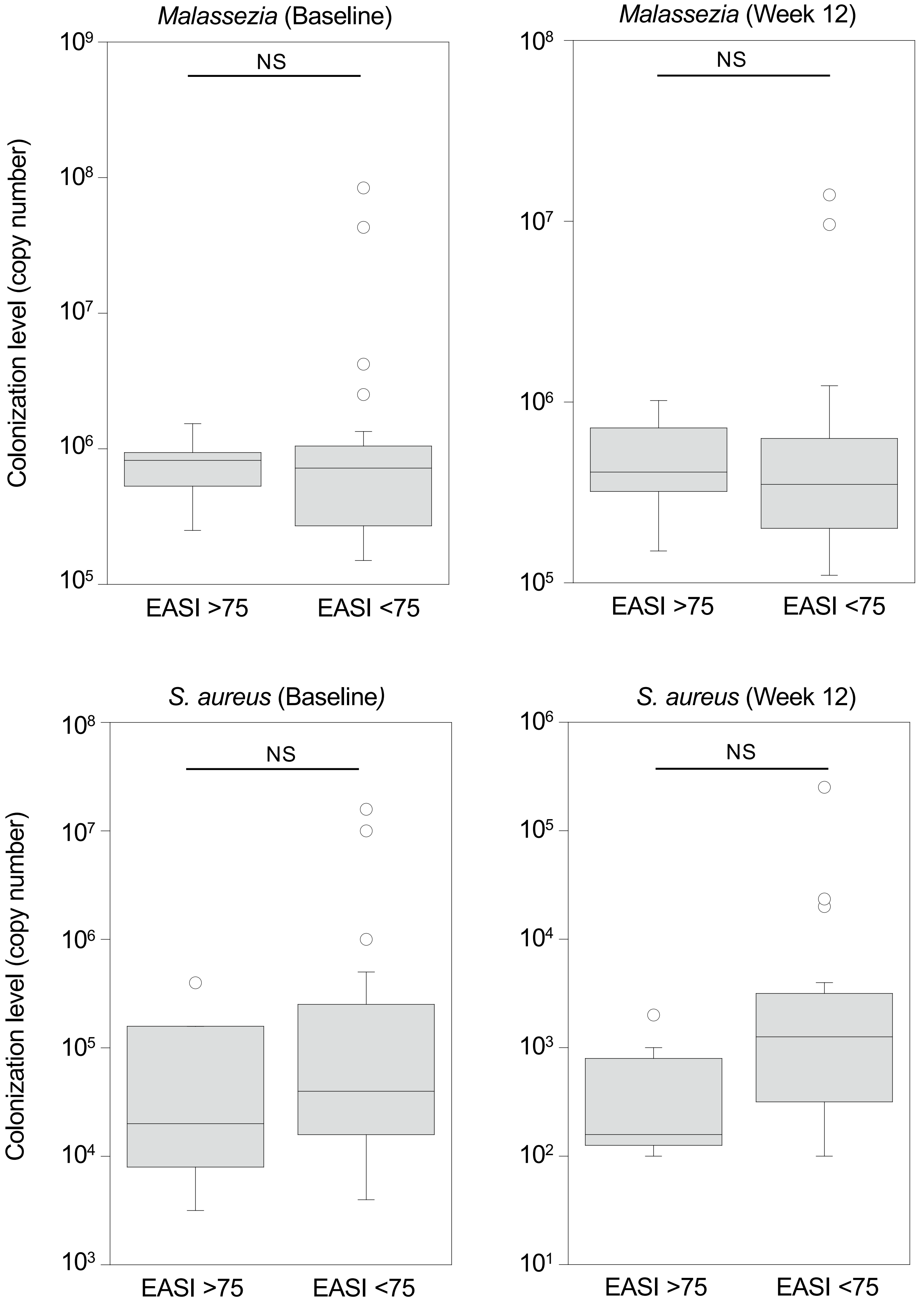

Supplement: Supplementary file 1 [file microorganisms-12-00224-s001.zip › Figure_S.pdf]
