# Supplementary material for: Dupilumab Alters Both the Bacterial and Fungal Skin Microbiomes of Patients with Atopic Dermatitis
Source: Microorganisms. 2024 Jan 22;12(1):224. doi: 10.3390/microorganisms12010224 (PMC10820602; doi:10.3390/microorganisms12010224)
Supplement: Supplementary file 1 [file microorganisms-12-00224-s001.zip › Table_S1_word.pdf]

Table S1 Relative abundance (%) of fungal microbiome of subject involved in this study

	Subject_01					Subject_02					Subject_03					Subject_04					Subject_05				
Taxa	Baseline	Week 2	Week 4	Week 8	Week 12	Baseline	Week 2	Week 4	Week 8	Week 12	Baseline	Week 2	Week 4	Week 8	Week 12	Baseline	Week 2	Week 4	Week 8	Week 12	Baseline	Week 2	Week 4	Week 8	Week 12
Malassezia restricta	59.4	61.5	57.9	56.6	53.6	57.1	60.1	55.2	55.6	53.4	66.3	63.1	59.3	57.8	54.5	45.0	47.8	45.0	44.5	42.3	51.2	53.2	48.9	49.4	47.5
Malassezia globosa	30.6	23.5	22.1	18.4	16.4	32.9	25.9	23.8	20.4	18.6	28.7	19.9	18.7	15.2	13.5	45.0	37.2	35.0	30.5	27.7	40.8	32.8	30.1	26.6	24.5
Malassezia sympodialis	0.0	4.6	9.2	5.9	4.2	0.5	2.8	3.2	1.5	4.4	0.1	3.5	4.5	5.5	5.4	1.3	2.9	5.2	5.6	4.4	0.2	1.8	4.5	3.2	5.1
Malassezia yamatoensis	0.0	0.0	0.1	0.0	1.3	0.0	0.0	0.0	0.0	0.0	0.0	0.0	0.0	0.0	1.4	0.0	0.0	0.0	0.3	1.5	0.0	0.4	1.1	0.4	0.5
Malassezia dermatis	0.0	0.4	0.0	2.3	4.3	0.0	0.0	0.0	0.0	0.0	0.0	0.0	2.1	2.1	1.4	0.0	0.5	0.0	0.0	0.2	0.0	0.0	0.0	0.0	0.0
Malassezia japonica	0.0	0.0	0.0	0.1	0.0	0.0	0.0	0.0	0.0	0.0	0.0	0.0	0.0	1.2	0.3	0.0	0.0	0.0	0.0	0.1	0.0	0.0	0.0	0.0	1.5
Malassezia obtusa	0.0	0.0	0.2	0.0	0.0	0.0	0.0	0.4	0.0	0.0	0.0	0.5	0.0	0.0	0.1	0.0	0.0	0.0	1.1	1.1	0.0	0.0	0.5	2.0	0.0
Malassezia furfur	0.0	0.0	0.0	0.0	0.0	0.0	0.0	0.0	0.0	0.0	0.0	0.0	0.3	0.0	0.3	0.0	0.0	0.0	0.0	0.0	0.0	0.0	0.0	0.0	0.2
Malassezia slooffiae	0.2	0.0	0.0	0.0	0.2	0.0	1.0	0.0	0.0	0.0	0.0	0.0	0.2	1.2	0.0	0.2	0.0	0.0	0.4	1.1	0.0	0.0	0.0	0.4	0.6
Aureobasidium	0.0	0.0	0.0	0.4	1.2	0.0	0.0	2.3	0.0	1.1	0.0	0.0	0.0	2.9	1.4	0.0	0.0	0.0	0.0	0.0	2.2	0.0	2.4	0.5	0.5
Rhodotorula mucilaginosa	2.3	2.5	2.2	3.2	4.3	4.0	3.2	4.8	5.7	4.3	1.1	3.7	3.5	0.8	4.4	3.4	2.6	2.1	4.3	4.3	0.0	1.1	2.2	1.0	0.6
Aspergillus	0.0	0.0	0.0	0.0	2.3	0.0	0.0	0.0	0.0	0.5	0.0	0.0	4.3	1.3	0.4	1.0	1.7	0.0	0.0	0.0	3.2	1.1	2.9	1.1	0.4
Cladosporium	3.2	0.0	0.0	2.3	2.5	0.0	0.0	1.2	2.3	0.0	0.0	3.2	0.7	2.2	1.3	0.2	1.7	4.3	3.3	3.2	0.9	1.4	1.0	0.9	0.5
Torula herbarum	0.0	0.0	0.0	0.0	0.0	0.0	0.0	0.2	0.5	0.0	0.0	0.0	0.0	0.0	0.0	0.0	0.0	0.0	0.3	0.2	0.0	0.0	0.0	0.0	0.2
Naganishia friedmannii	0.0	0.0	0.0	0.0	0.2	0.0	0.0	0.0	0.0	0.0	0.1	1.5	0.0	1.2	0.3	0.0	0.0	0.0	0.0	0.2	0.0	0.6	0.5	0.3	0.2
Candida albicans	0.5	0.3	0.2	0.0	0.0	3.2	3.0	2.2	0.3	0.6	1.5	0.6	0.4	0.4	0.0	1.4	0.5	0.1	0.1	0.1	1.6	0.5	0.2	0.4	0.1
Candida parapsilosis	1.1	3.0	3.2	4.5	4.6	1.7	1.0	5.5	4.3	2.5	2.2	2.2	0.0	0.0	3.2	2.2	3.5	4.5	5.5	4.3	0.0	1.7	3.2	0.2	0.6
Alternaria	2.2	1.1	0.2	0.0	1.0	0.0	0.0	0.0	2.2	1.3	0.0	0.0	0.0	1.1	0.0	0.0	0.0	0.0	0.0	0.0	0.0	0.0	0.0	0.4	0.0
Cystobasidium slooffiae	0.0	0.3	0.0	0.0	0.0	0.2	1.0	0.0	0.0	0.0	0.0	0.0	0.0	0.0	0.2	0.0	0.0	0.3	0.3	1.4	0.0	1.5	0.0	0.0	0.0
Pichia membranifaciens	0.0	0.0	0.3	0.0	0.0	0.0	0.0	0.8	0.0	0.0	0.0	0.0	0.0	0.0	0.0	0.0	0.0	0.0	0.0	0.3	0.0	0.0	0.1	0.2	0.2
Sympodiomycopsis	0.0	0.2	0.0	0.1	0.0	0.0	0.0	0.0	0.0	0.0	0.0	0.0	0.0	0.0	0.0	0.0	0.6	0.3	0.0	0.0	0.0	0.0	0.2	0.1	0.4
[Candida] glaebosa	0.0	0.0	0.3	0.0	0.1	0.0	1.0	0.0	1.1	1.5	0.0	0.0	0.0	1.4	0.1	0.0	0.0	0.0	0.0	0.0	0.0	1.4	0.2	0.3	0.5
Filobasidium floriforme	0.1	0.0	0.0	0.0	0.1	0.0	0.0	0.0	0.0	0.0	0.0	0.0	0.0	0.0	0.0	0.0	0.0	0.0	0.1	0.0	0.0	0.0	0.0	0.0	0.2
Erythrobasidium hasegawianum	0.0	0.0	0.0	0.0	0.0	0.0	1.0	0.0	0.0	0.0	0.0	0.2	0.0	0.1	0.0	0.0	0.0	0.1	0.0	0.0	0.0	0.0	0.0	0.0	0.0
Knufia	0.0	0.0	0.1	0.0	0.1	0.0	0.0	0.0	0.0	0.0	0.0	0.0	0.0	0.0	0.0	0.0	0.0	0.0	0.0	0.1	0.0	0.0	0.0	0.0	0.0
Vishniacozyma carnescens	0.0	0.3	0.0	0.0	0.1	0.0	0.0	0.4	0.2	1.1	0.0	0.0	0.4	0.0	0.0	0.0	0.0	0.0	0.3	0.0	0.0	0.0	0.2	0.0	0.0
Cystobasidium minutum	0.0	0.0	0.0	0.1	0.1	0.0	0.0	0.0	0.0	0.0	0.0	0.0	0.0	0.0	0.3	0.0	0.0	0.1	0.0	0.0	0.0	0.0	0.2	0.0	0.0
[Candida] zeylanoides	0.0	0.0	0.3	0.0	0.3	0.0	0.0	0.0	0.0	0.0	0.0	0.0	0.0	0.0	0.0	0.2	0.5	0.3	0.0	0.1	0.0	0.6	0.0	0.0	0.0
Fusarium	0.0	0.0	0.0	0.0	0.1	0.0	0.0	0.0	0.0	1.1	0.0	1.6	0.0	0.0	0.0	0.0	0.0	0.2	0.4	0.0	0.0	0.0	0.0	1.4	0.0
Debaryomyces hansenii/fabryi/vindc	0.0	0.0	2.3	0.0	0.1	0.0	0.0	0.0	0.0	0.0	0.0	0.0	2.3	0.0	0.5	0.0	0.0	0.2	0.0	0.0	0.0	0.0	0.2	3.5	1.2
Villosiclava	0.0	0.0	0.0	0.0	0.4	0.0	0.0	0.0	1.1	0.0	0.0	0.0	0.0	0.0	0.0	0.0	0.0	0.0	0.3	0.0	0.0	0.0	0.0	0.2	0.0
Trichosporon coremiiforme	0.0	0.1	0.0	0.0	0.1	0.0	0.0	0.0	0.0	0.0	0.0	0.0	0.0	0.0	0.2	0.0	0.0	0.0	0.2	0.0	0.0	0.0	0.0	0.0	0.0
Saitozyma flava	0.0	0.0	0.0	0.0	0.0	0.0	0.0	0.0	0.0	1.2	0.0	0.0	0.5	0.2	0.0	0.0	0.0	0.2	0.1	0.1	0.0	0.5	0.0	0.0	0.0
Cystofilobasidium capitatum	0.0	0.0	0.0	0.0	0.1	0.0	0.0	0.0	0.0	0.0	0.0	0.0	0.0	0.0	0.0	0.0	0.5	0.0	0.2	0.0	0.0	0.0	0.1	1.6	0.0
Sarocladium	0.0	0.0	0.2	0.0	0.1	0.0	0.0	0.0	0.0	0.0	0.0	0.0	0.4	0.0	0.4	0.0	0.0	0.0	0.1	0.2	0.0	0.0	0.3	0.0	0.0
Penicillium	0.0	2.2	0.0	0.0	0.0	0.0	0.0	0.0	0.0	0.0	0.0	0.0	0.0	0.0	0.6	0.0	0.0	0.4	0.2	0.3	0.0	1.4	0.1	0.0	0.0
Verrucaria	0.0	0.0	0.0	0.3	0.1	0.0	0.0	0.0	0.0	0.0	0.0	0.0	0.0	0.0	0.6	0.0	0.0	0.2	0.4	0.4	0.0	0.0	0.0	0.0	0.0
Saccharomyces cerevisiae	0.0	0.0	0.0	0.3	0.1	0.0	0.0	0.0	1.1	0.0	0.0	0.0	0.3	0.0	0.0	0.0	0.0	0.1	0.1	0.2	0.0	0.0	0.0	0.0	0.0
Sterigmatomyces halophilus	0.0	0.0	0.0	0.0	0.0	0.0	0.0	0.0	0.0	0.0	0.0	0.0	0.0	0.3	0.0	0.0	0.0	0.0	0.0	0.0	0.0	0.0	0.0	0.2	0.0
Microporus	0.0	0.0	0.0	0.2	0.1	0.0	0.0	0.0	0.0	0.9	0.0	0.0	0.0	0.0	0.4	0.0	0.0	0.0	0.0	0.0	0.0	0.0	0.4	0.0	0.0
Wallemia	0.0	0.0	0.2	0.0	0.0	0.0	0.0	0.0	1.5	0.0	0.0	0.0	0.7	0.0	0.6	0.0	0.0	0.0	0.0	0.0	0.0	0.0	0.0	0.0	0.0
Papiliotrema flavescens	0.0	0.0	0.0	0.0	0.0	0.0	0.0	0.0	0.0	0.0	0.0	0.0	0.0	0.0	0.2	0.0	0.0	0.0	0.0	0.0	0.0	0.0	0.5	0.0	0.5
Filobasidium uniguttulatum	0.0	0.0	0.0	0.2	0.0	0.0	0.0	0.0	0.0	0.0	0.0	0.0	0.0	1.5	0.0	0.0	0.0	0.2	0.0	0.2	0.0	0.0	0.0	2.3	0.6
Taphrina	0.0	0.0	0.0	0.0	0.2	0.0	0.0	0.0	0.0	0.0	0.0	0.0	0.0	0.0	0.0	0.0	0.0	0.0	0.0	0.3	0.0	0.0	0.0	0.0	0.0
Acremonium	0.0	0.0	0.0	0.0	0.0	0.0	0.0	0.0	2.2	0.0	0.0	0.0	0.0	0.0	0.7	0.0	0.0	0.0	0.0	0.0	0.0	0.0	0.0	0.0	0.0
Filobasidium magnum	0.4	0.0	0.0	0.0	0.0	0.0	0.0	0.0	0.0	0.0	0.0	0.0	1.4	0.0	0.0	0.0	0.0	0.0	0.0	0.1	0.0	0.0	0.0	0.0	0.5
Vishniacozyma victoriae	0.0	0.0	0.0	0.0	0.4	0.0	0.0	0.0	0.0	0.9	0.0	0.0	0.0	0.0	0.8	0.0	0.0	0.3	0.0	0.0	0.0	0.0	0.0	1.3	0.1
Epicoccum	0.0	0.0	0.0	0.3	0.0	0.0	0.0	0.0	0.0	0.0	0.0	0.0	0.0	0.5	0.3	0.0	0.0	0.0	0.0	0.0	0.0	0.0	0.0	0.0	1.3
Cerrena	0.0	0.0	0.0	0.0	0.0	0.0	0.0	0.0	0.0	0.8	0.0	0.0	0.0	0.0	0.6	0.0	0.0	0.0	0.0	0.0	0.0	0.0	0.0	0.0	2.1
Trichosporon faecale	0.0	0.0	0.0	0.9	0.0	0.0	0.0	0.0	0.0	0.0	0.0	0.0	0.0	0.0	0.0	0.0	0.0	0.0	0.0	0.3	0.0	0.0	0.0	0.4	0.0
Trichomerium	0.0	0.0	0.0	0.0	0.0	0.0	0.0	0.0	0.0	2.1	0.0	0.0	0.0	0.0	0.0	0.0	0.0	0.4	0.0	0.0	0.0	0.0	0.0	0.0	0.0
Papiliotrema aurea	0.0	0.0	0.0	0.0	0.0	0.4	0.0	0.0	0.0	0.6	0.0	0.0	0.0	0.0	0.8	0.0	0.0	0.0	1.1	0.0	0.0	0.0	0.0	0.0	0.2
[Candida] sak	0.0	0.0	0.0	0.0	0.0	0.0	0.0	0.0	0.0	0.0	0.0	0.0	0.0	1.3	0.0	0.0	0.0	0.0	0.0	0.1	0.0	0.0	0.0	1.3	0.5
Chaetomium	0.0	0.0	0.0	0.0	0.0	0.0	0.0	0.0	0.0	0.0	0.0	0.0	0.0	0.0	0.2	0.0	0.0	0.5	0.3	1.0	0.0	0.0	0.0	0.4	0.2
Clavispora lusitaniae	0.0	0.0	0.0	0.0	0.2	0.0	0.0	0.0	0.0	0.4	0.0	0.0	0.0	0.0	0.0	0.0	0.0	0.0	0.0	1.1	0.0	0.0	0.0	0.0	0.6
Naganishia adeliensis	0.0	0.0	0.0	0.0	0.0	0.0	0.0	0.0	0.0	0.0	0.0	0.0	0.0	0.0	1.4	0.0	0.0	0.0	0.0	0.3	0.0	0.0	0.0	0.0	0.8
Naganishia albidosimilis	0.0	0.0	0.0	0.0	0.1	0.0	0.0	0.0	0.0	1.0	0.0	0.0	0.0	0.0	0.4	0.0	0.0	0.0	0.0	0.2	0.0	0.0	0.0	0.0	0.4
Naganishia albida	0.0	0.0	0.3	1.1	0.0	0.0	0.0	0.0	0.0	0.0	0.0	0.0	0.0	0.0	0.5	0.0	0.0	0.0	0.0	0.0	0.0	0.0	0.0	0.0	0.4
Naganishia diffluens	0.0	0.0	0.2	0.0	0.3	0.0	0.0	0.0	0.0	0.0	0.0	0.0	0.0	1.3	0.7	0.0	0.0	0.0	0.0	0.3	0.0	0.0	0.0	0.0	1.4
Naganishia globosa	0.0	0.0	0.4	0.0	0.0	0.0	0.0	0.0	0.0	1.0	0.0	0.0	0.0	0.0	0.0	0.0	0.0	0.0	0.0	0.1	0.0	0.0	0.0	0.0	1.8
Meyerozyma guilliermondii	0.0	0.0	0.0	0.0	0.2	0.0	0.0	0.0	0.0	0.0	0.0	0.0	0.0	0.0	0.5	0.1	0.0	0.0	0.0	0.3	0.0	0.0	0.0	0.0	1.4
Phoma	0.0	0.0	0.1	2.3	0.0	0.0	0.0	0.0	0.0	0.0	0.0	0.0	0.0	0.0	0.0	0.0	0.0	0.0	0.0	0.5	0.0	0.0	0.0	0.0	0.0
Trichosporon asahii	0.0	0.0	0.0	0.0	0.1	0.0	0.0	0.0	0.0	0.0	0.0	0.0	0.0	0.0	0.6	0.0	0.0	0.0	0.0	0.0	0.0	0.0	0.0	0.0	1.7
Trichosporon ovoides	0.0	0.0	0.0	0.4	0.0	0.0	0.0	0.0	0.0	0.0	0.0	0.0	0.0	0.0	0.2	0.0	0.0	0.0	0.0	0.0	0.0	0.0	0.0	0.0	0.0
Yarrowia lipolytica	0.0	0.0	0.0	0.1	0.1	0.0	0.0	0.0	0.0	0.7	0.0	0.0	0.0	0.5	0.3	0.0	0.0	0.0	0.0	1.1	0.0	0.0	0.0	0.0	0.0
Gyrodontium	0.0	0.0	0.0	0.0	0.3	0.0	0.0	0.0	0.0	0.0	0.0	0.0	0.0	0.0	0.0	0.0	0.0	0.0	0.0	0.3	0.0	0.0	0.0	0.0	0.0

	Subject_06					Subject_07					Subject_08					Subject_09					Subject_10				
Taxa	Baseline	Week 2	Week 4	Week 8	Week 12	Baseline	Week 2	Week 4	Week 8	Week 12	Baseline	Week 2	Week 4	Week 8	Week 12	Baseline	Week 2	Week 4	Week 8	Week 12	Baseline	Week 2	Week 4	Week 8	Week 12
Malassezia restricta	63.5	63.1	59.3	57.8	54.5	40.7	43.8	41.3	41.1	39.1	57.5	60.4	55.5	55.8	53.6	56.8	59.7	56.1	55.0	52.0	54.0	56.4	53.1	52.1	49.4
Malassezia globosa	27.5	19.9	18.7	15.2	13.5	49.3	41.2	38.7	33.9	30.9	32.6	25.6	23.5	20.2	18.4	30.3	23.3	21.9	18.0	16.0	36.0	28.6	26.9	22.9	20.6
Malassezia sympodialis	1.3	2.2	2.9	2.0	3.4	0.0	1.4	1.9	2.7	3.6	3.5	3.5	5.4	4.9	4.5	0.0	1.7	0.4	5.2	4.2	1.3	4.7	3.3	5.7	3.2
Malassezia yamatoensis	0.0	0.0	0.0	0.0	0.0	0.0	0.0	0.0	0.3	1.1	0.0	0.0	0.7	1.4	1.2	0.0	0.0	0.0	1.2	0.2	0.0	0.0	0.0	0.0	0.0
Malassezia dermatis	0.0	0.0	0.0	0.6	0.1	0.0	0.4	1.3	0.4	2.1	0.2	0.5	0.4	0.0	0.0	0.0	0.6	0.5	0.9	0.4	0.1	0.0	1.1	0.3	0.2
Malassezia japonica	0.0	0.0	0.0	0.3	0.0	0.0	0.0	0.3	0.0	0.0	0.0	0.0	0.0	0.5	0.0	0.0	0.0	0.0	0.0	0.4	0.0	0.0	0.0	0.0	0.2
Malassezia obtusa	0.0	0.0	0.0	0.0	1.4	0.0	0.0	0.0	0.0	0.5	0.0	1.1	0.4	0.5	0.4	1.4	0.3	0.3	0.4	0.3	0.0	0.0	0.0	0.0	0.0
Malassezia furfur	0.0	0.0	0.0	0.0	0.0	0.0	0.0	0.1	0.0	0.3	0.0	0.0	0.0	0.0	0.0	0.0	0.0	0.0	0.0	0.1	0.0	0.3	0.3	0.2	0.2
Malassezia slooffiae	0.0	0.0	0.0	0.2	0.3	0.0	0.0	0.4	0.0	0.0	0.0	0.2	0.0	0.2	0.4	0.0	0.0	0.2	0.4	0.0	0.0	0.0	0.1	0.1	0.2
Aureobasidium	0.0	0.0	0.0	2.0	3.3	0.0	0.0	1.5	0.4	2.1	0.0	2.1	0.0	0.0	1.4	1.8	1.1	4.3	0.0	1.1	0.6	0.5	2.1	0.4	0.9
Rhodotorula mucilaginosa	1.4	1.3	0.9	1.6	1.9	2.3	3.2	1.4	1.9	3.1	0.0	0.0	2.9	1.9	3.2	2.1	0.0	0.0	2.5	2.9	1.4	1.3	2.7	1.9	2.6
Aspergillus	2.3	3.2	0.7	2.2	3.2	2.2	2.3	1.8	2.4	3.2	2.7	0.0	1.5	0.0	0.0	2.4	3.9	1.6	2.0	0.3	1.4	1.6	0.4	0.8	2.2
Cladosporium	3.2	2.2	0.5	4.3	1.4	0.0	1.0	2.2	3.2	2.6	0.0	2.4	2.4	2.6	1.2	0.0	0.0	0.0	0.0	0.6	0.6	1.2	0.8	1.6	4.2
Torula herbarum	0.0	0.0	0.0	0.0	0.6	0.0	0.0	0.2	0.0	0.1	0.0	0.0	0.0	0.0	0.7	0.0	0.0	0.0	0.1	0.0	0.0	0.0	0.0	0.1	0.0
Naganishia friedmannii	0.0	0.0	1.4	0.5	2.2	0.0	0.0	0.3	3.2	1.2	0.0	0.0	0.0	0.0	0.2	0.0	0.0	0.0	0.4	0.5	0.0	0.4	0.3	0.4	0.2
Candida albicans	0.7	1.1	0.2	0.0	0.1	2.4	0.8	0.0	0.0	0.0	2.2	1.4	0.4	0.0	0.0	5.2	2.1	1.2	0.0	0.0	0.3	0.2	0.0	0.2	0.0
Candida parapsilosis	0.0	3.2	2.2	2.0	2.9	3.1	3.2	1.7	2.1	3.2	0.0	0.0	2.5	3.0	2.5	0.0	3.2	0.0	5.6	3.7	3.2	3.6	6.3	5.7	8.2
Alternaria	0.0	0.0	0.0	0.0	0.2	0.0	0.3	1.2	0.0	0.2	1.4	1.4	1.5	1.1	0.3	0.0	0.0	0.4	0.3	0.0	0.0	0.0	0.1	0.2	0.1
Cystobasidium slooffiae	0.0	1.1	1.4	0.0	0.1	0.0	0.0	0.0	2.1	0.1	0.0	0.0	1.0	0.3	0.0	0.0	0.2	0.4	0.6	0.3	0.7	0.0	0.2	0.0	0.0
Pichia membranifaciens	0.0	0.0	0.0	0.0	0.2	0.0	0.0	0.0	0.0	0.2	0.0	0.4	0.0	0.0	0.2	0.0	0.0	0.0	1.4	0.4	0.0	0.0	0.0	0.5	0.1
Sympodiomycopsis	0.0	0.0	0.0	0.0	0.0	0.0	1.3	0.0	0.3	0.1	0.0	0.0	0.2	0.0	0.0	0.0	0.0	0.0	0.0	0.0	0.0	0.2	0.0	0.0	0.0
[Candida] glaebosa	0.0	2.2	0.2	0.2	0.0	0.0	0.0	1.8	0.0	0.0	0.0	0.1	0.0	0.1	1.2	0.0	0.0	0.0	0.0	0.0	0.0	0.0	0.0	0.0	0.0
Filobasidium floriforme	0.0	0.0	0.0	0.3	0.0	0.0	1.1	0.0	0.0	0.0	0.0	0.0	0.0	0.0	0.0	0.0	0.0	2.4	0.0	0.0	0.0	0.0	0.2	1.7	0.4
Erythrobasidium hasegawianum	0.0	0.5	0.0	0.2	0.1	0.0	0.0	0.0	0.0	0.0	0.0	0.4	0.0	0.5	0.1	0.0	0.0	0.0	0.0	0.0	0.0	0.0	0.0	0.0	0.0
Knufia	0.0	0.0	1.1	0.4	0.0	0.0	0.0	2.1	0.0	0.2	0.0	0.0	0.0	0.3	0.0	0.0	0.0	0.0	0.2	0.0	0.0	0.0	0.0	0.1	0.0
Vishniacozyma carnescens	0.0	0.0	0.2	0.0	0.0	0.0	0.0	0.0	0.0	0.0	0.0	0.2	0.0	0.0	0.0	0.0	0.0	0.0	0.8	0.0	0.0	0.0	0.0	0.0	0.0
Cystobasidium minutum	0.0	0.0	0.0	0.0	0.1	0.0	0.0	0.0	1.3	0.3	0.0	0.0	0.3	0.5	0.3	0.0	0.0	4.3	0.0	0.0	0.0	0.4	0.0	0.0	0.0
[Candida] zeylanoides	0.0	0.0	1.3	0.0	0.2	0.0	0.0	1.8	0.0	0.0	0.0	0.3	0.0	0.0	0.0	0.0	0.0	0.0	1.0	0.2	0.0	0.0	0.3	0.2	0.1
Fusarium	0.0	0.0	1.4	2.2	0.0	0.0	0.0	0.0	0.0	0.3	0.0	0.0	0.0	0.0	0.0	0.0	0.0	0.0	1.5	0.0	0.0	0.0	0.0	0.0	0.0
Debaryomyces hansenii/fabryi/vindc	0.0	0.0	0.0	0.0	0.9	0.0	0.0	0.0	0.0	0.0	0.0	0.0	0.9	1.4	1.4	0.0	1.4	0.0	1.2	1.5	0.0	0.0	0.0	0.0	1.4
Villosiclava	0.0	0.0	0.0	1.1	0.2	0.0	0.0	0.0	0.0	0.2	0.0	0.0	0.0	0.0	0.0	0.0	0.0	0.2	0.0	0.5	0.4	0.0	0.0	0.0	0.0
Trichosporon coremiiforme	0.0	0.0	0.0	0.8	0.0	0.0	0.0	0.0	0.3	0.0	0.0	0.0	0.0	0.4	0.2	0.0	0.0	0.0	0.0	0.3	0.0	0.2	0.2	0.1	0.2
Saitozyma flava	0.0	0.0	2.1	0.0	0.0	0.0	0.0	0.0	0.0	0.0	0.0	0.0	0.1	0.0	0.0	0.0	1.4	0.0	0.0	0.1	0.0	0.0	0.0	0.0	0.0
Cystofilobasidium capitatum	0.0	0.0	1.1	0.0	0.0	0.0	0.0	0.0	0.0	0.0	0.0	0.0	0.0	0.4	0.2	0.0	1.1	0.0	0.4	0.4	0.0	0.0	0.0	0.0	0.4
Sarocladium	0.0	0.0	0.0	0.0	0.0	0.0	0.0	0.0	0.0	0.2	0.0	0.0	0.0	0.0	0.0	0.0	0.0	0.1	0.0	0.6	0.0	0.0	0.0	0.0	0.0
Penicillium	0.0	0.0	4.2	1.2	0.4	0.0	0.0	0.0	0.0	0.0	0.0	0.0	0.3	1.1	0.4	0.0	0.0	0.0	0.0	0.0	0.0	0.0	0.4	2.4	0.2
Verrucaria	0.0	0.0	0.0	0.0	0.0	0.0	0.0	0.0	0.3	0.0	0.0	0.0	0.0	0.4	0.0	0.0	0.0	0.0	0.2	0.0	0.0	0.0	0.0	0.2	0.0
Saccharomyces cerevisiae	0.0	0.0	0.2	0.0	0.0	0.0	0.0	0.0	0.0	0.1	0.0	0.0	0.1	0.0	0.2	0.0	0.0	0.0	0.0	0.2	0.0	0.1	0.0	0.0	0.0
Sterigmatomyces halophilus	0.0	0.0	0.0	1.3	0.0	0.0	0.0	0.0	0.0	0.0	0.0	0.0	0.0	0.0	0.0	0.0	0.0	0.0	0.2	0.0	0.0	0.0	0.0	0.0	0.0
Microporus	0.0	0.0	0.0	0.4	0.0	0.0	0.0	0.0	0.0	0.2	0.0	0.0	0.0	0.0	0.2	0.0	0.0	1.3	0.0	0.1	0.0	0.0	0.1	0.3	0.1
Wallemia	0.0	0.0	0.0	0.2	0.0	0.0	0.0	0.0	0.1	0.2	0.0	0.0	0.0	0.0	0.0	0.0	0.0	0.0	0.0	0.0	0.0	0.0	0.0	0.0	0.0
Papiliotrema flavescens	0.0	0.0	0.0	0.0	2.2	0.0	0.0	0.0	0.0	0.3	0.0	0.0	0.0	0.4	0.0	0.0	0.0	0.0	0.5	0.4	0.0	0.0	0.0	0.0	0.0
Filobasidium uniguttulatum	0.0	0.0	0.0	1.5	0.4	0.0	0.0	0.0	0.0	0.4	0.0	0.0	0.0	0.0	0.5	0.0	0.0	0.0	0.0	0.6	0.0	0.0	0.0	0.0	0.2
Taphrina	0.0	0.0	0.0	0.0	1.1	0.0	0.0	0.0	0.0	0.0	0.0	0.0	0.0	0.0	0.2	0.0	0.0	0.0	0.0	0.1	0.0	0.0	0.1	0.1	0.1
Acremonium	0.0	0.0	0.0	0.0	1.6	0.0	0.0	0.0	1.1	0.0	0.0	0.0	0.0	0.4	0.4	0.0	0.0	0.9	0.0	0.4	0.0	0.0	0.0	0.0	0.0
Filobasidium magnum	0.0	0.0	0.0	1.5	0.4	0.0	0.0	0.0	0.0	0.4	0.0	0.0	0.0	0.0	0.4	0.0	0.0	0.0	0.0	0.2	0.0	0.0	0.0	0.0	0.0
Vishniacozyma victoriae	0.0	0.0	0.0	0.0	0.3	0.0	0.0	0.0	0.0	0.0	0.0	0.0	0.0	0.0	0.5	0.0	0.0	0.0	0.0	0.9	0.0	0.0	0.0	0.0	0.0
Epicoccum	0.0	0.0	0.0	0.0	0.0	0.0	0.0	0.0	0.0	0.2	0.0	0.0	0.0	0.3	0.0	0.0	0.0	0.3	0.0	0.0	0.0	0.0	0.1	0.4	0.2
Cerrena	0.0	0.0	0.0	0.0	0.0	0.0	0.0	0.0	0.0	0.0	0.0	0.0	0.0	0.0	0.2	0.0	0.0	0.0	0.0	0.0	0.0	0.0	0.0	0.0	0.0
Trichosporon faecale	0.0	0.0	0.0	0.0	0.2	0.0	0.0	0.0	0.4	0.0	0.0	0.0	0.0	0.0	0.0	0.0	0.0	0.0	0.0	1.1	0.0	0.0	0.0	0.0	0.0
Trichomerium	0.0	0.0	0.0	0.0	0.0	0.0	0.0	0.0	0.0	0.2	0.0	0.0	0.0	0.1	0.0	0.0	0.0	0.0	0.0	0.0	0.0	0.0	0.0	0.0	0.0
Papiliotrema aurea	0.0	0.0	0.0	0.0	0.4	0.0	0.0	0.0	0.0	0.0	0.0	0.0	0.0	0.0	0.4	0.0	0.0	0.7	0.0	2.1	0.0	0.3	0.3	0.3	0.2
[Candida] sak	0.0	0.0	0.0	0.0	0.0	0.0	0.0	0.0	0.0	0.0	0.0	0.0	0.0	0.2	0.0	0.0	0.0	0.0	0.0	0.0	0.0	0.0	0.0	0.0	0.0
Chaetomium	0.0	0.0	0.0	0.0	0.4	0.0	0.0	0.0	0.0	0.0	0.0	0.0	0.0	0.0	0.3	0.0	0.0	0.0	0.0	0.0	0.0	0.0	0.0	0.0	0.0
Clavispora lusitaniae	0.0	0.0	0.0	0.0	0.0	0.0	0.0	0.0	1.1	0.0	0.0	0.0	0.0	0.0	0.0	0.0	0.0	0.4	0.0	0.3	0.0	0.0	0.0	0.4	0.2
Naganishia adeliensis	0.0	0.0	0.0	0.0	0.2	0.0	0.0	0.0	0.0	0.4	0.0	0.0	0.0	0.0	0.0	0.0	0.0	0.8	0.0	0.6	0.0	0.0	0.0	0.0	0.3
Naganishia albidosimilis	0.0	0.0	0.0	0.0	0.2	0.0	0.0	0.0	0.0	0.6	0.0	0.0	0.0	0.8	0.4	0.0	0.0	0.0	0.0	0.8	0.0	0.0	0.0	0.0	0.1
Naganishia albida	0.0	0.0	0.0	0.0	0.4	0.0	0.0	0.0	0.0	0.6	0.0	0.0	0.0	0.0	0.0	0.0	0.0	1.3	0.0	0.9	0.0	0.0	0.0	0.0	1.2
Naganishia diffluens	0.0	0.0	0.0	0.0	0.4	0.0	0.0	0.0	1.4	0.4	0.0	0.0	0.0	0.0	0.2	0.0	0.0	0.0	0.0	1.3	0.0	0.0	0.0	0.0	0.2
Naganishia globosa	0.0	0.0	0.0	0.0	0.1	0.0	0.0	0.0	0.0	0.6	0.0	0.0	0.0	0.3	0.4	0.0	0.0	0.0	0.0	1.6	0.0	0.0	0.4	0.3	0.3
Meyerozyma guilliermondii	0.0	0.0	0.0	0.0	0.3	0.0	0.0	0.0	0.0	0.0	0.0	0.0	0.0	0.0	0.9	0.0	0.0	0.0	0.0	1.4	0.0	0.0	0.0	0.0	1.3
Phoma	0.0	0.0	0.0	0.0	0.1	0.0	0.0	0.0	0.0	0.0	0.0	0.0	0.0	0.0	1.4	0.0	0.0	0.0	0.0	0.0	0.0	0.0	0.0	0.2	0.1
Trichosporon asahii	0.0	0.0	0.0	0.0	0.0	0.0	0.0	0.0	0.0	0.0	0.0	0.0	0.0	0.0	0.4	0.0	0.0	0.0	0.0	0.0	0.0	0.0	0.0	0.0	0.0
Trichosporon ovoides	0.0	0.0	0.0	0.0	0.1	0.0	0.0	0.0	0.0	0.4	0.0	0.0	0.0	0.0	0.2	0.0	0.0	0.0	0.0	0.0	0.0	0.0	0.1	0.1	0.2
Yarrowia lipolytica	0.0	0.0	0.0	0.0	0.0	0.0	0.0	0.0	0.0	0.1	0.0	0.0	0.0	0.0	0.3	0.0	0.0	0.0	0.0	0.0	0.0	0.0	0.1	0.1	0.1
Gyrodontium	0.0	0.0	0.0	0.0	0.0	0.0	0.0	0.0	0.0	0.0	0.0	0.0	0.0	0.0	0.5	0.0	0.0	0.0	0.0	0.0	0.0	0.0	0.0	0.0	0.0

	Subject_11					Subject_12					Subject_13					Subject_14					Subject_15				
Taxa	Baseline	Week 2	Week 4	Week 8	Week 12	Baseline	Week 2	Week 4	Week 8	Week 12	Baseline	Week 2	Week 4	Week 8	Week 12	Baseline	Week 2	Week 4	Week 8	Week 12	Baseline	Week 2	Week 4	Week 8	Week 12
Malassezia restricta	60.3	62.5	57.4	57.6	55.3	63.9	62.3	58.5	57.0	53.8	63.4	65.1	61.2	59.7	56.4	75.2	74.1	68.0	67.7	64.9	41.0	45.0	42.3	41.9	39.8
Malassezia globosa	30.7	23.5	21.6	18.4	16.7	29.1	20.8	19.5	16.0	14.2	26.6	19.9	18.8	15.3	13.6	18.6	11.9	11.0	8.3	7.1	44.6	38.0	35.7	31.1	28.2
Malassezia sympodialis	1.4	3.3	5.6	3.4	10.2	0.2	0.4	0.6	1.1	2.9	0.5	0.8	1.9	0.4	4.6	1.1	1.3	6.3	4.3	3.9	3.4	2.9	2.2	1.1	7.3
Malassezia yamatoensis	0.0	0.0	0.0	0.1	0.0	0.0	0.0	0.0	0.0	0.0	0.0	0.0	0.2	0.0	0.0	0.1	0.0	0.1	0.5	1.3	0.0	0.1	0.0	0.1	0.0
Malassezia dermatis	0.0	0.0	0.0	0.0	0.0	0.3	1.2	0.0	0.0	0.4	0.0	0.0	0.3	0.0	0.4	0.0	0.5	0.0	0.0	0.0	0.2	0.0	0.1	0.0	1.3
Malassezia japonica	0.2	0.0	0.2	0.0	0.3	0.1	0.0	0.0	0.0	0.0	1.1	0.0	0.3	0.0	0.4	0.0	0.0	0.0	0.0	0.1	0.1	0.0	0.0	0.0	0.2
Malassezia obtusa	0.0	0.1	0.0	0.0	0.0	0.0	0.4	0.4	0.8	1.4	0.0	0.0	2.1	0.0	0.3	0.0	0.0	0.0	0.9	0.7	0.0	0.3	0.2	0.1	0.2
Malassezia furfur	0.0	0.0	0.0	0.0	0.0	0.0	0.0	0.0	0.0	0.1	0.2	0.0	0.0	0.2	0.0	0.0	0.0	0.0	0.0	0.0	0.0	0.0	0.1	0.0	0.0
Malassezia slooffiae	0.0	0.2	0.0	0.3	0.0	0.0	0.4	0.4	0.0	0.0	0.0	0.0	0.2	0.0	0.0	1.1	0.0	0.0	0.4	0.7	0.0	0.2	0.0	0.0	0.0
Aureobasidium	0.0	0.0	0.0	0.0	0.5	0.0	0.2	0.0	0.0	0.0	0.0	0.0	0.0	0.0	0.0	0.0	0.0	0.0	0.0	0.0	1.1	3.2	1.3	0.2	1.0
Rhodotorula mucilaginosa	0.3	1.4	0.3	3.4	4.7	1.2	0.0	0.4	1.5	1.0	1.3	0.4	1.4	1.9	1.0	0.5	2.1	2.3	3.2	5.5	2.9	2.0	3.9	4.3	3.2
Aspergillus	1.9	2.3	2.3	2.1	0.4	0.2	1.1	0.8	3.5	2.8	0.8	1.5	2.2	1.8	0.3	0.3	1.9	0.0	0.0	0.0	1.2	0.9	3.2	0.3	0.2
Cladosporium	0.0	0.0	2.9	0.0	0.0	1.4	2.4	0.2	3.6	3.2	0.0	0.0	0.0	1.5	1.2	0.2	1.1	3.6	2.2	2.9	2.4	1.2	2.4	5.9	1.0
Torula herbarum	0.0	0.0	0.0	0.0	0.0	0.0	0.0	0.0	0.0	0.0	0.0	0.0	0.0	0.0	0.0	0.0	0.0	0.0	0.3	0.1	0.0	0.0	0.0	0.1	0.0
Naganishia friedmannii	0.3	0.3	0.1	0.1	0.3	0.2	0.4	0.0	0.1	0.0	0.1	0.3	0.5	0.2	0.9	0.0	0.0	0.0	0.0	0.2	0.0	0.0	0.0	0.0	2.1
Candida albicans	2.1	1.3	0.4	0.0	0.0	0.0	0.0	0.0	0.2	0.0	2.1	3.2	0.3	0.0	0.1	1.4	0.1	0.0	0.1	0.0	1.2	0.8	0.1	0.0	0.0
Candida parapsilosis	2.7	3.6	4.6	5.5	5.4	3.2	3.9	7.1	3.9	2.9	3.4	5.4	4.9	6.3	9.3	1.3	4.0	3.6	7.4	5.5	1.9	2.4	4.5	7.5	9.3
Alternaria	0.0	0.0	0.0	0.0	0.0	0.0	0.0	0.0	0.0	0.0	0.0	0.0	0.0	0.0	0.0	0.0	0.0	0.0	0.0	0.0	0.0	0.3	0.0	0.0	0.1
Cystobasidium slooffiae	0.0	0.0	0.0	0.0	0.0	0.0	0.0	0.3	0.0	0.4	0.0	0.0	0.0	0.2	0.1	0.0	0.0	0.3	0.8	0.3	0.0	0.0	0.0	0.2	0.0
Pichia membranifaciens	0.1	0.2	0.0	0.2	0.1	0.1	0.2	0.4	0.3	1.7	0.2	0.1	0.1	0.3	0.1	0.0	0.1	0.0	0.0	0.1	0.0	0.0	0.0	0.0	0.2
Sympodiomycopsis	0.0	0.2	0.0	0.3	0.0	0.0	0.0	0.0	0.0	0.0	0.0	1.0	0.1	0.1	0.0	0.0	0.3	0.6	0.1	0.0	0.0	0.0	0.0	0.1	0.0
[Candida] glaebosa	0.0	0.0	0.0	0.0	0.0	0.0	0.0	0.0	0.0	0.4	0.0	0.0	0.0	0.0	0.0	0.0	0.0	0.0	0.0	0.0	0.0	0.0	0.0	0.0	0.0
Filobasidium floriforme	0.0	0.0	0.1	0.0	0.0	0.0	0.0	0.0	0.2	0.0	0.0	0.0	0.0	0.2	0.3	0.0	0.0	0.0	0.1	0.0	0.0	0.0	0.2	0.0	0.1
Erythrobasidium hasegawianum	0.0	0.1	0.0	0.0	0.0	0.0	0.0	0.0	0.0	0.0	0.0	0.2	0.0	0.0	0.0	0.0	0.0	0.1	0.0	0.0	0.0	0.0	0.0	0.0	0.0
Knufia	0.0	0.0	0.0	0.2	0.1	0.0	0.0	0.0	0.0	0.1	0.0	0.0	0.1	0.1	0.1	0.0	0.0	0.0	0.0	0.1	0.0	0.0	0.0	0.1	0.0
Vishniacozyma carnescens	0.0	0.0	0.0	0.3	0.0	0.0	0.0	0.1	0.0	0.0	0.0	0.0	0.0	0.1	0.0	0.0	0.0	0.0	0.3	0.1	0.0	0.0	0.0	0.0	0.0
Cystobasidium minutum	0.0	0.0	0.0	0.0	0.2	0.0	0.0	0.0	0.0	0.0	0.0	0.0	0.1	0.0	0.0	0.0	0.0	0.1	0.0	0.0	0.0	0.3	0.0	0.2	0.1
[Candida] zeylanoides	0.0	0.0	0.2	0.0	0.3	0.0	0.1	0.0	1.4	2.9	0.0	0.0	0.2	0.0	0.3	0.1	0.2	0.4	0.0	0.1	0.0	0.0	0.0	0.0	0.1
Fusarium	0.0	0.5	0.2	0.8	0.0	0.0	0.0	0.0	0.0	0.0	0.2	0.3	0.0	4.6	0.0	0.0	0.0	1.1	0.4	0.0	0.0	0.0	1.5	0.0	1.1
Debaryomyces hansenii/fabryi/vindo	0.0	0.0	0.0	1.6	0.4	0.0	1.5	2.0	1.8	4.0	0.0	0.0	0.0	1.4	3.9	0.0	0.0	0.2	0.0	0.0	0.0	0.0	0.0	2.6	2.1
Villosiclava	0.0	0.0	0.1	0.0	0.1	0.0	0.0	0.0	0.0	0.0	0.0	0.0	0.1	0.0	0.1	0.0	0.0	0.0	0.3	0.0	0.0	0.1	0.0	0.0	0.0
Trichosporon coremiiforme	0.0	0.0	0.0	0.1	0.0	0.1	0.0	0.0	0.0	0.0	0.0	0.0	0.0	0.2	0.0	0.0	0.0	0.0	0.0	0.0	0.0	0.0	0.0	0.2	0.1
Saitozyma flava	0.0	0.1	0.0	0.0	0.1	0.0	0.0	0.0	0.0	0.0	0.0	0.1	0.0	0.0	0.1	0.0	0.0	0.2	0.1	0.1	0.0	0.0	0.0	0.1	0.1
Cystofilobasidium capitatum	0.0	0.0	0.2	0.0	0.0	0.0	1.5	0.2	0.1	0.1	0.0	0.0	0.1	0.0	0.0	0.0	2.2	0.0	0.2	0.0	0.0	0.0	0.2	0.0	0.0
Sarocladium	0.0	0.0	0.0	0.1	0.2	0.0	0.0	0.0	0.0	0.0	0.0	0.0	0.0	0.1	0.1	0.0	0.0	0.0	0.1	0.2	0.0	0.0	0.0	0.0	0.1
Penicillium	0.0	0.0	0.0	0.0	0.0	0.0	0.0	0.4	3.2	0.4	0.0	0.0	0.0	0.0	0.0	0.0	0.0	0.4	0.2	0.3	0.0	1.4	0.0	2.3	0.1
Verrucaria	0.0	0.1	0.0	0.0	0.2	0.0	0.0	0.0	0.0	0.0	0.0	0.1	0.0	0.0	0.1	0.0	0.0	0.2	0.8	0.2	0.0	0.0	0.0	0.0	0.0
Saccharomyces cerevisiae	0.0	0.0	0.1	0.0	0.1	0.0	0.0	0.0	0.0	0.0	0.0	0.1	0.1	0.0	0.1	0.0	0.0	0.1	0.1	0.2	0.0	0.0	0.1	0.0	0.1
Sterigmatomyces halophilus	0.0	0.0	0.0	0.1	0.1	0.0	0.1	0.2	0.1	0.1	0.0	0.1	0.0	0.1	0.0	0.0	0.0	0.0	0.0	0.0	0.0	0.0	0.0	0.2	0.0
Microporus	0.0	0.0	0.0	0.0	0.0	0.0	0.0	0.0	0.0	0.0	0.0	0.0	0.0	0.0	0.0	0.0	0.0	0.0	0.0	0.0	0.0	0.0	0.0	0.0	0.1
Wallemia	0.0	0.0	0.0	0.0	0.0	0.0	0.0	0.0	0.0	0.0	0.1	0.0	0.0	0.0	0.0	0.0	0.0	0.0	0.0	0.0	0.0	0.0	0.0	0.1	0.1
Papiliotrema flavescens	0.0	0.3	0.2	0.0	0.0	0.0	0.0	0.0	0.0	0.0	0.0	1.4	0.2	0.0	0.0	0.0	0.0	0.0	0.0	0.0	0.0	0.9	0.0	0.0	0.1
Filobasidium uniguttulatum	0.0	0.0	0.0	0.2	0.0	0.0	1.1	0.5	0.9	0.3	0.0	0.0	0.0	0.4	0.0	0.0	0.0	0.2	0.0	0.2	0.0	0.0	0.3	0.1	0.0
Taphrina	0.0	0.0	0.0	0.0	0.1	0.0	0.0	0.0	0.0	0.0	0.0	0.0	0.0	0.0	0.1	0.0	0.0	0.0	0.0	0.1	0.0	0.0	0.0	0.0	0.0
Acremonium	0.0	0.0	0.0	0.0	0.0	0.0	0.0	0.0	0.0	0.0	0.0	0.0	0.0	0.0	0.0	0.0	0.0	0.0	0.0	0.0	0.0	0.0	0.0	0.1	0.0
Filobasidium magnum	0.0	0.0	0.0	0.0	0.0	0.0	0.1	1.4	0.2	0.5	0.0	0.0	0.0	0.0	0.0	0.0	0.0	0.0	0.0	0.1	0.0	0.0	0.3	0.2	0.2
Vishniacozyma victoriae	0.0	0.0	0.0	0.1	0.0	0.0	0.0	0.0	0.0	0.0	0.0	0.0	0.2	0.3	0.0	0.0	0.0	0.3	0.0	0.0	0.0	0.0	0.0	0.0	0.3
Epicoccum	0.0	0.0	0.0	0.0	0.0	0.0	0.0	0.0	0.0	0.0	0.0	0.0	0.0	0.0	0.0	0.0	0.0	0.0	0.0	0.0	0.0	0.0	0.0	0.0	0.0
Cerrena	0.0	0.0	0.0	0.0	0.1	0.0	0.0	0.1	0.0	0.2	0.0	0.0	0.0	0.0	0.1	0.0	0.0	0.0	0.0	0.0	0.0	0.0	0.0	0.0	0.1
Trichosporon faecale	0.0	0.0	0.0	0.2	0.0	0.0	0.0	0.0	0.0	0.0	0.0	0.0	0.0	0.2	0.0	0.0	0.0	0.0	0.0	0.3	0.0	0.0	0.2	0.0	0.0
Trichomerium	0.0	0.0	0.0	0.0	0.0	0.0	0.0	0.0	0.0	0.0	0.0	0.0	0.0	0.0	0.0	0.0	0.0	0.4	0.0	0.0	0.0	0.0	0.0	0.0	0.0
Papiliotrema aurea	0.0	0.0	0.3	0.0	0.0	0.0	0.3	1.4	0.4	1.1	0.0	0.0	0.4	0.0	0.0	0.0	0.0	0.0	0.8	0.0	0.0	0.0	0.1	0.0	0.2
[Candida] sak	0.0	0.0	0.0	0.1	0.1	0.0	0.0	0.0	0.0	0.0	0.0	0.0	0.0	0.2	0.2	0.0	0.0	0.0	0.0	0.1	0.0	0.0	0.0	0.0	0.0
Chaetomium	0.0	0.0	0.0	0.0	0.0	0.0	0.4	0.1	0.2	0.0	0.0	0.0	0.0	0.0	0.0	0.0	0.0	0.5	0.4	1.0	0.0	0.0	0.0	0.2	0.0
Clavispora lusitaniae	0.0	0.0	1.5	2.3	0.0	0.0	0.0	0.0	0.0	0.0	0.0	0.0	1.4	1.3	0.0	0.0	0.0	0.0	0.0	1.1	0.0	0.0	1.1	0.0	0.1
Naganishia adeliensis	0.0	0.0	0.0	0.0	0.3	0.0	0.0	0.0	0.0	0.0	0.0	0.0	0.0	0.0	0.3	0.0	0.0	0.0	0.0	0.3	0.0	0.0	0.0	0.2	0.1
Naganishia albidosimilis	0.0	0.0	0.5	0.0	0.3	0.0	0.0	2.2	0.2	1.1	0.0	0.0	0.3	0.0	0.0	0.0	0.0	0.0	0.0	0.2	0.0	0.0	0.0	0.2	0.1
Naganishia albida	0.0	0.0	0.2	0.0	0.0	0.0	1.3	0.0	1.1	1.5	0.0	0.0	0.0	0.0	0.0	0.0	0.0	0.0	0.0	0.0	0.0	0.0	0.0	0.1	0.1
Naganishia diffluens	0.0	0.0	0.4	1.7	0.4	0.0	0.0	2.8	2.2	1.9	0.0	0.0	0.4	2.2	0.5	0.0	0.0	0.0	0.0	0.1	0.0	0.0	0.0	0.2	0.1
Naganishia globosa	0.0	0.0	0.2	0.0	1.4	0.0	0.0	0.0	0.0	0.0	0.0	0.0	0.7	0.0	1.3	0.0	0.0	0.0	0.0	0.1	0.0	0.0	0.0	0.0	0.0
Meyerozyma guilliermondii	0.0	0.0	0.4	0.0	0.9	0.0	0.0	0.0	0.0	0.6	0.0	0.0	1.2	0.0	1.9	0.1	0.0	0.0	0.0	0.3	0.0	0.0	0.0	0.0	0.0
Phoma	0.0	0.0	0.0	0.5	0.4	0.0	0.0	0.0	0.0	0.0	0.0	0.0	0.0	0.3	1.4	0.0	0.2	0.0	0.0	0.1	0.0	0.0	0.0	0.0	0.2
Trichosporon asahii	0.0	0.0	0.0	0.0	0.2	0.0	0.0	0.0	0.0	0.0	0.0	0.0	0.0	0.0	0.4	0.0	0.0	0.0	0.0	0.0	0.0	0.0	0.0	0.0	0.0
Trichosporon ovoides	0.0	0.0	0.0	0.0	0.0	0.0	0.0	0.0	0.0	0.0	0.0	0.0	0.0	0.0	0.0	0.0	0.0	0.0	0.0	0.0	0.0	0.0	0.0	0.0	0.1
Yarrowia lipolytica	0.0	0.0	0.0	0.3	0.0	0.0	0.0	0.0	0.0	0.0	0.0	0.0	0.0	0.4	0.0	0.0	0.0	0.0	0.0	1.1	0.0	0.0	0.0	0.0	0.0
Gyrodontium	0.0	0.0	0.0	0.0	0.1	0.0	0.0	0.0	0.0	0.0	0.0	0.0	0.0	0.0	0.0	0.0	0.0	0.0	0.0	0.3	0.0	0.0	0.0	0.0	0.0

	Subject_16					Subject_17					Subject_18					Subject_20					Subject_22				
Taxa	Baseline	Week 2	Week 4	Week 8	Week 12	Baseline	Week 2	Week 4	Week 8	Week 12	Baseline	Week 2	Week 4	Week 8	Week 12	Baseline	Week 2	Week 4	Week 8	Week 12	Baseline	Week 2	Week 4	Week 8	Week 12
Malassezia restricta	64.2	65.6	61.8	60.1	56.8	78.4	78.4	72.0	71.5	68.5	56.8	59.0	55.5	54.2	51.2	52.9	55.2	52.0	51.0	48.4	46.0	51.4	47.2	47.9	46.2
Malassezia globosa	25.8	19.4	18.2	14.9	13.2	13.5	7.6	7.0	4.5	3.5	30.7	24.0	22.5	18.8	16.8	37.1	29.8	28.0	24.0	21.6	40.3	34.6	31.8	28.1	25.8
Malassezia sympodialis	3.2	3.6	2.5	4.8	5.3	1.3	3.3	2.9	3.6	2.1	6.7	5.9	3.2	1.9	7.3	1.5	3.5	4.1	5.8	7.3	2.2	1.3	0.0	0.5	3.9
Malassezia yamatoensis	0.0	0.2	0.0	0.0	0.1	0.0	0.0	0.0	0.0	0.0	0.0	0.0	0.0	0.1	0.0	0.0	0.0	0.0	0.0	0.0	0.0	0.0	0.0	0.0	0.0
Malassezia dermatis	0.2	0.0	0.2	0.3	0.0	0.2	0.0	0.1	0.2	0.0	1.1	0.4	0.3	0.1	0.3	0.5	0.2	0.0	0.1	0.3	2.1	1.8	0.2	0.1	0.1
Malassezia japonica	0.0	0.2	0.0	0.3	0.0	0.3	0.0	0.5	0.0	0.1	0.0	0.0	0.1	0.0	0.0	0.0	0.0	0.1	0.0	0.0	0.0	0.0	0.0	0.0	0.0
Malassezia obtusa	0.0	0.0	0.0	0.3	0.1	0.0	0.3	0.0	0.0	0.0	0.2	0.0	0.0	0.1	0.1	0.0	0.1	0.2	0.0	0.4	0.0	0.0	0.1	0.0	0.1
Malassezia furfur	0.0	0.0	0.1	0.2	0.0	0.0	0.0	0.1	0.4	0.0	0.0	0.1	0.0	0.0	0.0	0.0	0.0	0.0	0.1	0.0	0.0	0.3	0.1	0.1	0.2
Malassezia slooffiae	0.2	0.0	0.0	0.1	0.1	0.0	0.2	0.0	0.0	0.0	0.1	0.0	0.0	0.0	0.1	0.1	0.0	0.0	0.0	0.2	0.0	0.0	0.1	0.3	0.3
Aureobasidium	0.0	0.3	1.3	0.3	0.0	0.0	0.0	0.0	1.4	0.0	0.2	0.1	1.4	0.2	0.0	0.0	1.2	2.5	0.0	0.0	0.4	1.4	0.3	1.5	1.3
Rhodotorula mucilaginosa	0.0	0.0	0.0	1.3	0.3	1.8	1.4	2.5	3.2	6.6	0.0	0.0	3.2	2.9	1.4	0.5	2.1	1.4	2.9	3.7	1.6	1.9	2.5	3.2	2.9
Aspergillus	0.0	0.0	1.5	2.9	1.1	1.9	0.6	3.5	1.8	1.8	0.0	1.3	0.2	3.3	1.7	1.1	0.0	2.0	0.0	1.7	0.0	0.0	3.2	1.9	0.4
Cladosporium	0.0	0.0	2.3	3.9	6.4	0.0	0.0	0.0	0.0	0.0	1.1	3.2	1.3	4.3	3.2	1.9	3.2	0.0	4.3	0.0	3.1	2.9	2.9	3.4	4.6
Torula herbarum	0.0	0.1	0.1	0.1	0.0	0.0	0.0	0.0	0.0	0.0	0.0	0.1	0.0	0.0	0.0	0.0	0.0	0.0	0.0	0.1	0.0	0.0	0.0	0.2	0.0
Naganishia friedmannii	1.1	0.0	0.0	0.2	0.0	0.1	1.6	0.1	0.4	0.2	0.0	0.0	0.1	0.2	0.1	0.0	0.0	0.2	0.0	0.0	0.0	0.1	0.2	0.2	0.3
Candida albicans	2.5	0.7	0.3	0.1	0.0	0.4	1.4	2.9	0.0	0.0	0.0	0.3	0.1	0.1	0.0	1.3	0.3	0.0	0.1	0.0	0.3	0.1	0.0	0.0	0.1
Candida parapsilosis	2.1	4.2	4.6	4.4	10.3	1.9	4.5	3.1	3.6	7.4	2.9	3.9	4.8	4.5	9.5	1.6	2.4	3.9	5.1	4.9	3.2	1.3	3.2	3.5	8.7
Alternaria	0.0	0.3	0.0	0.1	0.0	0.0	0.0	0.1	0.0	1.4	0.0	0.0	0.0	0.1	0.1	0.0	0.0	0.0	1.2	1.4	0.0	0.0	1.8	1.7	0.1
Cystobasidium slooffiae	0.3	0.0	0.0	0.0	0.2	0.0	0.0	0.0	0.0	0.1	0.0	0.1	0.0	0.0	0.2	0.1	0.0	0.0	0.8	0.0	0.3	0.0	0.0	0.0	0.0
Pichia membranifaciens	0.0	0.0	0.2	0.0	0.0	0.2	0.1	0.1	0.1	0.1	0.1	0.0	0.2	0.1	0.0	0.0	0.1	0.0	0.0	2.1	0.0	0.0	0.2	0.0	0.2
Sympodiomycopsis	0.0	0.0	0.0	0.0	0.1	0.0	0.1	0.0	0.1	0.0	0.0	0.0	0.0	0.1	0.1	0.0	0.0	0.0	0.0	0.0	0.0	0.2	0.0	0.0	0.0
[Candida] glaebosa	0.0	0.5	0.0	0.0	0.0	0.0	0.0	0.0	0.0	0.0	0.0	0.0	0.4	0.0	0.0	0.0	0.2	0.0	0.0	0.0	0.0	0.0	0.0	0.0	0.0
Filobasidium floriforme	0.0	0.0	0.3	1.1	0.1	0.0	0.0	0.0	0.0	0.0	0.0	1.2	0.0	0.2	0.3	0.2	0.0	0.0	0.3	0.0	0.0	0.0	0.1	0.2	0.2
Erythrobasidium hasegawianum	0.0	0.0	0.0	0.0	0.0	0.0	0.0	0.0	0.0	0.0	0.0	0.0	0.0	0.0	0.0	0.0	0.0	0.0	0.0	0.0	0.0	0.0	0.0	0.0	0.0
Knufia	0.0	0.4	0.1	0.0	0.0	0.0	0.1	0.0	0.1	0.1	0.0	0.0	0.0	0.0	0.1	0.0	0.0	0.0	0.0	1.1	0.0	0.0	0.0	0.1	0.0
Vishniacozyma carnescens	0.0	0.0	0.0	0.1	0.0	0.0	0.0	0.0	0.1	0.0	0.1	0.0	0.0	0.0	0.2	0.0	0.0	0.0	0.1	0.0	0.0	0.0	0.0	0.0	0.0
Cystobasidium minutum	0.2	0.0	0.0	0.0	0.0	0.0	0.0	0.0	0.0	1.1	0.0	0.0	0.0	0.0	0.0	0.0	0.2	0.1	0.0	1.3	0.0	0.3	0.0	0.0	0.0
[Candida] zeylanoides	0.0	0.0	0.3	0.0	0.0	0.0	0.0	0.1	0.0	0.3	0.0	0.0	0.0	0.0	0.2	0.0	0.0	0.0	0.1	0.0	0.0	0.0	0.2	0.2	0.2
Fusarium	0.0	1.3	0.0	1.3	0.2	0.0	0.1	0.0	1.4	0.0	0.0	0.2	3.4	3.2	1.1	0.0	1.2	0.0	0.0	0.0	0.0	1.4	0.0	0.0	0.1
Debaryomyces hansenii/fabryi/vindo	0.0	2.3	0.0	0.0	0.0	0.0	0.0	0.0	2.5	1.9	0.0	0.0	0.0	2.2	0.0	1.2	0.0	1.9	0.0	3.2	0.0	0.0	2.3	0.0	0.3
Villosiclava	0.0	0.0	0.0	0.0	0.0	0.0	0.0	0.1	0.0	0.1	0.0	0.0	0.0	0.0	0.0	0.0	0.0	0.0	0.0	0.0	0.5	0.0	0.0	0.0	0.0
Trichosporon coremiiforme	0.0	0.2	0.1	0.0	0.0	0.0	0.0	0.0	0.2	0.0	0.0	0.0	0.0	0.0	0.1	0.0	0.0	0.0	0.1	0.0	0.0	0.1	0.1	0.2	0.1
Saitozyma flava	0.0	0.0	0.0	0.1	0.2	0.0	0.1	0.0	0.0	0.2	0.0	0.0	0.0	0.0	0.0	0.0	0.0	0.0	0.0	0.0	0.0	0.3	0.0	0.0	0.0
Cystofilobasidium capitatum	0.0	0.0	0.5	0.0	0.0	0.0	0.0	0.1	0.0	0.0	0.0	0.0	0.0	0.0	0.1	0.0	0.0	0.2	0.0	1.1	0.0	0.0	0.0	0.0	0.0
Sarocladium	0.0	0.1	0.0	0.0	0.1	0.0	0.0	0.0	0.1	0.1	0.0	0.0	0.0	0.0	0.1	0.0	0.0	0.0	0.0	0.0	0.0	0.0	0.0	0.0	0.0
Penicillium	0.0	0.0	0.0	2.2	1.1	0.0	0.0	0.0	0.0	0.0	0.0	0.2	2.9	2.1	1.8	0.0	0.0	1.4	0.0	0.0	0.0	0.0	2.1	3.2	0.2
Verrucaria	0.0	0.0	0.2	0.0	0.0	0.0	0.1	0.0	0.0	0.1	0.0	0.0	0.0	0.0	0.0	0.0	0.0	0.0	0.0	0.2	0.0	0.0	0.0	0.3	0.0
Saccharomyces cerevisiae	0.0	0.2	0.0	0.0	0.0	0.0	0.0	0.1	0.0	0.1	0.0	0.0	0.0	0.0	0.0	0.0	0.0	0.0	0.0	0.0	0.0	0.1	0.0	0.0	0.0
Sterigmatomyces halophilus	0.0	0.0	0.0	0.1	0.0	0.0	0.0	0.0	0.2	0.1	0.0	0.0	0.0	0.0	0.1	0.0	0.0	0.0	0.2	0.1	0.0	0.0	0.0	0.0	0.0
Microporus	0.0	0.0	0.0	0.0	0.2	0.0	0.0	0.0	0.0	0.0	0.0	0.0	0.0	0.0	0.0	0.0	0.0	0.0	0.0	0.0	0.0	0.0	0.1	0.1	0.1
Wallemia	0.0	0.0	0.0	0.0	0.0	0.0	0.0	0.0	0.0	0.0	0.0	0.0	0.0	0.0	0.1	0.0	0.0	0.0	0.0	0.1	0.0	0.0	0.0	0.0	0.0
Papiliotrema flavescens	0.0	0.2	0.5	0.0	0.1	0.0	0.1	0.3	0.0	0.0	0.0	0.0	0.2	0.1	0.0	0.0	0.0	0.0	0.0	0.0	0.0	0.0	0.0	0.0	0.0
Filobasidium uniguttulatum	0.0	0.0	0.8	0.2	0.0	0.0	0.0	0.0	1.2	0.0	0.0	0.0	0.0	0.0	0.2	0.0	0.1	0.3	0.3	0.0	0.0	0.0	0.0	0.0	0.0
Taphrina	0.0	0.1	0.0	0.0	0.1	0.0	0.0	0.0	0.0	0.1	0.0	0.0	0.0	0.1	0.0	0.0	0.0	0.0	0.0	0.1	0.0	0.0	0.2	0.2	0.1
Acremonium	0.0	0.0	1.3	0.0	0.0	0.0	0.0	0.0	0.0	0.0	0.0	0.0	0.0	0.2	0.0	0.0	0.0	0.0	0.1	0.0	0.0	0.0	0.0	0.0	0.0
Filobasidium magnum	0.0	0.0	2.3	0.3	0.0	0.0	0.0	0.0	0.0	0.0	0.0	0.0	0.2	0.0	0.2	0.0	0.0	0.0	0.0	0.0	0.0	0.0	0.0	0.3	0.0
Vishniacozyma victoriae	0.0	0.0	0.0	0.0	0.0	0.0	0.0	0.2	0.2	0.0	0.0	0.0	0.0	0.0	0.1	0.0	0.0	0.0	0.0	0.1	0.0	0.0	0.0	0.0	0.1
Epicoccum	0.0	0.0	0.0	0.0	0.3	0.0	0.0	0.0	0.0	0.0	0.0	0.0	0.0	0.0	0.1	0.0	0.0	0.0	0.0	0.0	0.0	0.0	0.2	0.4	0.1
Cerrena	0.0	0.0	0.0	0.0	0.0	0.0	0.0	0.0	0.0	0.1	0.0	0.0	0.0	0.0	0.0	0.0	0.0	0.0	0.0	0.1	0.0	0.0	0.0	0.0	0.0
Trichosporon faecale	0.0	0.0	0.0	0.0	0.1	0.0	0.0	0.0	0.1	0.0	0.0	0.0	0.0	0.0	0.0	0.0	0.0	0.0	0.3	0.0	0.0	0.0	0.0	0.0	0.0
Trichomerium	0.0	0.1	0.0	0.0	0.0	0.0	0.0	0.0	0.0	0.0	0.0	0.0	0.0	0.0	0.0	0.0	0.0	0.0	0.0	0.1	0.0	0.0	0.0	0.0	0.0
Papiliotrema aurea	0.0	0.0	0.0	0.0	1.2	0.0	0.0	0.4	0.0	0.0	0.0	0.0	0.0	0.0	0.0	0.0	0.0	0.0	0.0	0.0	0.0	0.0	0.3	0.2	0.3
[Candida] sak	0.0	0.0	0.0	0.0	0.0	0.0	0.0	0.0	0.3	0.2	0.0	0.0	0.0	0.0	0.0	0.0	0.0	0.0	0.0	0.0	0.0	0.0	0.0	0.0	0.0
Chaetomium	0.0	0.0	0.0	0.0	0.0	0.0	0.0	0.0	0.0	0.0	0.0	0.0	0.0	0.1	0.2	0.0	0.0	0.0	0.0	0.1	0.0	0.0	0.0	0.0	0.0
Clavispora lusitaniae	0.0	0.0	0.0	0.3	1.5	0.0	0.0	0.5	1.1	0.0	0.0	0.0	0.0	0.0	1.1	0.0	0.0	1.1	1.3	0.0	0.0	0.3	0.0	0.7	1.1
Naganishia adeliensis	0.0	0.0	0.0	0.0	0.0	0.0	0.0	0.0	0.0	1.3	0.0	0.0	0.0	0.2	0.2	0.0	0.2	0.0	0.0	0.0	0.0	0.0	0.0	0.0	0.4
Naganishia albidosimilis	0.0	0.0	0.0	0.0	0.0	0.0	0.0	0.3	0.0	0.0	0.0	0.0	0.0	0.3	0.1	0.0	0.0	0.0	0.6	0.0	0.0	0.0	0.0	0.0	0.5
Naganishia albida	0.0	0.0	0.0	0.0	0.0	0.0	0.0	0.4	0.0	0.0	0.0	0.0	0.0	0.0	0.1	0.0	0.0	0.4	0.0	0.1	0.0	0.0	0.0	0.0	0.2
Naganishia diffluens	0.0	0.0	0.3	0.0	0.2	0.0	0.0	1.3	1.2	0.4	0.0	0.0	0.0	0.3	0.2	0.0	0.0	0.0	0.2	0.0	0.0	0.0	0.0	0.0	0.4
Naganishia globosa	0.0	0.0	0.2	0.0	0.2	0.0	0.0	0.3	0.0	0.3	0.0	0.0	0.0	0.0	0.1	0.0	0.0	0.2	0.0	0.1	0.0	0.2	0.4	0.2	0.2
Meyerozyma guilliermondii	0.2	0.0	0.0	0.0	0.1	0.0	0.0	1.0	0.0	0.2	0.0	0.0	0.0	0.0	0.0	0.0	0.0	0.0	0.8	0.1	0.0	0.0	0.0	0.0	0.1
Phoma	0.0	0.0	0.0	0.0	0.1	0.0	0.0	0.0	0.3	1.4	0.0	0.0	0.0	0.0	1.1	0.0	0.0	0.0	0.0	0.0	0.0	0.0	0.0	0.4	0.1
Trichosporon asahii	0.0	0.0	0.0	0.0	0.1	0.0	0.0	0.0	0.0	0.1	0.0	0.0	0.0	0.0	0.0	0.0	0.0	0.0	0.0	0.0	0.0	0.0	0.0	0.0	0.0
Trichosporon ovoides	0.0	0.0	0.0	0.0	0.1	0.0	0.0	0.0	0.0	0.0	0.0	0.0	0.0	0.0	0.0	0.0	0.0	0.0	0.0	0.0	0.0	0.0	0.1	0.1	0.0
Yarrowia lipolytica	0.0	0.0	0.0	0.0	0.0	0.0	0.0	0.0	0.2	0.0	0.0	0.0	0.0	0.0	0.0	0.0	0.0	0.0	0.0	0.0	0.0	0.0	0.1	0.6	0.0
Gyrodontium	0.0	0.0	0.0	0.0	0.0	0.0	0.0	0.0	0.0	0.0	0.0	0.0	0.0	0.0	0.0	0.0	0.0	0.0	0.2	0.0	0.0	0.0	0.0	0.0	0.0

	Subject_23					Subject_24					Subject_25					Subject_26					Subject_27				
Taxa	Baseline	Week 2	Week 4	Week 8	Week 12	Baseline	Week 2	Week 4	Week 8	Week 12	Baseline	Week 2	Week 4	Week 8	Week 12	Baseline	Week 2	Week 4	Week 8	Week 12	Baseline	Week 2	Week 4	Week 8	Week 12
Malassezia restricta	61.8	55.6	52.2	50.9	48.0	55.8	58.2	54.8	53.8	51.0	73.2	70.3	67.0	66.8	64.0	62.3	61.2	57.5	56.0	52.9	61.9	63.8	60.0	58.6	55.4
Malassezia globosa	35.2	27.4	25.8	22.1	20.0	34.2	26.8	25.2	21.2	19.0	20.8	13.0	12.0	9.2	8.0	29.7	21.8	20.5	17.0	15.1	28.1	21.3	20.0	16.4	14.6
Malassezia sympodialis	0.3	0.9	0.2	1.1	1.9	2.1	3.1	2.1	3.2	4.7	1.8	3.2	2.9	3.2	5.4	0.0	0.0	0.4	0.9	1.6	0.0	0.0	0.5	1.1	4.5
Malassezia yamatoensis	0.0	0.0	0.0	0.0	0.2	0.0	0.0	0.0	0.0	0.0	0.0	0.0	0.0	0.0	0.0	0.0	0.0	0.0	0.0	0.0	0.0	0.0	0.0	0.0	0.0
Malassezia dermatis	0.1	0.2	0.0	0.0	0.0	0.7	0.2	0.0	0.1	0.2	0.2	0.0	0.2	0.4	0.4	0.2	0.1	0.0	0.0	0.0	0.5	0.2	0.0	0.0	0.1
Malassezia japonica	0.0	0.0	0.0	0.0	0.0	0.0	0.0	0.0	0.0	0.0	0.0	0.0	0.1	0.0	0.0	0.0	0.0	0.0	0.0	0.1	0.0	0.0	0.2	0.0	1.0
Malassezia obtusa	0.0	0.2	0.1	0.1	0.0	0.0	0.4	0.2	0.1	0.3	0.1	0.1	0.0	0.0	0.0	0.0	0.0	0.0	0.0	0.0	0.0	0.0	0.0	0.0	0.0
Malassezia furfur	0.1	0.0	0.1	0.0	0.0	0.0	0.0	0.0	0.0	0.0	0.0	0.1	0.0	0.0	0.0	0.0	0.0	0.0	0.0	0.0	0.0	0.0	0.0	0.0	0.0
Malassezia slooffiae	0.0	0.1	0.0	0.0	0.1	0.2	0.0	0.2	0.0	0.1	0.0	0.0	0.1	0.0	0.0	0.0	0.0	0.3		0.3	0.0	0.0	1.3	0.4	0.0
Aureobasidium	0.0	0.7	0.9	1.2	1.1	0.0	0.0	0.0	1.3	0.0	1.3	0.2	0.0	1.2	0.9	0.6	0.3	0.9	3.0	0.4	0.3	0.1	0.0	0.0	1.1
Rhodotorula mucilaginosa	0.2	1.4	0.8	1.4	0.9	1.3	0.0	1.1	2.3	3.2	0.5	0.4	1.7	3.2	3.6	1.9	2.2	3.2	1.9	3.2	1.4	1.9	3.5	3.2	1.9
Aspergillus	0.2	3.9	0.8	0.0	2.4	0.0	2.1	0.3	2.1	1.9	0.0	1.1	1.2	1.1	0.5	0.0	1.2	1.9	1.6	1.3	0.5	0.4	1.3	1.9	2.2
Cladosporium	0.4	1.5	2.2	4.1	6.7	2.1	3.2	2.9	3.1	5.0	0.2	2.1	3.2	3.8	4.6	3.2	4.2	5.1	3.9	5.1	0.0	2.4	0.2	5.3	0.0
Torula herbarum	0.0	0.0	0.0	0.1	0.0	0.0	0.0	0.0	0.2	0.0	0.0	0.0	0.0	0.1	0.0	0.0	0.0	0.0	0.0	0.0	0.0	0.0	0.0	0.1	0.0
Naganishia friedmannii	0.0	0.2	0.3	0.1	0.3	0.2	0.0	0.2	0.0	0,1	0.0	0.2	0.0	0.0	0.2	0.0	0.5	0.8	0.8	0.8	0.1	0.3	0.4	0.1	0.3
Candida albicans	0.2	0.3	0.5	0.0	0.0	0.0	0.0	0.2	0.0	0.0	0.3	0.7	0.2	0.0	0.0	0.5	0.3	0.1	0.0	0.1	1.3	0.8	0.3	0.2	0.0
Candida parapsilosis	1.5	2.6	4.8	5.1	5.7	3.2	2.9	6.1	5.5	4.3	1.4	3.7	5.6	6.3	6.9	1.3	2.8	3.8	4.3	3.8	3.8	2.9	4.3	5.4	3.9
Alternaria	0.0	0.0	1.1	0.0	0.4	0.0	0.0	0.0	0.0	0.0	0.0	1.3	0.0	0.2	0.1	0.0	0.2	0.0	0.0	0.0	0.0	1.1	0.0	0.0	0.0
Cystobasidium slooffiae	0.0	0.0	0.2	1.3	0.7	0.0	0.0	2.3	0.0	1.3	0.0	0.1	0.0	0.2	0.0	0.1	0.0	0.2	0.3	0.2	0.0	0.0	0.0	0.1	0.0
Pichia membranifaciens	0.0	0.0	0.3	0.0	0.2	0.1	0.2	0.1	0.2	0.4	0.2	0.0	0.0	0.0	0.1	0.0	0.0	0.1	0.0	0.1	0.2	0.3	0.1	0.1	0.2
Sympodiomycopsis	0.0	0.2	0.0	0.0	0.0	0.0	0.0	0.0	0.0	0.0	0.0	0.0	0.2	0.0	0.0	0.0	0.3	0.0	0.0	0.0	0.0	0.2	0.0	0.2	0.0
[Candida] glaebosa	0.0	0.0	0.0	0.0	0.0	0.0	0.0	0.0	0.0	0.0	0.0	0.0	0.2	0.2	0.0	0.0	0.0	0.0	0.0	0.0	0.0	0.0	0.0	0.0	0.0
Filobasidium floriforme	0.0	0.0	0.2	0.0	0.3	0.0	0.0	0.0	0.0	0.2	0.0	0.2	0.0	0.1	0.1	0.0	0.0	0.2	0.4	0.2	0.4	0.0	0.0	0.0	0.1
Erythrobasidium hasegawianum	0.0	0.0	0.0	0.0	0.0	0.0	0.0	0.0	0.0	0.0	0.0	0.0	0.0	0.0	0.0	0.0	0.0	0.0	0.0	0.0	0.0	0.0	0.0	0.0	0.0
Knufia	0.0	0.0	0.0	0.0	0.1	0.0	0.0	0.0	0.0	0.0	0.0	0.0	0.0	0.1	0.1	0.0	0.0	0.4	0.1	0.4	0.0	0.0	0.0	0.2	0.1
Vishniacozyma carnescens	0.0	0.0	0.2	0.2	0.0	0.0	0.0	0.0	0.0	0.0	0.0	0.0	0.2	0.0	0.0	0.0	0.0	0.0	0.0	0.0	0.0	0.3	0.0	0.1	0.0
Cystobasidium minutum	0.0	0.0	0.0	0.2	0.0	0.0	0.0	0.0	0.0	0.0	0.0	0.0	0.0	0.0	0.2	0.0	0.0	0.0	0.0	0.0	0.0	0.0	0.0	0.0	0.2
[Candida] zeylanoides	0.0	0.0	0.0	0.2	0.1	0.0	0.2	0.0	0.2	0.4	0.0	0.0	0.0	0.1	0.0	0.0	0.0	0.2	0.2	0.2	0.0	0.0	0.3	0.0	0.2
Fusarium	0.0	0.0	3.2	0.0	0.0	0.0	0.0	0.0	0.0	0.0	0.0	2.3	1.2	1.1	1.4	0.1	1.7	3.9	5.2	3.9	1.5	1.4	0.0	0.3	0.0
Debaryomyces hansenii/fabryi/vindo	0.0	0.0	0.0	2.3	1.9	0.0	1.3	2.3	2.3	4.2	0.0	0.0	1.1	0.0	0.0	0.1	2.2	0.0	1.4	0.0	0.0	0.0	0.0	1.3	2.3
Villosiclava	0.0	0.0	0.0	0.1	0.0	0.0	0.0	0.0	0.0	0.0	0.0	0.0	0.0	0.0	0.2	0.0	0.0	0.0	0.0	0.0	0.0	0.0	0.1	0.0	0.3
Trichosporon coremiiforme	0.0	0.0	0.0	0.1	0.2	0.1	0.0	0.0	0.0	0.0	0.0	0.0	0.2	0.0	0.0	0.0	0.0	0.0	0.1	0.0	0.0	0.0	0.0	0.2	0.0
Saitozyma flava	0.0	0.0	0.0	0.0	0.0	0.0	0.0	0.0	0.0	0.1	0.0	0.0	0.0	0.1	0.0	0.0	0.0	0.0	0.0	0.0	0.0	0.3	0.0	0.0	0.3
Cystofilobasidium capitatum	0.0	0.0	0.0	0.2	0.3	0.0	0.2	0.2	0.1	0.3	0.0	0.0	0.1	0.2	0.0	0.0	0.3	0.2	0.1	0.2	0.0	0.0	0.3	0.0	0.0
Sarocladium	0.0	0.0	0.0	0.0	0.0	0.0	0.0	0.0	0.0	0.0	0.0	0.0	0.0	0.1	0.0	0.0	0.0	0.0	0.0	0.0	0.0	0.0	0.0	0.1	0.2
Penicillium	0.0	3.0	4.7	3.2	1.9	0.0	0.0	0.4	2.3	1.2	0.0	0.9	0.0	0.0	1.4	0.0	0.4	0.3	1.1	0.3	0.0	0.0	0.0	0.0	0.0
Verrucaria	0.0	0.0	0.0	0.0	0.0	0.0	0.0	0.0	0.0	0.0	0.0	0.0	0.0	0.1	0.0	0.0	0.0	0.0	0.0	0.1	0.0	0.3	0.0	0.0	0.2
Saccharomyces cerevisiae	0.0	0.0	0.0	0.0	0.0	0.0	0.0	0.0	0.0	0.0	0.0	0.0	0.0	0.0	0.0	0.0	0.0	0.0	0.0	0.0	0.0	0.0	0.1	0.0	0.1
Sterigmatomyces halophilus	0.0	0.0	0.0	0.1	0.0	0.0	0.1	0.2	0.2	0.1	0.0	0.0	0.0	0.2	0.0	0.0	0.1	0.0	0.0	0.0	0.0	0.0	0.0	0.4	1.2
Microporus	0.0	0.0	0.0	0.1	0.0	0.0	0.0	0.0	0.0	0.0	0.0	0.0	0.2	0.0	0.2	0.0	0.0	0.0	0.0	0.0	0.0	0.0	0.0	0.0	0.0
Wallemia	0.0	1.8	0.0	0.0	2.8	0.0	0.0	0.0	0.0	0.0	0.0	0.0	0.0	0.1	0.0	0.0	0.0	0.0	0.3	0.1	0.0	0.0	0.0	0.0	0.0
Papiliotrema flavescens	0.0	0.0	0.0	0.0	0.0	0.0	0.0	0.0	0.0	0.0	0.0	0.0	1.7	0.0	0.1	0.0	0.0	0.0	0.2	0.0	0.0	0.8	0.3	0.0	0.0
Filobasidium uniguttulatum	0.0	0.0	0.2	0.0	0.4	0.0	0.2	0.3	0.2	0.1	0.0	0.0	0.0	0.2	0.0	0.0	0.0	0.0	0.3	0.2	0.0	0.0	0.0	0.4	0.0
Taphrina	0.0	0.0	0.0	0.0	0.0	0.0	0.0	0.0	0.0	0.0	0.0	0.0	0.0	0.0	0.0	0.0	0.0	0.0	0.0	0.0	0.0	0.0	0.0	0.0	0.1
Acremonium	0.0	0.0	0.0	0.0	0.0	0.0	0.0	0.0	0.0	0.0	0.0	0.0	0.0	1.1	0.0	0.0	0.0	0.0	0.0	1.2	0.0	0.0	0.0	0.0	1.1
Filobasidium magnum	0.0	0.0	0.0	0.0	0.2	0.0	0.3	0.2	0.1	0.3	0.0	0.0	0.0	0.0	0.0	0.0	0.0	0.0	0.4	0.2	0.0	0.0	0.0	0.0	0.2
Vishniacozyma victoriae	0.0	0.0	0.0	0.0	0.0	0.0	0.0	0.0	0.0	0.0	0.0	0.0	0.0	0.0	0.2	0.0	0.0	0.0	0.0	0.0	0.0	0.0	0.1	0.4	0.0
Epicoccum	0.0	0.0	0.0	1.1	0.3	0.0	0.0	0.0	0.0	0.0	0.0	0.0	0.0	0.0	0.2	0.0	0.0	0.0	0.0	1.3	0.0	0.0	0.0	0.0	1.2
Cerrena	0.0	0.0	0.0	0.0	0.0	0.0	0.0	0.1	0.0	0.1	0.0	0.0	0.0	0.0	0.0	0.0	0.0	0.0	0.0	0.0	0.0	0.0	0.0	0.0	0.8
Trichosporon faecale	0.0	0.0	0.0	0.2	0.0	0.0	0.0	0.0	0.0	0.0	0.0	0.0	0.2	0.0	0.1	0.0	0.0	0.0	0.0	0.2	0.0	0.0	0.0	0.3	0.0
Trichomerium	0.0	0.0	0.0	0.0	0.0	0.0	0.0	0.0	0.0	0.0	0.0	0.0	0.0	0.0	0.0	0.0	0.0	0.0	0.0	0.0	0.0	1.3	0.0	0.0	0.0
Papiliotrema aurea	0.0	0.0	0.0	0.0	0.4	0.0	0.3	0.2	0.2	0.4	0.0	0.0	0.3	0.2	0.0	0.0	0.0	0.0	0.0	0.2	0.0	0.0	0.3	0.0	1.2
[Candida] sak	0.0	0.0	0.0	0.0	0.0	0.0	0.0	0.0	0.0	0.0	0.0	0.1	0.0	0.0	0.2	0.0	0.0	0.0	0.0	0.0	0.0	0.0	0.0	0.4	0.3
Chaetomium	0.0	0.0	0.0	0.0	0.1	0.0	0.1	0.1	0.3	0.3	0.0	0.0	0.0	0.1	0.0	0.0	0.0	0.0	0.1	0.1	0.0	0.0	0.0	0.0	0.0
Clavispora lusitaniae	0.0	0.0	0.0	1.2	0.0	0.0	0.0	0.0	0.0	0.0	0.0	0.0	0.0	0.0	0.2	0.0	0.2	0.0	0.0	0.0	0.0	0.0	1.3	1.7	0.0
Naganishia adeliensis	0.0	0.0	1.2	0.7	1.1	0.0	0.0	0.0	0.0	0.0	0.0	0.0	0.2	0.0	0.0	0.0	0.0	0.0	0.2	0.2	0.0	0.0	0.0	0.0	1.3
Naganishia albidosimilis	0.0	0.0	0.0	0.8	0.2	0.0	0.0	0.2	0.2	0.1	0.0	0.0	0.0	0.0	0.2	0.0	0.0	0.0	0.1	1.4	0.0	0.0	2.5	0.0	0.0
Naganishia albida	0.0	0.0	0.0	0.2	0.4	0.0	0.2	0.0	0.1	0.1	0.0	0.0	0.0	0.1	0.1	0.0	0.0	0.0	0.0	1.6	0.0	0.0	0.4	0.0	0.0
Naganishia diffluens	0.0	0.0	0.0	0.0	0.1	0.0	0.0	0.1	0.7	0.1	0.0	0.0	0.0	0.0	0.1	0.0	0.0	0.0	0.1	2.5	0.0	0.0	1.8	0.4	0.5
Naganishia globosa	0.0	0.0	0.0	0.3	0.1	0.0	0.0	0.0	0.0	0.0	0.0	0.0	0.0	0.1	0.0	0.0	0.0	0.0	0.0	0.0	0.0	0.0	0.3	0.0	0.3
Meyerozyma guilliermondii	0.0	0.0	0.0	0.0	0.1	0.0	0.0	0.0	0.0	0.3	0.0	0.0	0.0	0.0	0.2	0.0	0.0	0.0	0.0	0.1	0.0	0.0	0.1	0.0	0.1
Phoma	0.0	0.0	0.0	1.3	0.3	0.0	0.0	0.0	0.0	0.2	0.0	0.0	0.0	0.1	0.0	0.0	0.0	0.0	0.0	0.4	0.0	0.0	0.0	0.5	2.4
Trichosporon asahii	0.0	0.0	0.0	0.0	0.0	0.0	0.0	0.0	0.0	0.0	0.0	0.0	0.0	0.0	0.1	0.0	0.0	0.0	0.0	0.0	0.0	0.0	0.0	0.0	0.1
Trichosporon ovoides	0.0	0.0	0.0	0.0	0.0	0.0	0.0	0.0	0.0	0.0	0.0	0.0	0.0	0.0	0.0	0.0	0.0	0.0	0.0	0.0	0.0	0.0	0.0	0.0	0.0
Yarrowia lipolytica	0.0	0.0	0.0	0.0	0.0	0.0	0.0	0.0	0.0	0.0	0.0	0.0	0.0	0.0	0.0	0.0	0.0	0.0	0.0	0.0	0.0	0.0	0.0	0.2	0.0
Gyrodontium	0.0	0.0	0.0	0.0	0.1	0.0	0.0	0.0	0.0	0.1	0.0	0.0	0.0	0.0	0.0	0.0	0.0	0.0	0.0	0.0	0.0	0.0	0.0	0.0	0.0

	Subject_28					Subject_30					Subject_31					Subject_32					Subject_33				
Taxa	Baseline	Week 2	Week 4	Week 8	Week 12	Baseline	Week 2	Week 4	Week 8	Week 12	Baseline	Week 2	Week 4	Week 8	Week 12	Baseline	Week 2	Week 4	Week 8	Week 12	Baseline	Week 2	Week 4	Week 8	Week 12
Malassezia restricta	58.8	62.2	57.1	57.4	55.1	62.9	59.2	55.6	54.0	50.9	46.0	49.3	46.4	46.0	43.8	65.3	63.9	58.7	58.7	56.3	41.0	49.8	46.8	46.4	44.0
Malassezia globosa	30.3	23.8	21.9	18.6	16.9	29.4	23.8	22.4	19.0	17.1	44.0	35.7	33.6	29.0	26.3	27.4	22.1	20.3	17.3	15.7	36.4	33.2	31.2	26.6	24.0
Malassezia sympodialis	1.5	3.2	2.8	3.9	2.1	1.3	3.2	2.2	4.9	3.8	0.1	0.6	3.7	1.2	2.1	0.3	1.2	1.5	2.2	3.2	8.2	7.4	3.2	8.4	10.4
Malassezia yamatoensis	0.0	0.0	0.0	0.0	0.0	0.0	0.0	0.0	0.0	0.0	0.0	0.0	0.0	0.1	0.0	0.0	0.0	0.0	0.0	0.0	0.0	0.0	0.0	0.0	0.0
Malassezia dermatis	0.0	0.4	0.2	0.0	1.0	1.2	1.1	1.3	3.2	0.2	0.0	0.2	0.0	0.0	0.0	0.0	0.2	0.0	0.0	0.0	0.0	0.4	0.0	0.0	0.3
Malassezia japonica	0.0	0.0	0.0	0.0	0.0	0.0	0.0	0.0	0.0	0.0	0.0	0.0	0.4	0.0	0.3	0.3	0.0	0.2	0.0	0.1	0.0	0.0	0.0	0.0	0.0
Malassezia obtusa	0.0	0.2	0.3	0.5	1.0	0.2	0.0	0.0	1.1	0.0	0.3	0.0	0.0	0.2	0.0	0.0	0.0	0.0	0.0	0.0	0.0	0.1	0.3	1.5	1.6
Malassezia furfur	0.0	0.0	0.0	0.0	0.1	0.0	0.0	0.1	0.0	0.0	0.0	0.1	0.0	0.0	0.0	0.2	0.0	0.2	0.0	0.0	0.0	0.0	0.0	0.0	0.0
Malassezia slooffiae	0.0	0.0	0.0	0.0	0.2	0.0	0.0	0.0	0.0	0.1	0.5	0.2	0.5	0.0	0.1	0.0	0.1	0.0	0.0	0.0	0.0	0.0	0.0	0.0	0.0
Aureobasidium	0.0	0.0	0.0	0.0	0.0	0.2	0.4	0.0	0.0	0.5	0.0	0.0	0.0	0.2	1.0	0.2	0.0	0.0	0.0	0.0	0.0	0.0	0.0	0.0	0.0
Rhodotorula mucilaginosa	0.3	0.0	1.9	0.3	0.4	0.3	3.2	3.2	3.1	5.6	1.2	3.2	0.0	1.7	1.0	1.9	2.4	3.2	1.8	1.7	2.4	0.0	0.0	4.3	0.3
Aspergillus	0.0	0.3	0.5	1.4	3.2	0.0	0.0	1.9	0.8	2.1	1.3	2.1	3.2	1.2	0.4	0.4	0.4	1.5	2.4	2.2	1.5	2.1	2.9	0.7	0.2
Cladosporium	1.2	2.6	3.7	4.5	3.7	2.9	4.5	2.6	3.9	5.5	0.8	2.1	2.9	0.0	0.0	0.0	0.0	0.0	0.0	0.0	2.4	2.8	3.2	2.0	2.1
Torula herbarum	0.0	0.0	0.0	0.0	0.0	0.0	0.0	0.0	0.0	0.0	0.0	0.0	0.0	0.0	1.1	0.0	0.2	0.0	0.0	0.0	0.0	0.0	0.2	0.0	0.0
Naganishia friedmannii	0.4	0.0	0.1	0.0	0.1	0.0	0.2	0.0	0.4	0.0	0.2	0.1	0.4	0.0	1.0	0.2	0.4	0.3	0.4	0.3	1.3	0.0	0.0	0.0	0.3
Candida albicans	0.8	0.0	0.0	0.0	0.0	0.0	0.1	0.1	0.0	0.0	0.0	0.0	0.0	0.0	0.1	1.4	1.2	0.3	0.0	0.0	3.5	0.0	0.0	0.0	0.4
Candida parapsilosis	1.4	3.6	4.5	5.5	4.9	1.4	3.2	3.1	3.2	3.9	2.5	3.5	3.2	4.2	7.3	2.1	2.4	4.3	3.6	4.2	2.7	2.5	3.8	2.1	3.9
Alternaria	0.0	0.0	0.0	0.0	0.2	0.0	0.0	0.2	0.0	0.0	0.0	0.0	0.0	0.0	1.2	0.0	0.0	0.2	0.0	0.0	0.0	0.0	0.0	0.0	0.2
Cystobasidium slooffiae	0.0	0.0	0.1	0.0	0.2	0.0	0.0	0.1	0.4	0.2	0.3	0.0	0.0	0.2	0.0	0.0	0.0	0.0	0.0	0.2	0.0	0.0	2.1	0.0	0.4
Pichia membranifaciens	2.1	0.1	0.1	0.2	0.3	0.0	0.2	0.0	0.2	0.1	0.2	0.2	0.1	0.3	0.4	0.1	2.1	0.2	0.4	0.2	0.4	0.1	0.1	0.2	0.2
Sympodiomycopsis	0.0	0.0	0.0	0.0	0.0	0.0	0.0	0.0	0.0	0.0	0.0	0.1	0.0	0.1	0.0	0.0	0.4	0.0	0.5	0.0	0.0	0.0	0.0	0.0	0.0
[Candida] glaebosa	0.0	0.0	0.0	0.0	0.0	0.2	0.1	0.1	0.4	0.0	0.0	0.0	0.0	0.0	0.3	0.0	0.0	0.2	0.0	0.0	0.0	0.0	0.0	0.2	0.0
Filobasidium floriforme	0.0	0.0	0.0	0.0	0.0	0.0	0.0	0.3	0.0	0.1	0.0	0.0	0.3	0.0	0.3	0.0	0.0	0.0	0.0	0.0	0.0	0.0	0.0	0.0	0.0
Erythrobasidium hasegawianum	0.0	0.0	0.0	0.0	0.3	0.0	0.0	0.0	0.0	0.0	0.0	0.0	0.0	0.0	0.0	0.0	1.1	0.0	0.0	0.0	0.0	0.0	0.0	0.0	0.0
Knufia	0.0	0.0	0.0	0.0	0.0	0.0	0.0	0.0	1.2	0.0	0.0	0.0	0.0	1.2	0.3	0.0	0.0	0.2	1.4	1.1	0.0	0.0	0.0	0.0	0.2
Vishniacozyma carnescens	0.0	0.0	0.0	0.0	0.1	0.0	0.1	0.2	0.0	0.2	0.0	0.0	1.2	0.3	0.0	0.0	0.0	0.0	0.5	0.0	0.0	0.0	0.0	0.1	0.1
Cystobasidium minutum	0.0	0.0	0.0	0.0	0.0	0.0	0.0	0.2	1.1	0.2	0.0	0.0	0.0	0.0	0.4	0.0	0.0	0.3	0.0	0.3	0.0	0.0	0.0	0.0	0.0
[Candida] zeylanoides	0.0	0.2	0.0	0.3	1.3	0.0	0.0	0.0	0.0	0.1	0.0	0.0	0.2	0.0	0.3	0.0	0.0	0.1	0.0	0.4	0.0	0.1	0.0	0.3	0.2
Fusarium	0.0	0.0	0.0	0.0	0.0	0.0	0.0	3.2	1.4	1.5	0.0	2.1	0.0	2.2	0.0	0.0	0.3	0.0	3.2	0.0	0.0	0.0	0.0	0.0	0.0
Debaryomyces hansenii/fabryi/vindo	0.0	2.4	2.1	1.9	3.2	0.0	0.0	0.0	2.3	3.6	0.0	0.0	0.0	3.2	1.8	0.0	0.0	0.0	2.1	3.2	0.0	0.2	2.4	2.1	2.4
Villosiclava	0.0	0.0	0.0	0.0	0.0	0.0	0.0	0.0	0.0	0.0	0.0	0.0	0.1	0.0	0.3	0.0	0.0	0.5	0.0	0.1	0.0	0.0	0.0	0.0	0.0
Trichosporon coremiiforme	3.2	0.0	0.0	0.0	0.2	0.0	0.0	0.0	0.1	0.1	0.0	0.0	0.0	0.1	0.0	0.0	0.0	0.0	0.2	0.0	0.2	0.0	0.0	0.0	0.1
Saitozyma flava	0.0	0.0	0.0	0.0	0.0	0.0	0.0	0.1	0.0	0.0	0.0	0.1	0.0	0.0	1.2	0.0	0.1	0.0	0.0	0.1	0.0	0.0	0.0	0.0	0.0
Cystofilobasidium capitatum	0.0	0.1	0.1	0.3	0.3	0.0	0.2	0.0	0.0	0.2	0.0	0.0	0.2	0.0	0.0	0.0	0.0	1.1	0.0	0.0	0.0	0.2	0.3	0.3	0.2
Sarocladium	0.0	0.0	0.0	0.0	0.0	0.0	0.0	0.0	0.1	0.0	0.1	0.0	0.0	0.0	0.1	0.2	0.0	0.0	0.1	0.2	0.0	0.0	0.0	0.0	0.0
Penicillium	0.0	0.0	2.7	3.1	2.9	0.0	0.0	2.1	0.0	0.0	0.0	0.0	0.0	0.0	2.2	0.0	0.0	0.0	0.0	0.0	0.0	0.0	2.1	1.3	0.3
Verrucaria	0.0	0.0	0.0	0.0	0.0	0.0	0.0	0.0	0.0	0.0	0.0	0.2	0.0	0.0	0.2	0.0	0.3	0.0	0.0	0.2	0.0	0.0	0.0	0.0	0.0
Saccharomyces cerevisiae	0.0	0.0	0.0	0.0	0.0	0.0	0.0	0.0	0.0	0.0	0.0	0.0	0.1	0.0	0.1	0.0	0.0	0.2	0.0	0.1	0.0	0.0	0.0	0.0	0.0
Sterigmatomyces halophilus	0.0	0.2	0.1	0.2	0.1	0.0	0.0	0.0	0.0	0.1	0.0	0.0	0.0	0.2	0.3	0.0	0.0	0.0	0.2	0.3	0.0	0.1	0.1	1.2	2.3
Microporus	0.0	0.0	0.0	0.0	0.0	0.0	0.0	0.0	0.0	0.0	0.0	0.0	0.0	0.0	0.8	0.0	0.0	0.0	0.0	0.0	0.0	0.0	0.0	0.0	0.0
Wallemia	0.0	0.0	0.0	0.0	0.0	0.0	0.0	0.0	0.0	0.0	0.0	0.0	0.0	0.0	0.0	0.0	0.0	0.0	0.0	0.0	0.0	0.0	0.0	0.0	1.2
Papiliotrema flavescens	0.0	0.0	0.0	0.0	0.0	0.0	0.0	0.2	0.0	0.2	0.1	0.2	0.2	0.0	0.0	0.0	1.2	1.4	0.0	0.0	0.0	0.0	0.0	0.0	0.0
Filobasidium uniguttulatum	0.0	0.2	0.2	0.1	0.3	0.0	0.0	0.0	0.2	0.0	0.0	0.0	0.0	3.2	0.0	0.0	0.0	0.0	1.1	0.0	0.0	0.2	0.2	0.4	0.2
Taphrina	0.0	0.0	0.0	0.0	0.0	0.0	0.0	0.0	0.0	0.0	0.0	0.0	0.0	0.0	0.2	0.0	0.0	0.0	0.0	0.1	0.0	0.0	0.0	0.0	0.0
Acremonium	0.0	0.0	0.0	0.0	0.0	0.0	0.2	0.0	0.1	0.0	0.0	0.0	0.0	0.0	0.0	0.0	0.0	0.0	0.0	0.0	0.0	0.0	0.0	0.0	0.0
Filobasidium magnum	0.0	0.2	0.3	0.1	0.2	0.0	0.0	0.2	0.0	0.0	0.0	0.0	0.0	0.0	0.0	0.0	0.0	0.0	0.0	0.0	0.0	0.2	0.1	0.5	0.2
Vishniacozyma victoriae	0.0	0.0	0.0	0.0	0.0	0.0	0.0	0.1	0.0	0.2	0.0	0.0	0.1	2.1	0.0	0.0	0.0	1.1	0.2	0.0	0.0	0.0	0.0	0.0	0.0
Epicoccum	0.0	0.0	0.0	0.0	0.0	0.0	0.2	0.0	0.0	0.3	0.0	0.0	0.0	0.0	0.0	0.0	0.0	0.0	0.0	0.3	0.0	0.0	0.0	0.0	0.0
Cerrena	0.0	0.0	0.1	0.0	0.1	0.0	0.0	0.0	0.0	0.0	0.0	0.0	0.0	0.0	0.3	0.0	0.0	0.0	0.0	1.3	0.0	0.0	0.3	0.0	3.2
Trichosporon faecale	0.0	0.0	0.0	0.0	0.0	0.0	0.0	0.0	0.0	0.1	0.0	0.0	0.0	0.2	0.0	0.0	0.0	0.0	0.2	0.0	0.0	0.0	0.0	0.0	0.0
Trichomerium	0.0	0.0	0.0	0.0	0.0	0.0	0.0	0.0	0.0	0.0	0.0	0.0	0.0	0.0	0.0	0.0	0.0	0.0	0.0	0.0	0.0	0.0	0.0	0.0	0.0
Papiliotrema aurea	0.0	0.1	0.2	0.2	0.3	0.0	0.0	0.0	0.0	0.4	0.0	0.0	0.4	0.0	0.0	0.0	0.0	0.4	0.0	0.0	0.0	0.2	0.3	0.2	0.3
[Candida] sak	0.0	0.0	0.0	0.0	0.0	0.0	0.0	0.0	0.0	0.0	0.0	0.0	0.0	0.2	0.3	0.0	0.0	0.0	0.3	0.2	0.0	0.0	0.0	0.0	0.0
Chaetomium	0.0	0.1	0.3	0.2	0.1	0.0	0.1	0.2	0.0	0.0	0.0	0.0	0.0	0.0	0.0	0.0	0.0	0.0	0.0	0.0	0.0	0.2	0.2	0.5	0.1
Clavispora lusitaniae	0.0	0.0	0.0	0.0	0.0	0.0	0.0	0.0	0.0	1.1	0.0	0.0	1.3	1.2	0.0	0.0	0.0	1.1	2.2	0.0	0.0	0.0	0.0	0.0	0.0
Naganishia adeliensis	0.0	0.0	0.0	0.0	0.0	0.0	0.0	0.2	0.0	0.2	2.4	0.0	0.0	0.0	0.8	0.0	0.0	0.0	0.0	0.5	0.0	0.0	0.0	0.0	0.0
Naganishia albidosimilis	0.0	0.0	0.2	1.1	0.2	0.0	0.0	0.0	0.3	0.3	0.0	0.0	0.2	0.0	0.0	0.0	0.0	0.4	0.0	0.0	0.0	0.0	0.1	0.2	0.1
Naganishia albida	0.0	0.1	0.0	0.1	0.2	0.0	0.0	0.0	0.2	0.2	0.0	0.0	0.3	0.0	0.0	0.0	0.0	0.6	0.0	0.0	0.0	0.2	0.0	0.2	0.2
Naganishia diffluens	0.0	0.0	0.5	0.1	0.3	0.0	0.0	0.0	0.0	0.3	0.0	0.0	0.4	1.3	0.9	0.0	0.0	0.3	0.5	3.4	0.0	0.0	0.1	0.3	0.1
Naganishia globosa	0.0	0.0	0.0	0.0	0.0	0.0	0.0	0.1	0.0	0.0	0.0	0.0	0.1	0.0	0.2	0.0	0.0	0.3	0.0	2.4	0.0	0.0	0.0	0.0	0.0
Meyerozyma guilliermondii	0.0	0.0	0.0	0.0	0.2	0.0	0.0	0.0	0.2	0.0	0.0	0.0	0.5	0.0	1.4	0.0	0.0	0.9	0.0	0.3	0.0	0.0	0.0	0.0	0.0
Phoma	0.0	0.0	0.0	0.0	0.1	0.0	0.0	0.0	0.0	0.6	0.0	0.0	0.0	0.1	1.2	0.0	0.0	0.0	0.4	1.2	0.0	0.0	0.0	0.0	0.3
Trichosporon asahii	0.0	0.0	0.0	0.0	0.2	0.0	0.0	0.0	0.0	0.0	0.0	0.0	0.0	0.0	0.1	0.0	0.0	0.0	0.0	0.2	0.0	0.0	0.0	0.0	0.0
Trichosporon ovoides	0.0	0.0	0.0	0.0	0.0	0.0	0.0	0.0	0.0	0.0	0.0	0.0	0.0	0.0	0.0	0.0	0.0	0.0	0.0	0.0	0.0	0.0	0.0	0.0	0.0
Yarrowia lipolytica	0.0	0.0	0.0	0.0	0.0	0.0	0.0	0.0	0.0	0.0	0.0	0.0	0.0	0.1	0.0	0.0	0.0	0.0	0.1	0.0	0.0	0.0	0.0	0.0	0.0
Gyrodontium	0.0	0.0	0.0	0.0	0.0	0.0	0.0	0.0	0.0	0.0	0.0	0.0	0.0	0.0	0.0	0.0	0.0	0.0	0.0	0.0	0.0	0.0	0.0	0.0	0.0

	Healthy control									
Taxa	Subject_01	Subject_02	Subject_03	Subject_04	Subject_05	Subject_06	Subject_07	Subject_08	Subject_09	Subject_10
Malassezia restricta	44.0	43.2	43.8	45.0	49.0	55.4	52.9	43.0	51.0	48.0
Malassezia globosa	24.0	21.0	26.3	29.0	16.9	26.0	25.0	24.0	28.0	20.0
Malassezia sympodialis	10.4	4.9	3.7	3.8	4.3	2.1	3.2	6.3	5.2	4.2
Malassezia yamatoensis	1.2	0.0	0.0	0.0	0.0	0.0	0.0	0.0	0.0	0.2
Malassezia dermatis	0.3	1.1	3.2	0.0	1.5	0.1	2.1	2.0	1.3	0.0
Malassezia japonica	0.0	0.1	0.3	0.0	0.0	1.0	0.1	0.0	0.0	1.1
Malassezia obtusa	1.6	0.0	0.2	0.2	0.2	0.0	0.0	0.0	0.3	0.0
Malassezia furfur	0.5	0.0	0.0	1.2	0.1	0.0	0.0	0.0	0.0	0.0
Malassezia slooffiae	0.0	0.1	0.3	0.1	0.0	0.1	0.3	0.0	0.1	0.1
Aureobasidium	0.0	0.2	0.2	0.1	0.3	0.2	0.4	1.2	1.1	1.1
Rhodotorula mucilaginosa	0.4	4.6	1.8	2.2	0.1	1.1	1.1	4.3	1.0	0.2
Aspergillus	0.1	5.6	2.2	0.9	3.1	1.4	0.1	0.2	0.2	1.4
Cladosporium	1.8	1.3	1.9	3.1	6.3	1.1	2.1	3.5	4.3	9.2
Torula herbarum	0.0	0.1	0.1	0.1	0.2	0.2	0.1	0.1	0.1	0.1
Naganishia friedmannii	0.2	0.2	0.1	0.0	0.1	0.1	0.2	0.2	0,1	0.3
Candida albicans	0.0	0.0	0.1	0.0	0.0	0.0	0.0	0.1	0.0	0.1
Candida parapsilosis	6.0	4.6	3.0	6.4	5.5	1.2	4.5	4.3	1.1	2.1
Alternaria	0.1	0.0	0.8	0.1	0.1	0.2	0.1	0.1	0.2	0.1
Cystobasidium slooffiae	0.2	0.2	0.0	0.0	0.1	0.0	0.2	0.1	0.2	0.1
Pichia membranifaciens	0.2	0.1	0.2	0.0	0.1	3.0	0.1	0.1	0.4	0.2
Sympodiomycopsis	0.1	0.1	0.1	0.2	0.0	0.1	0.0	0.0	0.1	0.1
[Candida] glaebosa	0.2	0.0	0.4	0.0	0.0	0.0	0.1	0.3	0.0	0.0
Filobasidium floriforme	0.3	0.2	0.2	0.1	0.0	0.1	0.2	0.1	0.2	0.1
Erythrobasidium hasegawianum	0.0	0.0	0.0	0.0	0.0	0.0	0.1	0.0	0.0	0.1
Knufia	0.2	0.2	0.3	0.1	0.2	0.1	0.1	0.1	0.0	0.1
Vishniacozyma carnescens	0.1	0.2	0.0	0.1	0.1	0.0	0.1	0.3	0.1	0.1
Cystobasidium minutum	0.0	0.3	0.1	0.2	0.1	0.2	0.0	0.2	0.0	0.0
[Candida] zeylanoides	0.1	0.3	0.1	0.1	0.4	0.0	0.2	0.0	0.2	0.1
Fusarium	0.2	1.1	1.3	0.2	0.8	0.0	1.2	0.2	0.1	2.2
Debaryomyces hansenii/fabryi/vindc	1.8	2.2	2.4	2.1	2.9	1.2	0.1	0.2	1.2	1.2
Villosiclava	0.1	0.1	0.1	0.2	0.0	0.1	0.0	0.2	0.0	0.1
Trichosporon coremiiforme	0.1	0.1	0.1	0.1	0.2	0.1	0.2	0.0	0.0	0.2
Saitozyma flava	0.0	0.1	0.1	0.1	0.1	0.1	0.0	1.2	0.1	0.1
Cystofilobasidium capitatum	0.2	0.0	0.1	0.2	0.2	0.0	0.2	0.0	0.1	0.3
Sarocladium	0.2	0.1	0.1	0.2	0.0	0.0	0.0	0.0	0.0	0.0
Penicillium	1.1	0.0	3.2	0.3	1.7	0.3	0.3	0.1	1.2	0.2
Verrucaria	0.0	0.2	0.1	0.2	2.1	0.2	0.1	1.3	0.0	0.1
Saccharomyces cerevisiae	0.0	0.0	0.0	0.1	0.0	0.1	0.0	0.1	0.0	0.0
Sterigmatomyces halophilus	0.1	0.3	0.3	0.1	0.0	0.2	0.0	0.0	0.1	0.0
Microporus	0.0	0.2	0.0	0.3	0.2	0.2	0.0	0.2	0.1	0.5
Wallemia	0.2	0.1	0.3	0.0	0.2	0.2	0.0	0.1	0.0	0.2
Papiliotrema flavescens	0.0	0.3	0.2	0.2	0.0	0.0	0.1	0.1	0.0	0.0
Filobasidium uniguttulatum	0.1	0.0	0.2	0.2	0.3	0.0	0.2	0.0	0.1	0.4
Taphrina	0.0	0.1	0.2	0.0	0.1	0.1	0.0	0.1	0.0	0.2
Acremonium	0.2	0.0	0.0	0.1	0.1	0.8	0.2	0.0	0.3	0.0
Filobasidium magnum	0.2	2.1	0.4	0.0	0.2	0.2	0.2	0.0	0.0	1.1
Vishniacozyma victoriae	0.0	0.0	0.0	0.1	0.0	0.1	0.1	0.1	0.0	0.0
Epicoccum	1.1	0.3	0.0	0.2	0.2	0.2	0.9	0.2	0.1	0.3
Cerrena	0.2	1.3	0.0	0.1	0.1	0.8	0.1	0.1	0.0	0.0
Trichosporon faecale	0.0	0.0	0.1	0.1	0.2	0.1	0.2	0.1	0.0	0.0
Trichomerium	0.0	0.1	0.0	0.0	0.0	0.0	0.0	0.0	0.4	0.1
Papiliotrema aurea	0.1	0.0	0.0	0.4	0.3	0.2	0.2	0.0	0.1	0.1
[Candida] sak	0.0	0.2	0.0	0.0	0.0	0.3	0.0	0.2	0.0	0.1
Chaetomium	0.1	0.1	0.2	0.2	0.1	0.0	0.0	0.1	0.3	0.1
Clavispora lusitaniae	0.0	0.0	0.0	0.2	0.0	0.0	0.1	1.1	0.0	0.0
Naganishia adeliensis	0.3	0.2	0.2	0.1	0.2	0.1	0.2	0.0	0.0	1.0
Naganishia albidosimilis	0.2	0.1	0.2	0.1	0.2	0.0	0.2	0.1	0.1	0.2
Naganishia albida	0.1	0.2	0.0	0.1	0.3	0.0	0.3	0.1	0.1	0.2
Naganishia diffluens	0.1	1.1	0.0	0.1	0.1	0.1	1.2	0.1	0.1	0.1
Naganishia globosa	0.1	0.1	0.3	0.2	0.0	0.3	0.0	2.3	0.0	1.6
Meyerozyma guilliermondii	1.1	0.3	0.3	0.0	0.2	0.1	0.1	1.1	0.1	0.1
Phoma	0.1	0.3	0.2	0.3	0.1	0.1	0.2	0.0	0.2	0.1
Trichosporon asahii	0.0	0.0	0.0	0.0	0.2	0.1	0.0	0.1	0.0	0.0
Trichosporon ovoides	0.0	0.1	0.0	0.0	0.0	0.0	0.1	0.0	0.0	0.0
Yarrowia lipolytica	0.0	0.0	0.1	0.0	0.0	0.0	0.0	0.0	0.0	0.0
Gyrodontium	0.0	0.0	0.0	0.2	0.0	0.0	0.0	0.0	0.1	0.1
