# Supplementary material for: Dupilumab Alters Both the Bacterial and Fungal Skin Microbiomes of Patients with Atopic Dermatitis
Source: Microorganisms. 2024 Jan 22;12(1):224. doi: 10.3390/microorganisms12010224 (PMC10820602; doi:10.3390/microorganisms12010224)
Supplement: Supplementary file 1 [file microorganisms-12-00224-s001.zip › Table_S2_word.pdf]

Table S2 Relative abundance (%) of bacterial microbiome of subject involved in this study

| | Subject_01 | | | | | Subject_02 | | | | | Subject_03 | | | | | Subject_04 | | | | | Subject_05 | | | | |
|---|---|---|---|---|---|---|---|---|---|---|---|---|---|---|---|---|---|---|---|---|---|---|---|---|---|
| Taxa | Baseline | Week 2 | Week 4 | Week 8 | Week 12 | Baseline | Week 2 | Week 4 | Week 8 | Week 12 | Baseline | Week 2 | Week 4 | Week 8 | Week 12 | Baseline | Week 2 | Week 4 | Week 8 | Week 12 | Baseline | Week 2 | Week 4 | Week 8 | Week 12 |
| *Cutibacterium* | 35.1 | 33.6 | 33.9 | 35.3 | 28.1 | 6.2 | 6.9 | 8.2 | 61.3 | 61.8 | 66.4 | 67.8 | 58.9 | 91.4 | 88.3 | 25.0 | 20.6 | 45.4 | 60.7 | 65.1 | 26.1 | 23.8 | 24.6 | 25.5 | 34.5 |
| *Staphylococcus* | 53.0 | 44.0 | 46.2 | 16.9 | 16.2 | 85.6 | 76.4 | 65.3 | 16.8 | 9.4 | 24.8 | 20.1 | 14.5 | 1.5 | 1.4 | 31.6 | 23.9 | 28.6 | 4.5 | 5.3 | 32.2 | 20.7 | 20.6 | 8.0 | 12.6 |
| *Corynebacterium* | 2.7 | 3.3 | 3.8 | 26.1 | 20.4 | 3.9 | 4.3 | 5.1 | 4.1 | 3.2 | 1.4 | 1.0 | 0.7 | 0.2 | 0.1 | 14.2 | 12.2 | 3.0 | 1.9 | 2.8 | 0.9 | 0.9 | 1.8 | 8.6 | 5.7 |
| *Streptococcus* | 0.7 | 0.9 | 1.3 | 3.7 | 15.0 | 1.5 | 4.0 | 2.4 | 0.3 | 9.0 | 2.9 | 2.8 | 2.8 | 1.7 | 3.2 | 15.9 | 13.1 | 5.0 | 9.3 | 4.0 | 0.0 | 0.2 | 1.4 | 12.4 | 8.2 |
| *Snodgrassella* | 6.1 | 8.0 | 4.8 | 0.0 | 0.3 | 0.0 | 0.0 | 0.0 | 0.0 | 0.0 | 0.7 | 0.8 | 0.1 | 0.7 | 0.9 | 0.0 | 0.0 | 0.0 | 0.0 | 0.1 | 0.0 | 0.1 | 0.0 | 0.4 | 0.4 |
| *Moraxella* | 0.0 | 0.0 | 0.1 | 0.0 | 0.2 | 0.1 | 1.0 | 2.4 | 0.0 | 0.0 | 0.5 | 0.4 | 0.6 | 0.3 | 0.2 | 0.0 | 0.6 | 0.0 | 0.7 | 0.2 | 0.0 | 0.0 | 0.0 | 0.7 | 0.1 |
| *Finegoldia* | 0.2 | 0.4 | 0.7 | 0.7 | 0.1 | 0.0 | 0.0 | 0.0 | 2.6 | 2.4 | 0.1 | 0.0 | 1.1 | 0.0 | 0.1 | 1.9 | 2.3 | 1.4 | 2.5 | 2.5 | 9.1 | 8.9 | 2.7 | 3.8 | 3.0 |
| *Acinetobacter* | 0.0 | 0.0 | 0.0 | 0.2 | 0.0 | 0.0 | 0.0 | 0.0 | 0.0 | 0.1 | 0.2 | 0.4 | 0.2 | 0.0 | 0.0 | 0.0 | 0.0 | 0.0 | 0.5 | 0.1 | 0.0 | 0.0 | 0.0 | 0.1 | 0.0 |
| *Deinococcus* | 0.0 | 0.0 | 0.0 | 0.0 | 0.0 | 0.1 | 1.0 | 8.5 | 0.0 | 0.0 | 0.0 | 0.0 | 0.3 | 0.0 | 0.0 | 0.0 | 0.3 | 0.3 | 0.3 | 0.1 | 0.0 | 0.0 | 0.0 | 0.4 | 0.0 |
| *Roseomonas* | 0.0 | 0.0 | 0.0 | 0.1 | 0.0 | 0.0 | 0.0 | 0.0 | 0.0 | 0.0 | 0.0 | 0.0 | 0.0 | 0.0 | 0.0 | 0.0 | 0.2 | 0.0 | 0.0 | 0.2 | 0.1 | 0.3 | 0.2 | 0.0 | 0.0 |
| *Neisseria* | 0.0 | 1.4 | 0.8 | 0.0 | 1.5 | 0.0 | 0.2 | 0.2 | 0.0 | 0.0 | 0.5 | 0.6 | 0.9 | 0.4 | 0.2 | 0.0 | 0.6 | 0.0 | 0.6 | 0.6 | 0.0 | 0.1 | 0.0 | 0.1 | 0.1 |
| *Rothia* | 0.0 | 0.1 | 0.1 | 0.6 | 2.3 | 0.1 | 0.2 | 0.1 | 0.7 | 0.0 | 0.1 | 0.2 | 1.6 | 0.2 | 0.1 | 0.1 | 0.7 | 0.1 | 0.7 | 0.0 | 0.0 | 0.2 | 0.0 | 6.5 | 0.8 |
| *Prevotella* | 0.0 | 0.4 | 0.6 | 0.0 | 0.2 | 0.0 | 0.0 | 0.1 | 0.0 | 0.6 | 0.1 | 0.4 | 0.2 | 0.1 | 0.0 | 0.2 | 0.1 | 0.2 | 1.3 | 1.2 | 1.0 | 5.9 | 2.0 | 1.0 | 2.3 |
| *Anaerococcus* | 0.0 | 0.7 | 0.5 | 0.3 | 0.1 | 0.0 | 0.1 | 0.1 | 0.0 | 0.5 | 0.0 | 0.0 | 0.6 | 0.0 | 0.0 | 0.1 | 1.0 | 0.8 | 1.0 | 3.3 | 0.2 | 0.5 | 0.4 | 1.8 | 1.9 |
| *Kocuria* | 0.0 | 0.1 | 0.1 | 0.1 | 0.2 | 0.0 | 0.0 | 0.0 | 0.1 | 0.1 | 0.0 | 0.1 | 0.8 | 0.0 | 0.0 | 0.0 | 0.1 | 1.0 | 0.3 | 0.3 | 0.0 | 0.0 | 0.3 | 0.3 | 0.2 |
| *Haemophilus* | 0.0 | 0.0 | 0.0 | 0.0 | 0.3 | 0.0 | 0.3 | 0.2 | 0.0 | 0.0 | 0.1 | 0.3 | 1.3 | 0.1 | 0.1 | 2.2 | 1.7 | 0.7 | 1.7 | 0.1 | 0.0 | 0.0 | 0.0 | 0.0 | 0.0 |
| *Paracoccus* | 0.0 | 0.0 | 0.0 | 0.0 | 0.0 | 0.0 | 0.0 | 0.0 | 0.3 | 0.0 | 0.0 | 0.0 | 0.1 | 0.0 | 0.0 | 0.1 | 3.4 | 0.1 | 1.0 | 1.5 | 0.0 | 0.0 | 0.0 | 0.0 | 0.0 |
| *Lawsonella* | 0.0 | 0.0 | 0.0 | 0.6 | 0.5 | 0.0 | 0.0 | 0.1 | 0.7 | 1.3 | 0.1 | 0.1 | 0.5 | 0.0 | 0.0 | 0.1 | 0.2 | 1.8 | 0.3 | 1.1 | 0.0 | 0.0 | 0.5 | 0.1 | 0.2 |
| *Bacillus* | 0.3 | 0.7 | 0.8 | 1.5 | 1.0 | 0.4 | 1.3 | 1.4 | 0.2 | 0.2 | 0.1 | 0.3 | 0.5 | 0.0 | 0.0 | 0.0 | 0.5 | 0.3 | 0.1 | 0.1 | 0.1 | 0.7 | 0.6 | 0.7 | 0.3 |
| *Methylobacterium* | 0.0 | 0.0 | 0.0 | 2.5 | 0.2 | 0.0 | 0.0 | 0.0 | 0.0 | 0.0 | 0.0 | 0.0 | 0.0 | 0.0 | 0.0 | 0.0 | 0.0 | 0.0 | 0.0 | 0.0 | 18.4 | 19.6 | 18.6 | 2.0 | 2.1 |
| *Veillonella* | 0.0 | 0.0 | 0.3 | 0.0 | 1.0 | 0.0 | 0.0 | 0.0 | 0.0 | 0.4 | 0.0 | 0.0 | 1.1 | 0.0 | 0.0 | 0.0 | 0.8 | 0.0 | 0.1 | 0.0 | 0.0 | 0.1 | 0.1 | 0.4 | 1.0 |
| *Granulicatella* | 0.0 | 0.0 | 0.0 | 0.0 | 0.3 | 0.0 | 0.1 | 0.2 | 0.0 | 0.0 | 0.0 | 0.3 | 0.3 | 0.2 | 0.1 | 0.0 | 1.1 | 0.0 | 0.7 | 0.0 | 0.0 | 0.0 | 0.1 | 0.5 | 2.2 |
| *Sphingomonas* | 0.0 | 0.0 | 0.1 | 0.0 | 0.0 | 0.0 | 0.0 | 0.1 | 0.0 | 0.0 | 0.0 | 0.0 | 0.0 | 0.1 | 0.0 | 0.0 | 0.3 | 0.0 | 0.0 | 0.0 | 4.3 | 4.5 | 10.1 | 0.2 | 0.0 |
| *Williamsia* | 0.0 | 0.0 | 0.0 | 0.3 | 0.3 | 0.0 | 0.0 | 0.0 | 0.0 | 0.0 | 0.0 | 0.0 | 0.0 | 0.0 | 0.0 | 0.0 | 0.1 | 0.0 | 0.0 | 0.0 | 0.0 | 0.0 | 0.2 | 0.0 | 0.0 |
| *Peptoniphilus* | 0.1 | 0.5 | 0.3 | 0.1 | 0.0 | 0.0 | 0.0 | 0.1 | 0.8 | 0.0 | 0.0 | 0.0 | 0.5 | 0.0 | 0.0 | 0.0 | 0.0 | 0.7 | 0.5 | 1.9 | 0.2 | 2.5 | 0.4 | 0.1 | 1.3 |
| *Arachnia* | 0.0 | 0.2 | 0.2 | 0.3 | 0.2 | 0.0 | 0.0 | 0.1 | 0.4 | 0.6 | 0.2 | 0.3 | 0.5 | 0.4 | 0.3 | 0.0 | 0.1 | 1.2 | 0.3 | 0.4 | 0.0 | 0.0 | 0.1 | 0.2 | 0.3 |
| *Escherichia* | 0.0 | 0.0 | 0.0 | 0.0 | 0.0 | 0.0 | 0.0 | 0.0 | 0.0 | 0.0 | 0.0 | 0.0 | 0.0 | 0.0 | 0.0 | 0.0 | 0.0 | 0.0 | 0.0 | 0.0 | 0.0 | 0.0 | 0.0 | 0.0 | 0.0 |
| *Skermanella* | 0.0 | 0.0 | 0.1 | 0.0 | 0.0 | 0.0 | 0.0 | 0.0 | 1.4 | 0.0 | 0.0 | 0.0 | 0.0 | 0.0 | 0.0 | 0.1 | 1.2 | 1.1 | 1.5 | 2.2 | 0.0 | 0.0 | 0.2 | 0.0 | 0.0 |
| *Microcoleus* | 0.0 | 0.0 | 0.0 | 0.7 | 1.5 | 0.0 | 0.0 | 0.0 | 1.0 | 1.5 | 0.0 | 0.0 | 0.0 | 0.1 | 0.6 | 0.0 | 0.1 | 0.0 | 0.2 | 0.0 | 0.0 | 0.2 | 1.4 | 3.5 | 3.6 |
| *Serratia* | 0.0 | 0.0 | 0.1 | 0.0 | 0.0 | 0.0 | 0.0 | 0.0 | 0.0 | 0.0 | 0.0 | 0.0 | 0.7 | 0.0 | 0.0 | 0.0 | 0.0 | 0.0 | 0.3 | 0.0 | 0.0 | 0.0 | 0.0 | 0.0 | 0.3 |
| *Pseudomonas* | 0.0 | 0.0 | 0.0 | 0.2 | 0.0 | 0.0 | 0.0 | 0.0 | 0.0 | 0.0 | 0.0 | 0.1 | 0.0 | 0.0 | 0.0 | 0.0 | 0.1 | 0.0 | 0.1 | 0.0 | 0.0 | 0.1 | 0.0 | 0.0 | 0.0 |
| *Gemella* | 0.0 | 0.0 | 0.0 | 0.0 | 0.7 | 0.0 | 0.0 | 0.0 | 0.0 | 0.0 | 0.0 | 0.1 | 1.2 | 0.1 | 0.0 | 0.0 | 0.2 | 0.0 | 0.9 | 0.3 | 0.0 | 0.0 | 0.0 | 0.0 | 0.0 |
| *Porphyromonas* | 0.0 | 0.0 | 0.0 | 0.0 | 0.0 | 0.0 | 0.0 | 0.0 | 0.0 | 0.0 | 0.0 | 0.2 | 0.6 | 0.0 | 0.0 | 0.0 | 0.4 | 0.0 | 0.2 | 0.0 | 0.0 | 0.0 | 0.0 | 0.0 | 0.0 |
| *Abiotrophia* | 0.0 | 0.0 | 0.0 | 0.0 | 0.0 | 0.0 | 0.0 | 0.0 | 0.0 | 0.0 | 0.0 | 0.1 | 0.2 | 0.1 | 0.0 | 0.0 | 0.0 | 0.0 | 0.3 | 0.0 | 0.0 | 0.0 | 0.0 | 0.3 | 0.0 |
| *Mycolicibacterium* | 0.0 | 0.1 | 0.1 | 0.3 | 0.1 | 0.0 | 0.1 | 0.0 | 0.1 | 0.1 | 0.0 | 0.0 | 0.1 | 0.0 | 0.0 | 0.1 | 1.0 | 0.4 | 0.1 | 0.2 | 0.0 | 0.1 | 0.1 | 0.0 | 0.1 |
| *Enterococcus* | 0.0 | 0.1 | 0.1 | 0.1 | 0.7 | 0.0 | 0.3 | 0.2 | 0.0 | 0.1 | 0.0 | 0.1 | 0.8 | 0.0 | 0.0 | 0.0 | 0.4 | 0.0 | 0.2 | 0.1 | 0.0 | 0.0 | 0.1 | 0.3 | 0.4 |
| *Actinomyces* | 0.0 | 0.0 | 0.0 | 0.1 | 0.2 | 0.0 | 0.0 | 0.0 | 0.0 | 0.5 | 0.0 | 0.0 | 0.1 | 0.0 | 0.0 | 0.0 | 0.1 | 0.0 | 0.0 | 0.1 | 0.0 | 0.0 | 0.0 | 2.8 | 0.5 |
| *Massilia* | 0.0 | 0.0 | 0.0 | 0.0 | 0.1 | 0.0 | 0.0 | 0.0 | 0.0 | 0.0 | 0.0 | 0.0 | 0.0 | 0.0 | 0.0 | 0.0 | 0.0 | 0.0 | 0.0 | 0.0 | 0.2 | 0.6 | 0.0 | 1.3 | 0.0 |
| *Brevundimonas* | 0.0 | 0.0 | 0.0 | 0.0 | 0.1 | 0.0 | 0.0 | 0.0 | 0.3 | 0.0 | 0.0 | 0.0 | 0.0 | 0.0 | 0.0 | 0.1 | 0.5 | 0.1 | 0.2 | 0.0 | 1.9 | 2.2 | 1.2 | 0.7 | 0.0 |
| *Micrococcus* | 0.0 | 0.0 | 0.0 | 0.0 | 0.0 | 0.0 | 0.0 | 0.0 | 0.4 | 0.4 | 0.0 | 0.0 | 0.3 | 0.0 | 0.0 | 0.0 | 0.9 | 0.3 | 0.2 | 0.2 | 0.0 | 0.0 | 0.0 | 0.1 | 0.0 |
| *Kingella* | 0.0 | 0.4 | 0.3 | 0.0 | 0.0 | 0.0 | 0.0 | 0.0 | 0.0 | 0.0 | 0.0 | 0.0 | 0.1 | 0.0 | 0.0 | 0.0 | 0.0 | 0.0 | 0.0 | 0.0 | 0.0 | 0.0 | 0.0 | 0.0 | 0.0 |
| *Dermacoccus* | 0.0 | 0.0 | 0.0 | 0.0 | 0.0 | 0.0 | 0.0 | 0.0 | 0.0 | 0.0 | 0.0 | 0.0 | 0.0 | 0.0 | 0.0 | 0.0 | 0.0 | 0.0 | 0.0 | 0.0 | 0.0 | 0.0 | 0.0 | 0.0 | 0.0 |
| *Leptotrichia* | 0.0 | 0.0 | 0.0 | 0.2 | 0.4 | 0.0 | 0.0 | 0.0 | 0.0 | 0.0 | 0.0 | 0.0 | 0.5 | 0.0 | 0.0 | 0.0 | 0.5 | 0.0 | 0.1 | 0.0 | 0.0 | 0.0 | 0.0 | 0.0 | 0.0 |
| *Fusobacterium* | 0.0 | 0.0 | 0.0 | 0.1 | 0.2 | 0.0 | 0.0 | 0.0 | 0.0 | 0.0 | 0.0 | 0.0 | 0.3 | 0.0 | 0.0 | 0.0 | 0.3 | 0.0 | 0.1 | 0.1 | 0.0 | 0.0 | 0.0 | 0.2 | 0.3 |
| *Eikenella* | 0.0 | 0.4 | 0.2 | 0.0 | 0.0 | 0.0 | 0.0 | 0.0 | 0.0 | 0.0 | 0.0 | 0.0 | 0.1 | 0.0 | 0.0 | 0.0 | 0.0 | 0.0 | 0.0 | 0.0 | 0.0 | 0.0 | 0.0 | 0.0 | 0.0 |
| *Mycobacterium* | 0.0 | 0.0 | 0.0 | 0.2 | 0.2 | 0.0 | 0.0 | 0.0 | 0.1 | 0.1 | 0.0 | 0.0 | 0.1 | 0.0 | 0.1 | 0.1 | 0.2 | 0.3 | 0.1 | 0.1 | 0.0 | 0.0 | 0.0 | 0.0 | 0.0 |
| *Gordonia* | 0.0 | 0.0 | 0.0 | 0.1 | 0.1 | 0.0 | 0.0 | 0.0 | 0.1 | 0.1 | 0.0 | 0.0 | 0.0 | 0.1 | 0.0 | 0.0 | 0.1 | 0.1 | 0.0 | 0.1 | 0.0 | 0.0 | 0.1 | 0.0 | 0.0 |
| *Tessaracoccus* | 0.0 | 0.1 | 0.1 | 0.1 | 0.0 | 0.0 | 0.0 | 0.0 | 0.2 | 0.2 | 0.0 | 0.2 | 0.2 | 0.2 | 0.2 | 0.0 | 0.1 | 0.3 | 0.2 | 0.2 | 0.0 | 0.0 | 0.1 | 0.1 | 0.1 |
| *Rhodococcus* | 0.0 | 0.0 | 0.0 | 0.3 | 0.2 | 0.0 | 0.0 | 0.0 | 0.3 | 0.1 | 0.0 | 0.0 | 0.1 | 0.0 | 0.0 | 0.0 | 0.2 | 0.2 | 0.0 | 0.1 | 0.0 | 0.0 | 0.1 | 0.0 | 0.0 |
| *Metabacillus* | 0.0 | 0.1 | 0.0 | 0.0 | 0.1 | 0.0 | 0.1 | 0.0 | 0.0 | 0.0 | 0.0 | 0.0 | 0.0 | 0.0 | 0.0 | 0.0 | 0.0 | 0.0 | 0.0 | 0.0 | 0.0 | 0.0 | 0.0 | 0.0 | 0.0 |
| *Prevotellamassilia* | 0.0 | 0.0 | 0.0 | 0.0 | 0.1 | 0.0 | 0.0 | 0.1 | 0.0 | 0.0 | 0.0 | 0.1 | 0.2 | 0.0 | 0.0 | 0.0 | 0.2 | 0.1 | 0.1 | 0.0 | 0.0 | 0.0 | 0.0 | 0.0 | 0.0 |
| *Povalibacter* | 0.0 | 0.0 | 0.0 | 0.2 | 0.0 | 0.0 | 0.0 | 0.0 | 0.4 | 0.4 | 0.0 | 0.0 | 0.0 | 0.0 | 0.0 | 0.0 | 0.0 | 0.0 | 0.0 | 0.0 | 0.0 | 0.0 | 0.0 | 0.8 | 0.3 |
| *Loriellopsis* | 0.0 | 0.0 | 0.0 | 0.0 | 0.1 | 0.0 | 0.0 | 0.0 | 0.1 | 0.1 | 0.0 | 0.0 | 0.0 | 0.0 | 0.3 | 0.0 | 0.1 | 0.0 | 0.0 | 0.0 | 0.0 | 0.1 | 0.0 | 1.3 | 1.5 |
| *Microbacterium* | 0.0 | 0.0 | 0.0 | 0.0 | 0.0 | 0.0 | 0.0 | 0.0 | 0.0 | 1.0 | 0.0 | 0.0 | 0.0 | 0.0 | 0.0 | 0.0 | 0.0 | 0.0 | 0.2 | 0.0 | 0.0 | 0.0 | 0.1 | 0.0 | 0.0 |
| *Methylorubrum* | 0.0 | 0.0 | 0.0 | 0.0 | 0.0 | 0.0 | 0.0 | 0.0 | 0.0 | 0.0 | 0.0 | 0.0 | 0.0 | 0.0 | 0.0 | 0.0 | 0.0 | 0.0 | 0.0 | 0.0 | 0.6 | 1.0 | 3.7 | 0.0 | 0.0 |
| *Chryseobacterium* | 0.0 | 0.0 | 0.0 | 0.0 | 0.0 | 0.0 | 0.0 | 0.0 | 0.0 | 0.0 | 0.0 | 0.0 | 0.0 | 0.0 | 0.0 | 0.0 | 0.0 | 0.0 | 0.0 | 0.0 | 0.0 | 0.0 | 1.2 | 0.0 | 0.0 |
| *Brevibacterium* | 0.0 | 0.0 | 0.0 | 0.1 | 0.0 | 0.0 | 0.1 | 0.2 | 0.0 | 0.0 | 0.0 | 0.0 | 0.1 | 0.0 | 0.0 | 0.1 | 1.0 | 0.1 | 0.5 | 0.2 | 0.0 | 0.0 | 0.0 | 0.0 | 0.0 |
| *Nocardioides* | 0.0 | 0.1 | 0.1 | 0.1 | 0.0 | 0.0 | 0.0 | 0.0 | 0.1 | 0.1 | 0.0 | 0.1 | 0.1 | 0.2 | 0.2 | 0.0 | 0.1 | 0.2 | 0.1 | 0.2 | 0.0 | 0.0 | 0.0 | 0.0 | 0.1 |
| *Mammaliicoccus* | 0.0 | 0.4 | 0.3 | 0.1 | 0.1 | 0.1 | 0.6 | 0.4 | 0.1 | 0.1 | 0.0 | 0.1 | 0.1 | 0.0 | 0.0 | 0.0 | 0.3 | 0.1 | 0.0 | 0.0 | 0.0 | 0.2 | 0.2 | 0.0 | 0.1 |
| *Lactobacillus* | 0.0 | 0.0 | 0.3 | 0.0 | 0.0 | 0.0 | 0.0 | 0.0 | 0.0 | 0.0 | 0.0 | 0.0 | 0.0 | 0.0 | 0.0 | 0.0 | 0.3 | 0.0 | 0.1 | 0.0 | 0.0 | 0.0 | 0.0 | 0.0 | 0.0 |
| *Steroidobacter* | 0.0 | 0.0 | 0.0 | 0.2 | 0.0 | 0.0 | 0.0 | 0.1 | 0.3 | 0.3 | 0.0 | 0.0 | 0.0 | 0.0 | 0.0 | 0.0 | 0.0 | 0.0 | 0.0 | 0.0 | 0.0 | 0.0 | 0.0 | 0.5 | 0.3 |
| *Rahnella* | 0.0 | 0.0 | 0.0 | 0.0 | 0.0 | 0.0 | 0.0 | 0.0 | 0.0 | 0.0 | 0.0 | 0.0 | 0.0 | 0.0 | 0.0 | 0.0 | 0.0 | 0.0 | 0.0 | 0.0 | 0.0 | 0.0 | 0.0 | 0.0 | 0.0 |
| *Macrococcus* | 0.1 | 0.3 | 0.3 | 0.1 | 0.1 | 0.0 | 0.4 | 0.5 | 0.1 | 0.0 | 0.0 | 0.1 | 0.1 | 0.0 | 0.0 | 0.0 | 0.1 | 0.2 | 0.0 | 0.0 | 0.1 | 0.2 | 0.3 | 0.1 | 0.1 |
| *Pseudonocardia* | 0.0 | 0.0 | 0.0 | 0.1 | 0.1 | 0.0 | 0.0 | 0.0 | 0.2 | 0.1 | 0.0 | 0.1 | 0.1 | 0.1 | 0.0 | 0.0 | 0.1 | 0.2 | 0.1 | 0.1 | 0.0 | 0.0 | 0.0 | 0.1 | 0.0 |
| *Alysiella* | 0.0 | 0.1 | 0.1 | 0.0 | 0.0 | 0.0 | 0.0 | 0.0 | 0.0 | 0.0 | 0.0 | 0.0 | 0.0 | 0.0 | 0.0 | 0.0 | 0.0 | 0.0 | 0.0 | 0.0 | 0.0 | 0.0 | 0.0 | 0.0 | 0.0 |
| *Dialister* | 0.0 | 0.1 | 0.0 | 0.0 | 0.0 | 0.0 | 0.0 | 0.0 | 0.0 | 0.0 | 0.0 | 0.0 | 0.0 | 0.0 | 0.0 | 0.0 | 0.0 | 0.0 | 0.8 | 0.9 | 0.0 | 0.4 | 0.0 | 0.0 | 0.4 |
| *Psychrobacter* | 0.0 | 0.0 | 0.0 | 0.0 | 0.0 | 0.0 | 0.0 | 0.2 | 0.0 | 0.0 | 0.0 | 0.0 | 0.0 | 0.0 | 0.0 | 0.0 | 0.0 | 0.0 | 0.0 | 0.0 | 0.0 | 0.0 | 0.0 | 0.0 | 0.0 |
| *Pantoea* | 0.0 | 0.0 | 0.0 | 0.0 | 0.0 | 0.0 | 0.0 | 0.0 | 0.0 | 0.0 | 0.0 | 0.0 | 0.0 | 0.0 | 0.0 | 0.0 | 0.0 | 0.0 | 0.0 | 0.0 | 0.0 | 0.0 | 0.0 | 0.0 | 0.0 |
| *Cupriavidus* | 0.0 | 0.0 | 0.0 | 0.2 | 0.1 | 0.0 | 0.0 | 0.0 | 0.1 | 0.1 | 0.0 | 0.0 | 0.0 | 0.0 | 0.0 | 0.0 | 0.0 | 0.0 | 0.0 | 0.0 | 0.0 | 0.0 | 0.0 | 0.2 | 0.2 |
| *Simonsiella* | 0.0 | 0.3 | 0.2 | 0.0 | 0.0 | 0.0 | 0.0 | 0.0 | 0.0 | 0.0 | 0.0 | 0.0 | 0.0 | 0.0 | 0.0 | 0.0 | 0.0 | 0.0 | 0.0 | 0.0 | 0.0 | 0.0 | 0.0 | 0.1 | 0.0 |
| *Lautropia* | 0.0 | 0.0 | 0.0 | 0.0 | 0.0 | 0.0 | 0.0 | 0.0 | 0.0 | 0.0 | 0.0 | 0.0 | 0.1 | 0.0 | 0.0 | 0.0 | 0.0 | 0.0 | 0.0 | 0.0 | 0.0 | 0.0 | 0.0 | 0.0 | 0.0 |
| *Erwinia* | 0.0 | 0.0 | 0.0 | 0.0 | 0.0 | 0.0 | 0.0 | 0.0 | 0.0 | 0.0 | 0.0 | 0.0 | 0.0 | 0.0 | 0.0 | 0.0 | 0.0 | 0.0 | 0.0 | 0.0 | 0.0 | 0.0 | 0.0 | 0.0 | 0.0 |
| *Nigerium* | 0.0 | 0.1 | 0.1 | 0.1 | 0.1 | 0.0 | 0.0 | 0.0 | 0.1 | 0.1 | 0.0 | 0.1 | 0.1 | 0.1 | 0.1 | 0.0 | 0.1 | 0.1 | 0.1 | 0.1 | 0.0 | 0.0 | 0.0 | 0.1 | 0.1 |
| *Aliterella* | 0.0 | 0.0 | 0.0 | 0.0 | 0.0 | 0.0 | 0.0 | 0.0 | 0.0 | 0.0 | 0.0 | 0.0 | 0.0 | 0.0 | 0.2 | 0.0 | 0.0 | 0.0 | 0.0 | 0.0 | 0.0 | 0.1 | 0.0 | 0.5 | 0.9 |
| *Aggregatibacter* | 0.0 | 0.0 | 0.0 | 0.0 | 0.0 | 0.0 | 0.0 | 0.0 | 0.0 | 0.0 | 0.0 | 0.0 | 0.1 | 0.0 | 0.0 | 0.0 | 0.0 | 0.0 | 0.0 | 0.1 | 0.0 | 0.0 | 0.0 | 0.0 | 0.0 |
| *Dietzia* | 0.0 | 0.0 | 0.0 | 0.2 | 0.1 | 0.0 | 0.0 | 0.0 | 0.1 | 0.1 | 0.0 | 0.0 | 0.0 | 0.0 | 0.0 | 0.0 | 0.1 | 0.2 | 0.0 | 0.1 | 0.0 | 0.0 | 0.1 | 0.0 | 0.0 |
| *Bifidobacterium* | 0.0 | 0.0 | 0.0 | 0.1 | 0.0 | 0.0 | 0.0 | 0.0 | 0.0 | 0.1 | 0.0 | 0.0 | 0.0 | 0.0 | 0.0 | 0.1 | 0.3 | 0.1 | 0.2 | 0.0 | 0.0 | 0.0 | 0.0 | 0.0 | 0.0 |
| *Vitreoscilla* | 0.0 | 0.3 | 0.1 | 0.0 | 0.0 | 0.0 | 0.0 | 0.0 | 0.0 | 0.0 | 0.0 | 0.0 | 0.0 | 0.0 | 0.0 | 0.0 | 0.0 | 0.0 | 0.0 | 0.0 | 0.0 | 0.0 | 0.0 | 0.0 | 0.0 |
| *Tsukamurella* | 0.0 | 0.0 | 0.0 | 0.1 | 0.1 | 0.0 | 0.0 | 0.0 | 0.0 | 0.1 | 0.0 | 0.0 | 0.1 | 0.0 | 0.0 | 0.0 | 0.0 | 0.2 | 0.0 | 0.1 | 0.0 | 0.0 | 0.0 | 0.0 | 0.0 |
| *Schaalia* | 0.0 | 0.0 | 0.0 | 0.1 | 0.0 | 0.0 | 0.0 | 0.0 | 0.0 | 0.2 | 0.0 | 0.0 | 0.1 | 0.0 | 0.0 | 0.0 | 0.1 | 0.0 | 0.1 | 0.0 | 0.0 | 0.0 | 0.0 | 0.2 | 0.2 |
| *Gloeocapsopsis* | 0.0 | 0.0 | 0.0 | 0.2 | 0.4 | 0.0 | 0.0 | 0.0 | 0.5 | 0.3 | 0.0 | 0.0 | 0.0 | 0.0 | 0.1 | 0.0 | 0.0 | 0.0 | 0.0 | 0.0 | 0.0 | 0.0 | 0.0 | 0.4 | 0.9 |
| *Klebsiella* | 0.0 | 0.0 | 0.0 | 0.0 | 0.2 | 0.0 | 0.0 | 0.0 | 0.0 | 0.0 | 0.0 | 0.0 | 0.0 | 0.0 | 0.0 | 0.0 | 0.0 | 0.0 | 0.0 | 0.0 | 0.0 | 0.0 | 0.0 | 0.0 | 0.0 |
| *Rhizobium* | 0.0 | 0.0 | 0.0 | 0.0 | 0.0 | 0.0 | 0.0 | 0.0 | 0.0 | 0.0 | 0.0 | 0.0 | 0.0 | 0.0 | 0.0 | 0.0 | 0.0 | 0.0 | 0.0 | 0.0 | 0.0 | 0.0 | 0.1 | 0.1 | 0.0 |
| *Nocardia* | 0.0 | 0.0 | 0.0 | 0.1 | 0.1 | 0.0 | 0.0 | 0.0 | 0.0 | 0.0 | 0.0 | 0.0 | 0.0 | 0.0 | 0.0 | 0.0 | 0.1 | 0.2 | 0.0 | 0.0 | 0.0 | 0.0 | 0.0 | 0.0 | 0.0 |
| *Brachybacterium* | 0.0 | 0.0 | 0.0 | 0.1 | 0.0 | 0.0 | 0.0 | 0.0 | 0.0 | 0.0 | 0.0 | 0.0 | 0.0 | 0.0 | 0.0 | 0.0 | 0.0 | 0.1 | 0.0 | 0.0 | 0.0 | 0.0 | 0.0 | 0.0 | 0.0 |
| *Janibacter* | 0.0 | 0.0 | 0.0 | 0.0 | 0.0 | 0.0 | 0.0 | 0.0 | 0.3 | 0.0 | 0.0 | 0.0 | 0.0 | 0.0 | 0.0 | 0.0 | 0.1 | 0.0 | 0.0 | 0.0 | 0.0 | 0.0 | 0.0 | 0.0 | 0.0 |
| *Lysobacter* | 0.0 | 0.0 | 0.0 | 0.0 | 0.0 | 0.0 | 0.0 | 0.0 | 0.0 | 0.0 | 0.0 | 0.0 | 0.0 | 0.0 | 0.0 | 0.0 | 0.0 | 0.0 | 0.0 | 0.0 | 0.0 | 0.0 | 0.0 | 0.0 | 0.0 |
| *Facklamia* | 0.0 | 0.0 | 0.0 | 0.0 | 0.0 | 0.0 | 0.0 | 0.0 | 0.8 | 0.0 | 0.0 | 0.0 | 0.0 | 0.0 | 0.0 | 0.0 | 0.0 | 0.0 | 0.0 | 0.0 | 0.0 | 0.0 | 0.0 | 0.0 | 0.0 |
| *Sphingopyxis* | 0.0 | 0.0 | 0.0 | 0.0 | 0.0 | 0.0 | 0.0 | 0.0 | 0.0 | 0.0 | 0.0 | 0.0 | 0.0 | 0.0 | 0.0 | 0.0 | 0.0 | 0.0 | 0.0 | 0.0 | 0.0 | 0.0 | 0.0 | 0.0 | 0.0 |
| *Flavobacterium* | 0.0 | 0.0 | 0.0 | 0.0 | 0.2 | 0.0 | 0.0 | 0.0 | 0.8 | 0.0 | 0.0 | 0.0 | 0.0 | 0.0 | 0.0 | 0.0 | 0.0 | 0.0 | 0.0 | 0.0 | 0.2 | 1.3 | 0.4 | 0.0 | 0.0 |
| *Stenotrophomonas* | 0.0 | 0.0 | 0.0 | 0.2 | 0.0 | 0.0 | 0.0 | 0.0 | 0.0 | 0.0 | 0.0 | 0.0 | 0.0 | 0.0 | 0.0 | 0.0 | 0.0 | 0.0 | 0.0 | 0.0 | 0.0 | 0.1 | 0.0 | 0.0 | 0.0 |
| *Afipia* | 0.0 | 0.0 | 0.0 | 0.0 | 0.0 | 0.0 | 0.0 | 0.1 | 0.2 | 0.1 | 0.0 | 0.0 | 0.0 | 0.0 | 0.0 | 0.0 | 0.1 | 0.0 | 0.0 | 0.1 | 0.0 | 0.1 | 0.1 | 0.3 | 0.2 |
| *Pseudoglutamicibacter* | 0.0 | 0.0 | 0.0 | 0.0 | 0.0 | 0.0 | 0.0 | 0.0 | 0.0 | 0.0 | 0.0 | 0.0 | 0.2 | 0.0 | 0.0 | 0.0 | 0.1 | 0.2 | 0.1 | 0.1 | 0.0 | 0.0 | 0.0 | 0.2 | 0.0 |
| *Hydrobacter* | 0.0 | 0.0 | 0.0 | 0.0 | 0.0 | 0.0 | 0.0 | 0.0 | 0.0 | 0.1 | 0.0 | 0.0 | 0.0 | 0.0 | 0.0 | 0.0 | 0.0 | 0.0 | 0.0 | 0.0 | 0.0 | 0.0 | 0.0 | 0.0 | 0.2 |
| *Dermabacter* | 0.0 | 0.0 | 0.0 | 1.1 | 0.2 | 0.0 | 0.1 | 0.1 | 0.0 | 0.0 | 0.0 | 0.0 | 0.0 | 0.0 | 0.0 | 0.0 | 0.0 | 0.0 | 0.0 | 0.0 | 0.0 | 0.0 | 0.0 | 0.0 | 0.0 |
| *Pectobacterium* | 0.0 | 0.0 | 0.0 | 0.0 | 0.0 | 0.0 | 0.0 | 0.0 | 0.0 | 0.0 | 0.0 | 0.0 | 0.0 | 0.0 | 0.0 | 0.0 | 0.0 | 0.0 | 0.0 | 0.0 | 0.0 | 0.0 | 0.0 | 0.0 | 0.0 |
| *Stanieria* | 0.0 | 0.0 | 0.0 | 0.0 | 0.0 | 0.0 | 0.0 | 0.0 | 0.0 | 0.0 | 0.0 | 0.0 | 0.0 | 0.0 | 0.1 | 0.0 | 0.0 | 0.0 | 0.0 | 0.0 | 0.0 | 0.1 | 0.0 | 0.4 | 0.5 |
| *Capnocytophaga* | 0.0 | 0.0 | 0.1 | 0.0 | 0.0 | 0.0 | 0.0 | 0.0 | 0.0 | 0.0 | 0.0 | 0.0 | 0.1 | 0.0 | 0.0 | 0.0 | 0.0 | 0.0 | 0.0 | 0.0 | 0.0 | 0.0 | 0.0 | 0.0 | 0.0 |
| Other genera | 1.6 | 2.9 | 2.7 | 4.6 | 4.6 | 2.0 | 2.7 | 3.1 | 3.5 | 3.9 | 1.9 | 1.9 | 4.9 | 1.2 | 2.4 | 8.2 | 6.5 | 4.3 | 3.6 | 3.3 | 4.3 | 3.9 | 5.3 | 11.3 | 10.9 |

| | Subject_06 | | | | | Subject_07 | | | | | Subject_08 | | | | | Subject_09 | | | | | Subject_10 | | | | |
|---|---|---|---|---|---|---|---|---|---|---|---|---|---|---|---|---|---|---|---|---|---|---|---|---|---|
| Taxa | Baseline | Week 2 | Week 4 | Week 8 | Week 12 | Baseline | Week 2 | Week 4 | Week 8 | Week 12 | Baseline | Week 2 | Week 4 | Week 8 | Week 12 | Baseline | Week 2 | Week 4 | Week 8 | Week 12 | Baseline | Week 2 | Week 4 | Week 8 | Week 12 |
| *Cutibacterium* | 41.6 | 42.8 | 43.4 | 46.5 | 69.9 | 42.9 | 45.2 | 25.4 | 41.6 | 37.6 | 20.3 | 20.9 | 22.5 | 31.2 | 37.5 | 28.3 | 27.5 | 24.9 | 38.2 | 50.2 | 18.1 | 18.2 | 18.5 | 32.6 | 41.8 |
| *Staphylococcus* | 24.4 | 18.9 | 18.4 | 3.2 | 1.4 | 39.1 | 28.8 | 26.9 | 20.7 | 13.8 | 39.5 | 29.7 | 24.5 | 6.2 | 8.5 | 42.4 | 35.7 | 27.2 | 20.3 | 16.0 | 38.8 | 29.6 | 26.7 | 14.3 | 20.0 |
| *Corynebacterium* | 5.4 | 3.9 | 3.8 | 1.0 | 1.5 | 8.2 | 9.1 | 5.7 | 18.2 | 15.4 | 1.8 | 1.6 | 3.1 | 4.4 | 7.6 | 16.0 | 15.7 | 20.8 | 6.3 | 3.1 | 7.8 | 7.9 | 13.0 | 19.8 | 10.9 |
| *Streptococcus* | 6.3 | 6.6 | 4.4 | 0.6 | 1.9 | 3.8 | 4.0 | 2.1 | 1.0 | 0.9 | 1.0 | 1.0 | 0.1 | 4.7 | 3.7 | 2.6 | 2.6 | 3.2 | 5.9 | 5.1 | 9.5 | 9.5 | 6.0 | 0.7 | 0.8 |
| *Snodgrassella* | 0.3 | 0.2 | 1.0 | 1.9 | 5.3 | 0.0 | 0.0 | 0.0 | 0.1 | 0.1 | 12.2 | 13.1 | 11.1 | 16.8 | 15.8 | 0.0 | 0.1 | 0.0 | 0.1 | 0.1 | 9.9 | 10.0 | 7.9 | 12.9 | 2.8 |
| *Moraxella* | 4.2 | 4.4 | 4.4 | 0.5 | 0.3 | 0.0 | 0.3 | 0.4 | 3.0 | 6.2 | 11.3 | 15.1 | 12.6 | 4.5 | 6.2 | 4.8 | 6.6 | 9.9 | 11.5 | 6.1 | 1.9 | 0.5 | 2.7 | 3.8 | 9.0 |
| *Finegoldia* | 3.9 | 2.5 | 0.9 | 0.3 | 0.1 | 0.1 | 0.0 | 0.2 | 0.1 | 0.4 | 0.1 | 0.0 | 0.1 | 0.2 | 0.3 | 0.1 | 0.6 | 0.1 | 0.3 | 0.4 | 0.3 | 0.3 | 5.2 | 0.2 | 0.5 |
| *Acinetobacter* | 0.6 | 0.1 | 0.3 | 0.0 | 0.2 | 0.0 | 0.0 | 0.0 | 0.0 | 0.2 | 1.0 | 0.2 | 0.8 | 0.9 | 0.4 | 0.2 | 0.7 | 1.4 | 1.1 | 0.8 | 0.2 | 0.8 | 0.9 | 0.4 | 0.1 |
| *Deinococcus* | 0.0 | 0.5 | 0.1 | 0.3 | 0.0 | 0.0 | 0.0 | 0.0 | 0.1 | 0.0 | 0.0 | 0.0 | 0.0 | 0.1 | 0.0 | 0.1 | 0.0 | 0.2 | 0.3 | 0.2 | 0.1 | 0.2 | 1.1 | 0.4 | 0.1 |
| *Roseomonas* | 0.0 | 0.0 | 0.1 | 0.4 | 0.1 | 0.0 | 0.0 | 0.0 | 0.0 | 0.0 | 1.6 | 1.5 | 0.9 | 2.0 | 0.3 | 0.0 | 0.0 | 0.0 | 0.0 | 0.0 | 0.0 | 0.0 | 0.0 | 0.0 | 0.0 |
| *Neisseria* | 0.0 | 0.1 | 0.1 | 1.8 | 2.0 | 0.0 | 0.2 | 2.8 | 0.0 | 0.1 | 1.6 | 3.9 | 2.0 | 5.8 | 4.1 | 0.1 | 0.1 | 0.2 | 0.1 | 0.2 | 1.9 | 2.2 | 0.3 | 2.8 | 0.6 |
| *Rothia* | 1.1 | 0.9 | 0.1 | 0.1 | 0.1 | 0.1 | 0.1 | 0.5 | 0.1 | 0.2 | 0.0 | 0.0 | 0.0 | 0.1 | 0.6 | 0.0 | 0.2 | 0.1 | 0.4 | 0.1 | 0.5 | 0.5 | 0.2 | 0.4 | 0.1 |
| *Prevotella* | 1.4 | 1.7 | 0.3 | 0.3 | 0.1 | 0.1 | 0.3 | 1.5 | 0.2 | 2.0 | 0.0 | 0.0 | 0.0 | 0.1 | 0.0 | 0.2 | 0.2 | 0.1 | 0.9 | 0.0 | 0.0 | 0.0 | 0.5 | 0.0 | 0.7 |
| *Anaerococcus* | 0.9 | 0.7 | 0.1 | 0.2 | 0.1 | 0.0 | 0.0 | 0.0 | 0.0 | 0.3 | 0.0 | 0.0 | 0.2 | 0.3 | 0.2 | 0.2 | 0.4 | 0.1 | 0.7 | 0.1 | 2.0 | 3.8 | 4.8 | 0.2 | 1.0 |
| *Kocuria* | 0.3 | 1.7 | 4.1 | 0.9 | 0.9 | 0.1 | 0.2 | 0.3 | 0.1 | 0.2 | 0.0 | 0.0 | 0.0 | 0.0 | 0.2 | 0.1 | 0.1 | 0.0 | 0.1 | 0.1 | 0.0 | 0.1 | 0.5 | 0.1 | 0.2 |
| *Haemophilus* | 0.1 | 0.2 | 0.0 | 0.0 | 0.0 | 0.1 | 0.5 | 1.9 | 0.0 | 0.0 | 0.0 | 0.1 | 0.0 | 0.7 | 0.1 | 0.0 | 0.2 | 0.0 | 0.3 | 0.2 | 0.0 | 0.9 | 0.0 | 0.0 | 0.0 |
| *Paracoccus* | 0.1 | 0.1 | 0.1 | 0.3 | 0.0 | 0.1 | 0.9 | 2.4 | 2.7 | 2.2 | 0.1 | 0.4 | 0.3 | 0.6 | 0.1 | 0.0 | 0.0 | 0.0 | 0.0 | 0.0 | 0.0 | 0.0 | 0.0 | 0.3 | 0.1 |
| *Lawsonella* | 0.1 | 0.6 | 0.2 | 0.1 | 0.0 | 0.1 | 1.0 | 1.5 | 2.0 | 0.6 | 0.0 | 0.0 | 0.0 | 0.1 | 0.1 | 0.0 | 0.2 | 0.0 | 0.3 | 2.3 | 0.0 | 0.2 | 0.5 | 0.1 | 0.4 |
| *Bacillus* | 0.1 | 0.4 | 0.4 | 0.2 | 0.2 | 0.2 | 0.6 | 1.2 | 0.3 | 0.2 | 0.1 | 0.6 | 0.6 | 0.2 | 0.1 | 0.2 | 0.5 | 0.4 | 0.4 | 0.3 | 0.0 | 0.3 | 0.3 | 0.6 | 0.3 |
| *Methylobacterium* | 0.0 | 0.0 | 0.2 | 0.0 | 0.0 | 0.0 | 0.0 | 0.1 | 0.0 | 0.5 | 0.7 | 0.5 | 0.4 | 1.6 | 1.0 | 0.0 | 0.0 | 0.1 | 0.0 | 0.0 | 0.0 | 0.0 | 0.0 | 0.5 | 0.8 |
| *Veillonella* | 0.0 | 0.2 | 0.0 | 0.1 | 0.2 | 0.1 | 0.4 | 2.2 | 0.0 | 0.0 | 0.0 | 0.0 | 0.0 | 0.3 | 0.1 | 0.0 | 0.3 | 0.4 | 0.4 | 0.1 | 0.0 | 1.1 | 0.0 | 0.5 | 0.4 |
| *Granulicatella* | 0.0 | 0.4 | 0.0 | 0.1 | 0.2 | 0.1 | 0.5 | 1.6 | 0.1 | 0.2 | 0.0 | 0.0 | 0.0 | 0.1 | 0.1 | 0.0 | 0.4 | 0.1 | 0.3 | 0.6 | 0.0 | 0.8 | 0.0 | 0.0 | 0.1 |
| *Sphingomonas* | 0.0 | 0.0 | 0.3 | 0.6 | 0.0 | 0.2 | 0.4 | 0.4 | 0.5 | 3.8 | 0.9 | 0.4 | 0.5 | 0.6 | 0.6 | 0.1 | 0.3 | 0.9 | 1.6 | 0.3 | 0.0 | 0.0 | 0.0 | 0.2 | 0.7 |
| *Williamsia* | 0.0 | 0.1 | 0.0 | 0.0 | 0.0 | 0.0 | 0.1 | 0.5 | 0.5 | 0.4 | 0.9 | 0.8 | 0.9 | 3.9 | 0.3 | 0.0 | 0.1 | 1.1 | 0.3 | 0.2 | 0.0 | 0.0 | 0.0 | 0.0 | 0.0 |
| *Peptoniphilus* | 0.0 | 0.1 | 0.2 | 0.1 | 0.1 | 0.0 | 0.0 | 0.1 | 0.1 | 0.5 | 0.0 | 0.0 | 0.0 | 0.1 | 0.1 | 0.0 | 0.1 | 0.1 | 0.1 | 0.1 | 0.1 | 0.6 | 1.8 | 0.2 | 0.9 |
| *Arachnia* | 0.6 | 0.8 | 0.4 | 2.3 | 2.8 | 0.0 | 0.3 | 0.1 | 0.4 | 0.3 | 0.0 | 0.1 | 0.0 | 0.2 | 0.2 | 0.0 | 0.2 | 0.1 | 0.1 | 0.3 | 0.0 | 0.4 | 0.2 | 0.3 | 0.4 |
| *Escherichia* | 0.0 | 0.0 | 0.0 | 0.0 | 0.0 | 0.0 | 0.0 | 0.0 | 0.0 | 0.0 | 0.0 | 0.0 | 0.0 | 0.0 | 0.0 | 0.0 | 0.0 | 0.0 | 0.0 | 0.0 | 0.0 | 0.0 | 0.0 | 0.0 | 0.0 |
| *Skermanella* | 0.1 | 0.5 | 0.2 | 5.6 | 2.5 | 0.0 | 0.0 | 0.1 | 0.0 | 0.1 | 0.0 | 0.1 | 0.1 | 0.1 | 0.1 | 0.0 | 0.3 | 0.0 | 0.0 | 0.2 | 0.0 | 0.0 | 0.0 | 0.0 | 0.2 |
| *Microcoleus* | 0.0 | 0.0 | 0.0 | 0.2 | 0.2 | 0.0 | 0.1 | 0.0 | 0.0 | 0.1 | 0.0 | 0.0 | 0.0 | 0.0 | 0.1 | 0.0 | 0.0 | 0.0 | 0.0 | 0.0 | 0.0 | 0.0 | 0.0 | 0.1 | 0.0 |
| *Serratia* | 0.0 | 0.0 | 0.0 | 0.0 | 0.0 | 0.0 | 0.0 | 0.0 | 0.0 | 0.0 | 0.0 | 0.0 | 0.0 | 0.0 | 0.0 | 0.0 | 0.0 | 0.0 | 0.0 | 0.0 | 0.0 | 0.0 | 0.0 | 0.0 | 0.0 |
| *Pseudomonas* | 0.0 | 0.1 | 0.0 | 0.1 | 0.3 | 0.0 | 0.0 | 0.0 | 0.0 | 0.0 | 0.0 | 0.0 | 0.1 | 0.1 | 0.0 | 0.0 | 0.0 | 0.1 | 0.0 | 1.2 | 0.0 | 0.0 | 2.0 | 0.0 | 0.0 |
| *Gemella* | 0.0 | 0.2 | 0.0 | 0.0 | 0.0 | 0.0 | 0.1 | 1.3 | 0.0 | 0.0 | 0.0 | 0.0 | 0.0 | 0.0 | 0.2 | 0.0 | 0.2 | 0.1 | 0.1 | 0.1 | 0.0 | 0.0 | 0.8 | 0.0 | 0.1 |
| *Porphyromonas* | 0.0 | 0.3 | 0.0 | 0.0 | 0.0 | 0.1 | 0.2 | 1.1 | 0.1 | 0.4 | 0.0 | 0.0 | 0.0 | 0.0 | 0.0 | 0.0 | 0.0 | 0.0 | 0.0 | 0.2 | 0.0 | 0.0 | 0.0 | 0.0 | 0.2 |
| *Abiotrophia* | 0.0 | 0.1 | 0.0 | 0.0 | 0.0 | 0.0 | 0.2 | 1.2 | 0.0 | 0.0 | 0.0 | 0.0 | 0.0 | 0.0 | 0.0 | 0.0 | 0.1 | 0.0 | 0.2 | 0.0 | 0.0 | 0.0 | 0.0 | 0.0 | 0.0 |
| *Mycolicibacterium* | 0.0 | 0.2 | 1.0 | 0.9 | 0.1 | 0.2 | 0.6 | 0.9 | 0.5 | 1.4 | 0.4 | 0.4 | 0.5 | 0.8 | 1.2 | 0.1 | 0.1 | 0.2 | 0.1 | 0.5 | 0.0 | 0.1 | 0.3 | 0.2 | 0.1 |
| *Enterococcus* | 0.0 | 0.2 | 0.1 | 0.0 | 0.0 | 0.2 | 0.2 | 1.0 | 0.1 | 0.0 | 0.0 | 0.1 | 0.0 | 0.1 | 0.1 | 0.0 | 0.2 | 0.0 | 0.2 | 0.5 | 0.0 | 0.3 | 0.1 | 0.0 | 0.0 |
| *Actinomyces* | 0.0 | 0.0 | 0.0 | 0.0 | 0.1 | 0.0 | 0.1 | 0.8 | 0.0 | 0.1 | 0.0 | 0.0 | 0.0 | 0.0 | 0.0 | 0.0 | 0.0 | 0.0 | 0.4 | 0.0 | 0.0 | 0.0 | 0.0 | 0.0 | 0.0 |
| *Massilia* | 0.0 | 0.0 | 0.0 | 0.1 | 0.0 | 0.0 | 0.0 | 0.0 | 0.0 | 1.6 | 0.0 | 0.0 | 0.0 | 0.0 | 0.3 | 0.0 | 0.0 | 0.2 | 0.0 | 0.2 | 0.0 | 0.1 | 0.0 | 0.0 | 0.0 |
| *Brevundimonas* | 0.0 | 0.0 | 0.5 | 0.1 | 0.1 | 0.0 | 0.0 | 0.0 | 0.0 | 0.1 | 0.2 | 0.6 | 0.8 | 1.4 | 0.3 | 0.0 | 0.0 | 0.0 | 0.1 | 0.2 | 0.0 | 0.0 | 0.0 | 0.1 | 1.1 |
| *Micrococcus* | 0.0 | 0.1 | 0.0 | 0.0 | 0.0 | 0.0 | 0.0 | 0.1 | 0.0 | 0.0 | 0.0 | 0.0 | 0.0 | 0.0 | 0.0 | 0.0 | 0.0 | 0.0 | 0.0 | 0.1 | 0.0 | 0.0 | 0.6 | 0.0 | 0.0 |
| *Kingella* | 0.0 | 0.0 | 0.0 | 0.4 | 0.2 | 0.0 | 0.0 | 0.1 | 0.0 | 0.0 | 0.0 | 0.6 | 0.1 | 0.7 | 0.7 | 0.0 | 0.1 | 0.1 | 0.0 | 0.0 | 0.0 | 0.5 | 0.0 | 0.8 | 0.2 |
| *Dermacoccus* | 0.1 | 0.1 | 1.0 | 0.1 | 0.1 | 0.0 | 0.0 | 0.1 | 0.0 | 0.1 | 0.0 | 0.3 | 0.3 | 0.3 | 0.1 | 0.0 | 0.0 | 0.0 | 0.2 | 0.2 | 0.0 | 0.0 | 0.0 | 0.0 | 0.0 |
| *Leptotrichia* | 0.0 | 0.0 | 0.0 | 0.0 | 0.1 | 0.0 | 0.0 | 0.8 | 0.0 | 0.1 | 0.0 | 0.0 | 0.0 | 0.0 | 0.0 | 0.0 | 0.2 | 0.0 | 0.5 | 0.0 | 0.0 | 0.1 | 0.0 | 0.0 | 0.0 |
| *Fusobacterium* | 0.0 | 0.1 | 0.3 | 0.0 | 0.0 | 0.0 | 0.1 | 0.7 | 0.0 | 0.4 | 0.0 | 0.0 | 0.0 | 0.0 | 0.0 | 0.0 | 0.1 | 0.0 | 0.0 | 0.1 | 0.0 | 0.0 | 0.0 | 0.0 | 0.0 |
| *Eikenella* | 0.0 | 0.0 | 0.0 | 0.1 | 0.3 | 0.0 | 0.0 | 0.1 | 0.0 | 0.1 | 0.2 | 0.4 | 0.2 | 0.6 | 0.5 | 0.0 | 0.0 | 0.0 | 0.0 | 0.0 | 0.0 | 0.7 | 0.0 | 0.8 | 0.1 |
| *Mycobacterium* | 0.0 | 0.2 | 1.0 | 0.2 | 0.1 | 0.0 | 0.1 | 0.2 | 0.2 | 0.3 | 0.0 | 0.1 | 0.1 | 0.2 | 0.2 | 0.0 | 0.1 | 0.1 | 0.1 | 0.3 | 0.0 | 0.1 | 0.1 | 0.0 | 0.1 |
| *Gordonia* | 0.0 | 0.1 | 0.1 | 0.0 | 0.0 | 0.0 | 0.1 | 0.1 | 0.3 | 0.4 | 0.0 | 0.1 | 0.1 | 0.5 | 0.1 | 0.0 | 0.1 | 0.2 | 0.2 | 0.4 | 0.0 | 0.1 | 0.1 | 0.1 | 0.1 |
| *Tessaracoccus* | 0.0 | 0.1 | 0.1 | 0.2 | 0.1 | 0.0 | 0.2 | 0.1 | 0.3 | 0.1 | 0.0 | 0.1 | 0.0 | 0.2 | 0.2 | 0.0 | 0.1 | 0.1 | 0.2 | 0.2 | 0.0 | 0.3 | 0.1 | 0.1 | 0.2 |
| *Rhodococcus* | 0.0 | 0.1 | 0.1 | 0.1 | 0.0 | 0.0 | 0.2 | 0.3 | 0.5 | 0.3 | 0.0 | 0.1 | 0.1 | 0.4 | 0.1 | 0.0 | 0.1 | 0.1 | 0.1 | 0.4 | 0.0 | 0.1 | 0.1 | 0.1 | 0.3 |
| *Metabacillus* | 0.0 | 0.0 | 0.0 | 0.0 | 0.1 | 0.0 | 0.0 | 0.1 | 0.0 | 0.0 | 0.0 | 0.0 | 0.0 | 0.0 | 0.0 | 0.0 | 0.1 | 0.0 | 0.0 | 0.0 | 0.0 | 0.0 | 0.0 | 0.3 | 0.0 |
| *Prevotellamassilia* | 0.0 | 0.0 | 0.0 | 0.0 | 0.0 | 0.0 | 0.0 | 0.5 | 0.0 | 0.0 | 0.0 | 0.0 | 0.0 | 0.0 | 0.0 | 0.0 | 0.0 | 0.0 | 0.0 | 0.3 | 0.0 | 0.0 | 0.0 | 0.0 | 0.0 |
| *Povalibacter* | 0.0 | 0.1 | 0.2 | 0.0 | 0.0 | 0.0 | 0.0 | 0.0 | 0.0 | 0.0 | 0.0 | 0.0 | 0.0 | 0.0 | 0.1 | 0.0 | 0.0 | 0.0 | 0.1 | 0.0 | 0.0 | 0.0 | 0.0 | 0.1 | 0.0 |
| *Loriellopsis* | 0.0 | 0.0 | 0.0 | 0.1 | 0.1 | 0.0 | 0.0 | 0.0 | 0.0 | 0.0 | 0.0 | 0.0 | 0.0 | 0.0 | 0.0 | 0.0 | 0.0 | 0.0 | 0.0 | 0.0 | 0.0 | 0.0 | 0.0 | 0.1 | 0.0 |
| *Microbacterium* | 0.0 | 0.1 | 0.0 | 1.2 | 0.0 | 0.0 | 0.1 | 0.1 | 0.1 | 0.0 | 0.0 | 0.1 | 0.0 | 0.0 | 0.1 | 0.0 | 0.0 | 0.2 | 0.0 | 0.0 | 0.0 | 0.0 | 0.0 | 0.0 | 0.1 |
| *Methylorubrum* | 0.0 | 0.0 | 0.0 | 0.0 | 0.0 | 0.0 | 0.0 | 0.0 | 0.0 | 0.0 | 0.0 | 0.0 | 0.0 | 0.0 | 0.0 | 0.0 | 0.0 | 0.0 | 0.0 | 0.0 | 0.0 | 0.0 | 0.0 | 0.0 | 0.0 |
| *Chryseobacterium* | 0.0 | 0.0 | 0.0 | 0.0 | 0.0 | 0.0 | 0.0 | 0.4 | 0.1 | 0.2 | 0.0 | 0.0 | 0.1 | 0.0 | 0.0 | 0.2 | 0.5 | 0.9 | 0.0 | 0.0 | 0.0 | 0.0 | 0.0 | 0.0 | 0.0 |
| *Brevibacterium* | 0.0 | 0.0 | 0.0 | 0.1 | 0.2 | 0.0 | 0.1 | 0.0 | 0.2 | 0.3 | 0.0 | 0.0 | 0.0 | 0.0 | 0.0 | 0.0 | 0.0 | 0.0 | 0.2 | 0.0 | 0.0 | 0.0 | 0.2 | 0.0 | 0.0 |
| *Nocardioides* | 0.0 | 0.1 | 0.0 | 0.1 | 0.1 | 0.0 | 0.1 | 0.0 | 0.1 | 0.1 | 0.0 | 0.1 | 0.0 | 0.1 | 0.1 | 0.0 | 0.1 | 0.0 | 0.1 | 0.2 | 0.0 | 0.1 | 0.1 | 0.1 | 0.1 |
| *Mammaliicoccus* | 0.0 | 0.1 | 0.1 | 0.0 | 0.0 | 0.0 | 0.2 | 0.4 | 0.1 | 0.1 | 0.7 | 0.4 | 0.3 | 0.1 | 0.1 | 0.1 | 0.2 | 0.1 | 0.1 | 0.1 | 0.0 | 0.1 | 0.1 | 0.1 | 0.1 |
| *Lactobacillus* | 0.0 | 0.0 | 0.0 | 0.0 | 0.0 | 0.0 | 0.0 | 0.0 | 0.0 | 0.0 | 0.0 | 0.0 | 0.0 | 0.0 | 0.0 | 0.0 | 0.0 | 0.0 | 0.0 | 0.1 | 0.0 | 0.0 | 0.0 | 0.0 | 0.0 |
| *Steroidobacter* | 0.0 | 0.2 | 0.2 | 0.0 | 0.0 | 0.0 | 0.0 | 0.0 | 0.0 | 0.0 | 0.0 | 0.0 | 0.1 | 0.0 | 0.0 | 0.0 | 0.0 | 0.0 | 0.1 | 0.0 | 0.0 | 0.0 | 0.0 | 0.1 | 0.0 |
| *Rahnella* | 0.0 | 0.0 | 0.0 | 0.0 | 0.0 | 0.0 | 0.0 | 0.0 | 0.0 | 0.0 | 0.0 | 0.0 | 0.0 | 0.0 | 0.0 | 0.0 | 0.0 | 0.0 | 0.0 | 0.0 | 0.0 | 0.0 | 0.0 | 0.0 | 0.0 |
| *Macrococcus* | 0.0 | 0.1 | 0.1 | 0.0 | 0.0 | 0.0 | 0.3 | 0.3 | 0.1 | 0.1 | 0.0 | 0.2 | 0.2 | 0.0 | 0.0 | 0.0 | 0.2 | 0.1 | 0.1 | 0.1 | 0.0 | 0.1 | 0.1 | 0.1 | 0.1 |
| *Pseudonocardia* | 0.0 | 0.1 | 0.1 | 0.7 | 0.1 | 0.0 | 0.1 | 0.1 | 0.1 | 0.1 | 0.0 | 0.0 | 0.0 | 0.1 | 0.1 | 0.0 | 0.1 | 0.1 | 0.1 | 0.2 | 0.0 | 0.1 | 0.1 | 0.1 | 0.1 |
| *Alysiella* | 0.0 | 0.0 | 0.0 | 0.1 | 0.4 | 0.0 | 0.0 | 0.0 | 0.0 | 0.0 | 0.0 | 0.1 | 0.0 | 0.2 | 0.1 | 0.0 | 0.0 | 0.0 | 0.0 | 0.0 | 0.0 | 0.1 | 0.0 | 0.2 | 0.0 |
| *Dialister* | 0.0 | 0.0 | 0.0 | 0.0 | 0.0 | 0.0 | 0.0 | 0.1 | 0.0 | 0.2 | 0.0 | 0.0 | 0.0 | 0.1 | 0.0 | 0.0 | 0.0 | 0.0 | 0.0 | 0.0 | 0.0 | 0.0 | 0.0 | 0.0 | 0.1 |
| *Psychrobacter* | 0.0 | 0.0 | 0.4 | 0.0 | 0.0 | 0.0 | 0.0 | 0.0 | 0.0 | 0.3 | 0.4 | 0.3 | 0.6 | 0.1 | 0.1 | 0.2 | 0.1 | 0.3 | 0.2 | 0.1 | 0.0 | 0.0 | 0.0 | 0.1 | 0.3 |
| *Pantoea* | 0.1 | 0.4 | 0.0 | 0.0 | 0.0 | 0.0 | 0.0 | 0.0 | 0.0 | 0.0 | 0.0 | 0.0 | 0.0 | 0.0 | 0.0 | 0.0 | 0.0 | 0.0 | 0.0 | 0.0 | 0.0 | 0.0 | 0.0 | 0.0 | 0.0 |
| *Cupriavidus* | 0.0 | 0.0 | 0.0 | 0.0 | 0.0 | 0.0 | 0.0 | 0.0 | 0.0 | 0.0 | 0.0 | 0.0 | 0.0 | 0.0 | 0.1 | 0.0 | 0.0 | 0.0 | 0.1 | 0.0 | 0.0 | 0.0 | 0.0 | 0.0 | 0.0 |
| *Simonsiella* | 0.0 | 0.0 | 0.0 | 0.0 | 0.1 | 0.0 | 0.0 | 0.0 | 0.0 | 0.0 | 0.1 | 0.2 | 0.2 | 0.3 | 0.3 | 0.0 | 0.0 | 0.0 | 0.0 | 0.0 | 0.0 | 0.5 | 0.0 | 0.5 | 0.2 |
| *Lautropia* | 0.0 | 0.0 | 0.0 | 0.0 | 0.0 | 0.0 | 0.0 | 0.5 | 0.0 | 0.0 | 0.0 | 0.0 | 0.0 | 0.0 | 0.0 | 0.0 | 0.0 | 0.0 | 0.0 | 0.3 | 0.0 | 0.1 | 0.0 | 0.0 | 0.0 |
| *Erwinia* | 0.0 | 0.3 | 0.0 | 0.0 | 0.0 | 0.0 | 0.0 | 0.0 | 0.0 | 0.0 | 0.0 | 0.0 | 0.0 | 0.0 | 0.0 | 0.0 | 0.0 | 0.0 | 0.0 | 0.0 | 0.0 | 0.0 | 0.0 | 0.0 | 0.0 |
| *Nigerium* | 0.0 | 0.1 | 0.0 | 0.0 | 0.0 | 0.0 | 0.1 | 0.0 | 0.2 | 0.1 | 0.0 | 0.0 | 0.0 | 0.0 | 0.1 | 0.0 | 0.1 | 0.0 | 0.1 | 0.1 | 0.0 | 0.1 | 0.0 | 0.1 | 0.1 |
| *Aliterella* | 0.0 | 0.0 | 0.0 | 0.2 | 0.3 | 0.0 | 0.0 | 0.0 | 0.0 | 0.0 | 0.0 | 0.0 | 0.0 | 0.0 | 0.0 | 0.0 | 0.0 | 0.0 | 0.0 | 0.0 | 0.0 | 0.0 | 0.0 | 0.0 | 0.0 |
| *Aggregatibacter* | 0.0 | 0.0 | 0.0 | 0.0 | 0.0 | 0.0 | 0.0 | 0.4 | 0.0 | 0.0 | 0.0 | 0.0 | 0.0 | 0.0 | 0.0 | 0.0 | 0.0 | 0.0 | 0.2 | 0.3 | 0.0 | 0.0 | 0.0 | 0.0 | 0.0 |
| *Dietzia* | 0.0 | 0.1 | 0.1 | 0.0 | 0.0 | 0.0 | 0.1 | 0.1 | 0.2 | 0.1 | 0.0 | 0.0 | 0.0 | 0.0 | 0.0 | 0.0 | 0.1 | 0.1 | 0.1 | 0.6 | 0.0 | 0.0 | 0.1 | 0.1 | 0.1 |
| *Bifidobacterium* | 0.0 | 0.0 | 0.0 | 0.0 | 0.0 | 0.0 | 0.0 | 0.0 | 0.0 | 0.0 | 0.0 | 0.0 | 0.0 | 0.0 | 0.0 | 0.0 | 0.0 | 0.0 | 0.0 | 0.0 | 0.0 | 0.0 | 0.0 | 0.0 | 0.0 |
| *Vitreoscilla* | 0.0 | 0.0 | 0.0 | 0.1 | 0.0 | 0.0 | 0.0 | 0.0 | 0.0 | 0.0 | 0.0 | 0.2 | 0.0 | 0.3 | 0.3 | 0.0 | 0.0 | 0.0 | 0.0 | 0.0 | 0.0 | 0.4 | 0.0 | 0.5 | 0.1 |
| *Tsukamurella* | 0.0 | 0.1 | 0.1 | 0.0 | 0.0 | 0.0 | 0.1 | 0.1 | 0.2 | 0.1 | 0.0 | 0.0 | 0.0 | 0.1 | 0.0 | 0.0 | 0.1 | 0.1 | 0.1 | 0.7 | 0.0 | 0.0 | 0.1 | 0.1 | 0.1 |
| *Schaalia* | 0.0 | 0.0 | 0.0 | 0.0 | 0.0 | 0.0 | 0.0 | 0.1 | 0.0 | 0.0 | 0.0 | 0.0 | 0.0 | 0.0 | 0.0 | 0.0 | 0.0 | 0.0 | 0.0 | 0.0 | 0.0 | 0.1 | 0.0 | 0.0 | 0.0 |
| *Gloeocapsopsis* | 0.0 | 0.0 | 0.2 | 0.1 | 0.1 | 0.0 | 0.0 | 0.0 | 0.0 | 0.0 | 0.0 | 0.0 | 0.0 | 0.0 | 0.0 | 0.0 | 0.0 | 0.0 | 0.0 | 0.0 | 0.0 | 0.0 | 0.0 | 0.1 | 0.0 |
| *Klebsiella* | 0.0 | 0.0 | 0.4 | 0.0 | 0.0 | 0.0 | 0.0 | 0.0 | 0.0 | 0.0 | 0.0 | 0.0 | 0.0 | 0.0 | 0.0 | 0.0 | 0.0 | 0.0 | 0.0 | 0.0 | 0.0 | 0.0 | 0.0 | 0.0 | 0.0 |
| *Rhizobium* | 0.0 | 0.1 | 0.3 | 0.1 | 0.1 | 0.0 | 0.0 | 0.0 | 0.0 | 0.1 | 0.0 | 0.2 | 0.4 | 0.2 | 0.0 | 0.0 | 0.0 | 0.0 | 0.0 | 0.1 | 0.0 | 0.3 | 0.1 | 0.0 | 0.4 |
| *Nocardia* | 0.0 | 0.0 | 0.1 | 0.1 | 0.0 | 0.0 | 0.1 | 0.1 | 0.1 | 0.1 | 0.0 | 0.1 | 0.0 | 0.2 | 0.1 | 0.0 | 0.0 | 0.1 | 0.1 | 0.3 | 0.0 | 0.0 | 0.0 | 0.0 | 0.0 |
| *Brachybacterium* | 0.0 | 0.0 | 0.4 | 0.1 | 0.0 | 0.0 | 0.0 | 0.0 | 0.0 | 0.0 | 0.0 | 0.0 | 0.0 | 0.0 | 0.0 | 0.0 | 0.0 | 0.0 | 0.0 | 0.0 | 0.0 | 0.1 | 0.0 | 0.0 | 0.0 |
| *Janibacter* | 0.0 | 0.0 | 0.0 | 0.1 | 0.0 | 0.1 | 0.3 | 0.4 | 0.3 | 0.6 | 0.0 | 0.0 | 0.0 | 0.0 | 0.0 | 0.0 | 0.0 | 0.0 | 0.0 | 0.0 | 0.0 | 0.0 | 0.0 | 0.0 | 0.0 |
| *Lysobacter* | 0.0 | 0.1 | 0.0 | 2.1 | 0.0 | 0.0 | 0.0 | 0.0 | 0.0 | 0.0 | 0.0 | 0.1 | 0.3 | 0.0 | 0.0 | 0.0 | 0.0 | 0.0 | 0.0 | 0.0 | 0.0 | 0.1 | 0.0 | 0.0 | 0.0 |
| *Facklamia* | 0.0 | 0.0 | 0.3 | 0.0 | 0.0 | 0.0 | 0.0 | 0.1 | 0.0 | 0.0 | 0.0 | 0.0 | 0.0 | 0.0 | 0.0 | 0.0 | 0.0 | 0.0 | 0.0 | 0.0 | 0.0 | 0.0 | 0.0 | 0.0 | 0.0 |
| *Sphingopyxis* | 0.0 | 0.0 | 0.0 | 0.2 | 0.0 | 0.0 | 0.0 | 0.0 | 0.0 | 0.0 | 0.0 | 0.0 | 0.0 | 0.0 | 0.0 | 0.0 | 0.0 | 0.0 | 0.0 | 0.0 | 0.0 | 0.0 | 0.0 | 0.0 | 0.0 |
| *Flavobacterium* | 0.0 | 0.0 | 0.5 | 0.1 | 0.0 | 0.0 | 0.0 | 0.0 | 0.0 | 0.0 | 0.0 | 0.0 | 0.0 | 0.0 | 0.0 | 0.0 | 0.0 | 0.0 | 0.0 | 0.0 | 0.0 | 0.0 | 0.0 | 0.0 | 0.0 |
| *Stenotrophomonas* | 0.0 | 0.3 | 0.0 | 0.0 | 0.0 | 0.0 | 0.0 | 0.0 | 0.0 | 0.0 | 0.0 | 0.1 | 0.0 | 0.0 | 0.0 | 0.0 | 0.3 | 0.1 | 0.0 | 0.0 | 0.0 | 0.0 | 0.0 | 0.0 | 0.0 |
| *Afipia* | 0.0 | 0.2 | 0.1 | 0.1 | 0.0 | 0.0 | 0.0 | 0.1 | 0.0 | 0.0 | 0.0 | 0.0 | 0.0 | 0.0 | 0.0 | 0.0 | 0.0 | 0.0 | 0.0 | 0.0 | 0.0 | 0.0 | 0.0 | 0.0 | 0.0 |
| *Pseudoglutamicibacter* | 0.0 | 0.2 | 0.2 | 0.0 | 0.1 | 0.0 | 0.0 | 0.0 | 0.0 | 0.1 | 0.0 | 0.0 | 0.0 | 0.0 | 0.0 | 0.0 | 0.0 | 0.0 | 0.0 | 0.0 | 0.0 | 0.0 | 0.0 | 0.0 | 0.0 |
| *Hydrobacter* | 0.0 | 0.0 | 0.0 | 0.0 | 0.0 | 0.0 | 0.0 | 0.0 | 0.0 | 0.0 | 0.0 | 0.0 | 0.0 | 0.0 | 0.0 | 0.0 | 0.0 | 0.0 | 0.0 | 0.0 | 0.0 | 0.1 | 0.2 | 0.0 | 0.0 |
| *Dermabacter* | 0.0 | 0.0 | 0.0 | 0.0 | 0.0 | 0.0 | 0.0 | 0.0 | 0.0 | 0.1 | 0.0 | 0.0 | 0.0 | 0.0 | 0.0 | 0.0 | 0.2 | 0.5 | 0.0 | 0.0 | 0.0 | 0.3 | 0.0 | 0.0 | 0.0 |
| *Pectobacterium* | 0.0 | 0.0 | 0.2 | 0.0 | 0.0 | 0.0 | 0.0 | 0.0 | 0.0 | 0.0 | 0.0 | 0.0 | 0.0 | 0.0 | 0.0 | 0.0 | 0.0 | 0.0 | 0.0 | 0.0 | 0.0 | 0.0 | 0.0 | 0.0 | 0.0 |
| *Stanieria* | 0.0 | 0.0 | 0.0 | 0.0 | 0.0 | 0.0 | 0.0 | 0.0 | 0.0 | 0.0 | 0.0 | 0.0 | 0.0 | 0.0 | 0.0 | 0.0 | 0.0 | 0.0 | 0.0 | 0.0 | 0.0 | 0.0 | 0.0 | 0.0 | 0.0 |
| *Capnocytophaga* | 0.0 | 0.0 | 0.0 | 0.0 | 0.0 | 0.0 | 0.0 | 0.2 | 0.0 | 0.2 | 0.0 | 0.0 | 0.0 | 0.0 | 0.0 | 0.0 | 0.0 | 0.0 | 0.2 | 0.0 | 0.0 | 0.0 | 0.0 | 0.0 | 0.1 |
| Other genera | 8.2 | 6.7 | 8.2 | 24.0 | 5.7 | 4.4 | 2.8 | 8.9 | 4.0 | 4.9 | 5.1 | 4.8 | 14.5 | 6.5 | 5.5 | 3.9 | 3.3 | 4.8 | 5.7 | 4.5 | 8.8 | 7.1 | 3.5 | 3.9 | 2.4 |

| | Subject_11 | | | | | Subject_12 | | | | | Subject_13 | | | | | Subject_14 | | | | | Subject_15 | | | | |
|---|---|---|---|---|---|---|---|---|---|---|---|---|---|---|---|---|---|---|---|---|---|---|---|---|---|
| Taxa | Baseline | Week 2 | Week 4 | Week 8 | Week 12 | Baseline | Week 2 | Week 4 | Week 8 | Week 12 | Baseline | Week 2 | Week 4 | Week 8 | Week 12 | Baseline | Week 2 | Week 4 | Week 8 | Week 12 | Baseline | Week 2 | Week 4 | Week 8 | Week 12 |
| *Cutibacterium* | 22.3 | 23.0 | 32.8 | 50.4 | 41.6 | 39.1 | 30.5 | 31.7 | 47.7 | 41.7 | 7.4 | 7.6 | 37.2 | 30.2 | 41.2 | 17.4 | 18.1 | 14.4 | 54.7 | 49.2 | 38.1 | 35.2 | 38.5 | 53.8 | 57.0 |
| *Staphylococcus* | 44.1 | 31.7 | 17.4 | 11.2 | 10.7 | 16.5 | 11.9 | 11.2 | 2.0 | 2.1 | 67.2 | 48.9 | 16.2 | 5.1 | 8.7 | 44.1 | 35.0 | 28.8 | 0.6 | 1.5 | 51.1 | 42.7 | 19.8 | 17.1 | 14.1 |
| *Corynebacterium* | 11.2 | 15.3 | 16.2 | 13.8 | 14.1 | 5.8 | 5.8 | 5.0 | 5.1 | 4.5 | 3.9 | 6.6 | 5.8 | 15.8 | 11.3 | 0.8 | 0.6 | 1.4 | 3.3 | 0.2 | 0.2 | 0.3 | 4.3 | 0.6 | 0.8 |
| *Streptococcus* | 15.3 | 15.4 | 13.3 | 5.4 | 6.0 | 19.0 | 19.4 | 18.7 | 1.8 | 1.7 | 1.9 | 3.5 | 4.5 | 11.9 | 18.7 | 9.8 | 8.0 | 9.6 | 1.1 | 0.2 | 5.0 | 6.3 | 5.4 | 8.9 | 11.4 |
| *Snodgrassella* | 0.1 | 0.0 | 0.3 | 0.1 | 1.1 | 0.0 | 0.0 | 0.0 | 0.0 | 0.0 | 0.0 | 0.1 | 0.0 | 1.0 | 0.0 | 15.8 | 16.4 | 18.2 | 16.8 | 13.7 | 0.0 | 0.0 | 0.0 | 0.0 | 0.1 |
| *Moraxella* | 0.2 | 0.2 | 0.0 | 1.6 | 4.5 | 8.2 | 2.7 | 8.1 | 7.2 | 10.3 | 15.1 | 19.4 | 7.0 | 1.4 | 0.1 | 0.3 | 0.4 | 2.9 | 0.5 | 0.0 | 2.1 | 2.5 | 11.3 | 0.4 | 0.7 |
| *Finegoldia* | 0.7 | 1.3 | 1.5 | 2.8 | 0.5 | 0.1 | 0.3 | 0.5 | 0.0 | 0.4 | 0.1 | 0.1 | 0.0 | 0.8 | 0.0 | 0.1 | 0.1 | 0.7 | 0.2 | 0.0 | 0.0 | 0.0 | 0.0 | 0.0 | 0.0 |
| *Acinetobacter* | 0.0 | 0.0 | 0.1 | 0.1 | 0.0 | 0.4 | 0.9 | 0.6 | 0.2 | 0.1 | 1.7 | 0.8 | 6.6 | 4.4 | 1.2 | 2.5 | 2.6 | 1.1 | 1.5 | 0.0 | 0.0 | 0.0 | 0.0 | 0.0 | 0.5 |
| *Deinococcus* | 0.0 | 0.0 | 0.4 | 0.4 | 0.7 | 0.0 | 0.1 | 0.2 | 0.2 | 0.2 | 0.0 | 0.0 | 0.0 | 0.4 | 0.0 | 0.0 | 0.0 | 0.0 | 0.0 | 0.0 | 0.0 | 0.0 | 0.0 | 0.1 | 0.2 |
| *Roseomonas* | 0.0 | 0.0 | 0.0 | 0.1 | 0.3 | 0.0 | 0.1 | 0.5 | 2.4 | 2.6 | 0.0 | 0.2 | 0.4 | 1.4 | 3.8 | 0.0 | 0.0 | 0.0 | 0.0 | 0.0 | 0.0 | 0.0 | 0.0 | 0.0 | 0.0 |
| *Neisseria* | 0.1 | 0.5 | 0.1 | 0.0 | 1.5 | 1.9 | 3.2 | 0.7 | 0.1 | 0.4 | 0.0 | 0.0 | 0.9 | 2.0 | 0.0 | 0.2 | 2.5 | 0.3 | 2.0 | 0.1 | 0.0 | 0.4 | 0.0 | 1.3 | 0.5 |
| *Rothia* | 0.2 | 0.6 | 0.8 | 0.0 | 0.7 | 0.2 | 1.3 | 0.4 | 0.3 | 0.3 | 0.0 | 0.0 | 0.8 | 0.0 | 0.0 | 0.1 | 0.8 | 0.8 | 0.3 | 0.0 | 0.1 | 0.2 | 1.1 | 0.8 | 1.0 |
| *Prevotella* | 0.2 | 0.2 | 1.3 | 1.2 | 0.6 | 0.2 | 0.9 | 0.5 | 0.0 | 0.0 | 0.0 | 0.0 | 2.3 | 0.0 | 0.0 | 0.0 | 0.0 | 0.9 | 0.1 | 0.0 | 0.0 | 0.2 | 0.0 | 1.3 | 1.2 |
| *Anaerococcus* | 0.4 | 1.3 | 3.3 | 0.5 | 0.7 | 0.0 | 0.1 | 0.3 | 0.1 | 0.1 | 0.0 | 0.2 | 0.0 | 0.4 | 0.0 | 0.0 | 0.0 | 0.8 | 0.2 | 0.0 | 0.0 | 0.1 | 1.4 | 0.1 | 0.0 |
| *Kocuria* | 0.1 | 0.1 | 0.6 | 0.3 | 0.6 | 0.0 | 0.4 | 0.4 | 1.4 | 1.3 | 0.0 | 0.0 | 0.1 | 0.7 | 0.0 | 0.0 | 0.1 | 1.4 | 0.4 | 0.0 | 0.0 | 0.1 | 0.1 | 0.1 | 0.1 |
| *Haemophilus* | 0.0 | 0.0 | 0.0 | 0.3 | 0.6 | 0.4 | 1.3 | 0.7 | 0.0 | 0.0 | 0.0 | 0.1 | 2.0 | 2.5 | 1.1 | 0.0 | 0.4 | 0.3 | 0.0 | 0.0 | 0.2 | 0.8 | 1.0 | 2.2 | 1.3 |
| *Paracoccus* | 0.1 | 0.2 | 0.2 | 0.1 | 0.1 | 0.2 | 0.3 | 1.3 | 6.3 | 9.7 | 0.2 | 3.6 | 0.1 | 1.3 | 1.8 | 0.0 | 0.0 | 0.1 | 0.0 | 0.0 | 0.0 | 0.0 | 0.0 | 0.1 | 0.0 |
| *Lawsonella* | 0.2 | 0.4 | 0.8 | 0.2 | 0.3 | 0.2 | 0.7 | 1.5 | 0.1 | 0.1 | 0.2 | 0.0 | 1.2 | 0.3 | 0.0 | 0.0 | 0.2 | 0.2 | 0.1 | 0.0 | 0.0 | 0.0 | 0.0 | 0.0 | 0.0 |
| *Bacillus* | 0.2 | 1.0 | 0.4 | 0.2 | 0.4 | 0.3 | 1.8 | 1.2 | 0.1 | 0.4 | 0.0 | 0.1 | 1.5 | 1.0 | 0.2 | 0.0 | 1.2 | 1.1 | 0.0 | 0.0 | 0.0 | 0.3 | 0.6 | 0.1 | 0.1 |
| *Methylobacterium* | 0.0 | 0.0 | 0.0 | 0.3 | 0.8 | 0.0 | 0.1 | 0.2 | 0.6 | 1.2 | 0.0 | 0.1 | 0.0 | 0.1 | 0.0 | 0.0 | 0.0 | 0.0 | 0.0 | 0.0 | 0.0 | 0.0 | 0.0 | 0.0 | 0.0 |
| *Veillonella* | 0.0 | 0.0 | 0.0 | 0.1 | 0.0 | 0.0 | 0.6 | 0.8 | 0.0 | 0.0 | 0.0 | 0.0 | 1.2 | 0.0 | 0.0 | 0.0 | 0.1 | 0.5 | 0.0 | 0.0 | 0.0 | 0.6 | 0.0 | 0.8 | 0.2 |
| *Granulicatella* | 0.0 | 0.6 | 0.2 | 1.0 | 0.8 | 0.0 | 2.0 | 0.8 | 0.0 | 0.2 | 0.0 | 0.0 | 1.4 | 0.0 | 0.0 | 0.0 | 0.3 | 0.0 | 0.4 | 0.0 | 0.0 | 0.8 | 0.0 | 2.4 | 0.0 |
| *Sphingomonas* | 0.0 | 0.0 | 0.0 | 0.0 | 0.2 | 0.0 | 0.3 | 0.1 | 1.1 | 2.0 | 0.0 | 1.1 | 0.3 | 0.0 | 0.0 | 0.0 | 0.2 | 0.5 | 0.0 | 0.0 | 0.0 | 0.0 | 1.5 | 0.1 | 0.0 |
| *Williamsia* | 0.0 | 0.0 | 0.0 | 0.0 | 0.0 | 0.8 | 1.0 | 1.2 | 8.4 | 4.9 | 0.0 | 0.0 | 0.0 | 0.7 | 0.0 | 0.0 | 0.0 | 1.0 | 0.3 | 0.0 | 0.0 | 0.0 | 0.0 | 0.0 | 0.0 |
| *Peptoniphilus* | 0.2 | 0.8 | 0.2 | 0.7 | 2.5 | 0.2 | 0.2 | 0.6 | 0.3 | 0.2 | 0.0 | 0.0 | 0.6 | 0.0 | 0.4 | 0.0 | 0.0 | 0.5 | 0.1 | 0.0 | 0.0 | 0.0 | 0.0 | 0.0 | 0.0 |
| *Arachnia* | 0.0 | 0.2 | 0.5 | 0.6 | 0.4 | 0.0 | 0.4 | 0.4 | 0.3 | 0.3 | 0.0 | 0.0 | 0.3 | 0.2 | 0.3 | 0.0 | 0.1 | 0.1 | 0.3 | 0.0 | 0.0 | 0.3 | 0.6 | 0.3 | 0.3 |
| *Escherichia* | 0.0 | 0.0 | 0.0 | 0.0 | 0.0 | 0.0 | 0.0 | 0.0 | 0.0 | 0.0 | 0.0 | 0.0 | 0.0 | 0.0 | 0.0 | 0.0 | 0.0 | 0.0 | 0.0 | 0.0 | 0.0 | 0.0 | 0.0 | 0.0 | 0.0 |
| *Skermanella* | 0.0 | 0.0 | 0.0 | 0.2 | 0.9 | 0.0 | 0.0 | 0.0 | 0.0 | 0.2 | 0.0 | 0.3 | 0.0 | 0.0 | 1.1 | 0.0 | 0.0 | 0.0 | 0.0 | 0.0 | 0.0 | 0.0 | 0.0 | 0.0 | 0.0 |
| *Microcoleus* | 0.0 | 0.1 | 0.0 | 0.0 | 0.4 | 0.0 | 0.0 | 0.0 | 0.0 | 0.0 | 0.0 | 0.0 | 0.0 | 0.2 | 0.0 | 0.0 | 0.3 | 0.2 | 0.9 | 0.0 | 0.0 | 0.8 | 0.6 | 0.1 | 0.0 |
| *Serratia* | 0.0 | 0.0 | 0.0 | 0.3 | 0.0 | 0.0 | 0.0 | 0.0 | 0.0 | 0.0 | 0.0 | 0.0 | 0.0 | 0.0 | 0.0 | 0.0 | 0.6 | 0.9 | 0.0 | 0.2 | 0.0 | 0.0 | 0.0 | 0.0 | 0.0 |
| *Pseudomonas* | 0.0 | 0.0 | 0.0 | 0.0 | 0.0 | 0.0 | 0.1 | 0.0 | 0.0 | 0.0 | 0.0 | 0.2 | 0.0 | 0.0 | 0.0 | 0.0 | 0.5 | 1.1 | 0.3 | 0.0 | 0.0 | 0.0 | 0.0 | 0.1 | 0.0 |
| *Gemella* | 0.0 | 0.4 | 0.2 | 0.0 | 0.0 | 0.0 | 0.5 | 0.6 | 0.0 | 0.1 | 0.0 | 0.0 | 0.7 | 0.0 | 0.0 | 0.0 | 0.1 | 0.0 | 0.0 | 0.0 | 0.0 | 0.3 | 0.7 | 0.7 | 0.6 |
| *Porphyromonas* | 0.0 | 0.1 | 0.0 | 0.0 | 0.0 | 0.0 | 0.0 | 0.0 | 0.0 | 0.0 | 0.0 | 0.0 | 0.6 | 0.0 | 0.0 | 0.0 | 0.0 | 0.0 | 0.0 | 0.0 | 0.0 | 0.0 | 0.9 | 0.7 | 0.0 |
| *Abiotrophia* | 0.0 | 0.1 | 0.0 | 0.2 | 0.0 | 0.0 | 0.4 | 0.3 | 0.2 | 0.0 | 0.0 | 0.0 | 0.0 | 0.0 | 0.0 | 0.0 | 0.0 | 0.1 | 0.0 | 0.0 | 0.0 | 0.3 | 0.0 | 0.5 | 0.0 |
| *Mycolicibacterium* | 0.0 | 0.1 | 0.1 | 0.7 | 0.1 | 0.0 | 0.1 | 0.2 | 0.2 | 2.3 | 0.0 | 0.0 | 0.2 | 0.1 | 0.0 | 0.0 | 0.0 | 0.2 | 0.0 | 0.0 | 0.0 | 0.0 | 0.4 | 0.0 | 0.0 |
| *Enterococcus* | 0.0 | 0.7 | 0.2 | 0.2 | 0.2 | 0.0 | 0.8 | 0.4 | 0.2 | 0.0 | 0.0 | 0.0 | 0.2 | 0.1 | 0.1 | 0.0 | 0.4 | 0.5 | 0.2 | 0.0 | 0.0 | 0.2 | 0.1 | 0.6 | 0.0 |
| *Actinomyces* | 0.0 | 0.0 | 0.2 | 0.1 | 0.2 | 0.0 | 0.1 | 0.0 | 0.1 | 0.1 | 0.0 | 0.0 | 0.1 | 1.4 | 0.1 | 0.0 | 0.0 | 0.0 | 0.0 | 0.0 | 0.0 | 0.0 | 0.0 | 0.5 | 0.0 |
| *Massilia* | 0.0 | 0.0 | 0.0 | 0.0 | 0.0 | 0.0 | 0.0 | 0.0 | 0.0 | 0.0 | 0.0 | 0.0 | 0.0 | 0.0 | 0.0 | 0.0 | 0.0 | 0.0 | 0.0 | 0.0 | 0.0 | 0.0 | 0.0 | 0.0 | 0.0 |
| *Brevundimonas* | 0.0 | 0.0 | 0.0 | 0.2 | 0.0 | 0.0 | 0.1 | 0.0 | 0.0 | 0.4 | 0.0 | 2.1 | 0.0 | 0.0 | 0.7 | 0.0 | 0.0 | 0.0 | 0.0 | 0.0 | 0.0 | 0.0 | 0.8 | 0.0 | 0.0 |
| *Micrococcus* | 0.0 | 0.2 | 0.0 | 0.4 | 0.2 | 0.0 | 0.1 | 0.1 | 0.3 | 0.1 | 0.0 | 0.3 | 0.2 | 0.4 | 1.6 | 0.0 | 0.0 | 0.0 | 0.0 | 0.0 | 0.0 | 0.1 | 0.6 | 0.0 | 0.1 |
| *Kingella* | 0.0 | 0.0 | 0.0 | 0.0 | 0.1 | 0.0 | 0.0 | 0.0 | 0.0 | 0.0 | 0.0 | 0.0 | 0.2 | 1.3 | 0.0 | 0.1 | 0.5 | 0.1 | 1.1 | 0.0 | 0.0 | 0.0 | 0.0 | 0.0 | 0.1 |
| *Dermacoccus* | 0.0 | 0.0 | 0.0 | 0.2 | 0.1 | 0.0 | 0.2 | 1.0 | 2.4 | 1.9 | 0.0 | 0.1 | 0.0 | 0.3 | 0.0 | 0.0 | 0.0 | 0.5 | 0.0 | 0.0 | 0.0 | 0.0 | 0.0 | 0.0 | 0.0 |
| *Leptotrichia* | 0.0 | 0.0 | 0.0 | 0.0 | 0.0 | 0.0 | 1.4 | 0.2 | 0.0 | 0.0 | 0.0 | 0.0 | 0.9 | 0.0 | 0.0 | 0.0 | 0.0 | 0.0 | 0.0 | 0.0 | 0.0 | 0.0 | 0.0 | 0.4 | 1.0 |
| *Fusobacterium* | 0.0 | 0.0 | 0.0 | 0.1 | 0.0 | 0.0 | 1.5 | 0.2 | 0.0 | 0.0 | 0.0 | 0.0 | 0.0 | 0.0 | 0.0 | 0.0 | 0.0 | 0.1 | 0.0 | 0.0 | 0.0 | 0.0 | 0.0 | 0.0 | 0.0 |
| *Eikenella* | 0.0 | 0.0 | 0.0 | 0.0 | 0.1 | 0.0 | 0.0 | 0.1 | 0.0 | 0.0 | 0.0 | 0.0 | 0.1 | 0.1 | 0.0 | 0.0 | 0.7 | 0.2 | 1.9 | 0.0 | 0.0 | 0.0 | 0.0 | 0.0 | 0.0 |
| *Mycobacterium* | 0.0 | 0.0 | 0.1 | 0.2 | 0.1 | 0.0 | 0.1 | 0.1 | 0.1 | 0.8 | 0.0 | 0.0 | 0.1 | 0.0 | 0.1 | 0.2 | 0.0 | 0.1 | 0.1 | 0.0 | 0.0 | 0.0 | 0.1 | 0.0 | 0.0 |
| *Gordonia* | 0.0 | 0.1 | 0.1 | 0.1 | 0.3 | 0.0 | 0.2 | 0.2 | 1.1 | 0.9 | 0.0 | 0.0 | 0.1 | 0.2 | 0.0 | 0.0 | 0.0 | 0.2 | 0.0 | 0.0 | 0.0 | 0.0 | 0.0 | 0.0 | 0.0 |
| *Tessaracoccus* | 0.0 | 0.2 | 0.2 | 0.2 | 0.2 | 0.0 | 0.1 | 0.2 | 0.4 | 0.3 | 0.0 | 0.0 | 0.1 | 0.1 | 0.1 | 0.0 | 0.1 | 0.0 | 0.1 | 0.0 | 0.1 | 0.2 | 0.1 | 0.1 | 0.2 |
| *Rhodococcus* | 0.0 | 0.1 | 0.1 | 0.2 | 0.1 | 0.0 | 0.1 | 0.2 | 0.6 | 0.5 | 0.0 | 0.0 | 0.1 | 0.1 | 0.0 | 0.0 | 0.0 | 0.1 | 0.0 | 0.0 | 0.0 | 0.0 | 0.0 | 0.0 | 0.0 |
| *Metabacillus* | 0.0 | 0.1 | 0.1 | 0.0 | 0.0 | 0.0 | 0.0 | 0.0 | 0.0 | 0.0 | 0.0 | 0.0 | 0.0 | 0.0 | 0.0 | 0.0 | 0.0 | 0.1 | 0.0 | 0.0 | 0.0 | 0.0 | 0.0 | 0.0 | 0.0 |
| *Prevotellamassilia* | 0.0 | 0.0 | 0.0 | 0.0 | 0.0 | 0.0 | 0.0 | 0.3 | 0.0 | 0.0 | 0.0 | 0.0 | 0.0 | 0.0 | 0.0 | 0.0 | 0.0 | 0.0 | 0.0 | 0.0 | 0.0 | 0.0 | 0.0 | 0.0 | 0.0 |
| *Povalibacter* | 0.0 | 0.0 | 0.0 | 0.2 | 0.2 | 0.0 | 0.0 | 0.0 | 0.1 | 0.0 | 0.0 | 0.0 | 0.0 | 0.2 | 0.0 | 0.0 | 0.0 | 0.0 | 0.3 | 0.0 | 0.0 | 0.1 | 0.1 | 0.0 | 0.1 |
| *Loriellopsis* | 0.0 | 0.0 | 0.0 | 0.0 | 0.1 | 0.0 | 0.0 | 0.0 | 0.0 | 0.0 | 0.0 | 0.0 | 0.0 | 0.1 | 0.0 | 0.0 | 0.1 | 0.1 | 0.4 | 0.0 | 0.0 | 0.3 | 0.0 | 0.0 | 0.0 |
| *Microbacterium* | 0.0 | 0.0 | 0.1 | 0.1 | 0.0 | 0.0 | 0.0 | 0.0 | 0.0 | 0.0 | 0.0 | 0.0 | 0.0 | 0.0 | 1.8 | 0.0 | 0.0 | 1.3 | 0.2 | 0.1 | 0.0 | 0.0 | 0.5 | 0.0 | 0.0 |
| *Methylorubrum* | 0.0 | 0.0 | 0.0 | 0.1 | 0.0 | 0.0 | 0.0 | 0.0 | 0.0 | 0.3 | 0.0 | 0.0 | 0.0 | 0.0 | 0.0 | 0.0 | 0.0 | 0.0 | 0.0 | 0.0 | 0.0 | 0.0 | 0.0 | 0.0 | 0.0 |
| *Chryseobacterium* | 0.0 | 0.0 | 1.0 | 0.0 | 0.0 | 0.0 | 0.0 | 0.0 | 0.0 | 0.0 | 0.0 | 0.0 | 0.0 | 0.0 | 0.0 | 0.0 | 0.0 | 0.1 | 0.0 | 0.0 | 0.2 | 0.7 | 2.0 | 0.1 | 0.0 |
| *Brevibacterium* | 0.0 | 0.0 | 0.0 | 0.0 | 0.0 | 0.0 | 0.0 | 0.2 | 0.0 | 0.4 | 0.0 | 0.0 | 0.4 | 0.0 | 0.0 | 0.0 | 0.0 | 0.0 | 0.0 | 0.0 | 0.0 | 0.0 | 0.6 | 0.1 | 0.0 |
| *Nocardioides* | 0.0 | 0.1 | 0.1 | 0.2 | 0.1 | 0.0 | 0.1 | 0.1 | 0.1 | 0.1 | 0.0 | 0.0 | 0.1 | 0.1 | 0.1 | 0.0 | 0.0 | 0.0 | 0.1 | 0.0 | 0.0 | 0.2 | 0.1 | 0.1 | 0.2 |
| *Mammaliicoccus* | 0.0 | 0.4 | 0.1 | 0.1 | 0.1 | 0.0 | 0.1 | 0.1 | 0.0 | 0.0 | 0.0 | 0.0 | 0.1 | 0.0 | 0.0 | 0.0 | 0.4 | 0.4 | 0.0 | 0.0 | 0.0 | 0.1 | 0.1 | 0.0 | 0.0 |
| *Lactobacillus* | 0.0 | 0.0 | 0.0 | 0.0 | 0.0 | 0.0 | 0.0 | 0.0 | 0.0 | 0.0 | 0.0 | 0.0 | 0.0 | 0.0 | 0.0 | 0.0 | 0.1 | 1.1 | 0.0 | 0.0 | 0.0 | 0.0 | 1.2 | 0.0 | 0.0 |
| *Steroidobacter* | 0.0 | 0.0 | 0.0 | 0.1 | 0.2 | 0.0 | 0.0 | 0.0 | 0.1 | 0.0 | 0.0 | 0.0 | 0.0 | 0.1 | 0.0 | 0.0 | 0.0 | 0.0 | 0.2 | 0.0 | 0.0 | 0.1 | 0.1 | 0.0 | 0.1 |
| *Rahnella* | 0.0 | 0.0 | 0.0 | 0.0 | 0.0 | 0.0 | 0.0 | 0.0 | 0.0 | 0.0 | 0.0 | 0.0 | 0.0 | 0.0 | 0.0 | 0.0 | 0.0 | 0.0 | 0.0 | 0.0 | 0.0 | 0.0 | 0.0 | 0.0 | 0.0 |
| *Macrococcus* | 0.0 | 0.2 | 0.1 | 0.1 | 0.1 | 0.0 | 0.0 | 0.1 | 0.0 | 0.0 | 0.0 | 0.0 | 0.1 | 0.0 | 0.0 | 0.0 | 0.3 | 0.3 | 0.0 | 0.0 | 0.0 | 0.1 | 0.1 | 0.0 | 0.0 |
| *Pseudonocardia* | 0.0 | 0.1 | 0.1 | 0.1 | 0.1 | 0.0 | 0.1 | 0.1 | 0.1 | 0.1 | 0.0 | 0.0 | 0.1 | 0.1 | 0.1 | 0.0 | 0.0 | 0.0 | 0.0 | 0.0 | 0.0 | 0.1 | 0.0 | 0.1 | 0.1 |
| *Alysiella* | 0.0 | 0.0 | 0.0 | 0.0 | 0.0 | 0.0 | 0.0 | 0.0 | 0.0 | 0.0 | 0.0 | 0.0 | 0.0 | 0.0 | 0.0 | 0.1 | 1.1 | 0.1 | 2.3 | 0.0 | 0.0 | 0.0 | 0.0 | 0.0 | 0.4 |
| *Dialister* | 0.0 | 0.0 | 0.0 | 0.0 | 0.0 | 0.0 | 0.0 | 0.2 | 0.0 | 0.1 | 0.0 | 0.0 | 0.0 | 0.0 | 0.0 | 0.0 | 0.0 | 0.1 | 0.0 | 0.0 | 0.0 | 0.0 | 0.0 | 0.0 | 0.0 |
| *Psychrobacter* | 0.0 | 0.0 | 0.0 | 0.0 | 0.1 | 0.0 | 0.1 | 0.3 | 0.1 | 0.2 | 0.0 | 1.3 | 0.2 | 0.0 | 0.0 | 0.0 | 0.0 | 0.1 | 0.0 | 0.0 | 0.0 | 0.0 | 0.2 | 0.0 | 0.0 |
| *Pantoea* | 0.0 | 0.0 | 0.0 | 0.0 | 0.0 | 0.0 | 0.0 | 0.0 | 1.1 | 0.0 | 0.0 | 0.0 | 0.0 | 0.0 | 0.0 | 0.0 | 0.0 | 0.0 | 0.0 | 2.2 | 0.0 | 0.0 | 0.0 | 0.0 | 0.0 |
| *Cupriavidus* | 0.0 | 0.0 | 0.0 | 0.0 | 0.0 | 0.0 | 0.0 | 0.0 | 0.0 | 0.0 | 0.0 | 0.0 | 0.0 | 0.2 | 0.2 | 0.0 | 0.0 | 0.0 | 0.5 | 0.0 | 0.0 | 0.1 | 0.1 | 0.0 | 0.1 |
| *Simonsiella* | 0.0 | 0.0 | 0.0 | 0.0 | 0.1 | 0.0 | 0.0 | 0.0 | 0.0 | 0.0 | 0.0 | 0.0 | 0.0 | 0.0 | 0.0 | 0.0 | 0.5 | 0.1 | 0.7 | 0.0 | 0.0 | 0.0 | 0.0 | 0.0 | 0.0 |
| *Lautropia* | 0.0 | 0.0 | 0.0 | 0.0 | 0.0 | 0.0 | 0.2 | 0.1 | 0.0 | 0.1 | 0.0 | 0.0 | 0.0 | 0.1 | 0.0 | 0.0 | 0.0 | 0.0 | 0.0 | 0.0 | 0.0 | 0.1 | 0.3 | 1.0 | 0.8 |
| *Erwinia* | 0.0 | 0.0 | 0.0 | 0.0 | 0.0 | 0.0 | 0.0 | 0.0 | 0.0 | 0.0 | 0.0 | 0.0 | 0.0 | 0.0 | 0.0 | 0.0 | 0.0 | 0.0 | 0.0 | 2.0 | 0.0 | 0.0 | 0.0 | 0.0 | 0.0 |
| *Nigerium* | 0.0 | 0.0 | 0.1 | 0.2 | 0.2 | 0.0 | 0.0 | 0.1 | 0.1 | 0.1 | 0.0 | 0.0 | 0.1 | 0.1 | 0.1 | 0.0 | 0.0 | 0.1 | 0.1 | 0.0 | 0.0 | 0.1 | 0.1 | 0.1 | 0.1 |
| *Aliterella* | 0.0 | 0.0 | 0.0 | 0.0 | 0.0 | 0.0 | 0.0 | 0.0 | 0.0 | 0.0 | 0.0 | 0.0 | 0.0 | 0.1 | 0.0 | 0.0 | 0.1 | 0.0 | 0.2 | 0.0 | 0.0 | 0.4 | 0.0 | 0.1 | 0.0 |
| *Aggregatibacter* | 0.0 | 0.0 | 0.0 | 0.0 | 0.0 | 0.0 | 0.3 | 0.1 | 0.0 | 0.0 | 0.0 | 0.0 | 0.4 | 0.0 | 0.0 | 0.0 | 0.0 | 0.0 | 0.0 | 0.0 | 0.0 | 0.0 | 0.0 | 0.1 | 0.0 |
| *Dietzia* | 0.0 | 0.1 | 0.1 | 0.0 | 0.1 | 0.0 | 0.1 | 0.1 | 0.0 | 0.1 | 0.0 | 0.0 | 0.1 | 0.0 | 0.0 | 0.0 | 0.0 | 0.0 | 0.0 | 0.0 | 0.0 | 0.0 | 0.0 | 0.0 | 0.0 |
| *Bifidobacterium* | 0.0 | 0.0 | 0.0 | 0.0 | 0.0 | 0.0 | 0.1 | 0.1 | 0.0 | 0.0 | 0.0 | 0.0 | 0.0 | 0.0 | 0.0 | 0.0 | 0.0 | 0.0 | 0.0 | 0.0 | 0.0 | 0.0 | 0.0 | 0.0 | 0.0 |
| *Vitreoscilla* | 0.0 | 0.0 | 0.0 | 0.0 | 0.1 | 0.0 | 0.0 | 0.0 | 0.0 | 0.0 | 0.0 | 0.0 | 0.0 | 0.0 | 0.0 | 0.0 | 0.0 | 0.0 | 0.1 | 0.0 | 0.0 | 0.0 | 0.0 | 0.0 | 0.0 |
| *Tsukamurella* | 0.0 | 0.0 | 0.1 | 0.1 | 0.1 | 0.0 | 0.1 | 0.2 | 0.1 | 0.1 | 0.0 | 0.0 | 0.1 | 0.0 | 0.0 | 0.0 | 0.0 | 0.0 | 0.0 | 0.0 | 0.0 | 0.0 | 0.0 | 0.0 | 0.0 |
| *Schaalia* | 0.0 | 0.0 | 0.0 | 0.0 | 0.0 | 0.0 | 0.1 | 0.1 | 0.1 | 0.0 | 0.0 | 0.0 | 0.0 | 1.0 | 0.0 | 0.0 | 0.0 | 0.0 | 0.0 | 0.0 | 0.0 | 0.0 | 0.0 | 0.2 | 0.0 |
| *Gloeocapsopsis* | 0.0 | 0.0 | 0.0 | 0.0 | 0.0 | 0.0 | 0.0 | 0.0 | 0.0 | 0.1 | 0.0 | 0.0 | 0.0 | 0.1 | 0.0 | 0.0 | 0.1 | 0.0 | 0.1 | 0.0 | 0.0 | 0.1 | 0.1 | 0.0 | 0.0 |
| *Klebsiella* | 0.0 | 0.0 | 0.0 | 0.0 | 0.0 | 0.0 | 0.0 | 0.0 | 0.0 | 0.0 | 0.0 | 0.0 | 0.0 | 0.0 | 0.0 | 0.0 | 0.3 | 0.1 | 0.0 | 0.2 | 0.0 | 0.0 | 0.0 | 0.0 | 0.0 |
| *Rhizobium* | 0.0 | 0.0 | 0.0 | 0.0 | 0.0 | 0.0 | 0.0 | 0.0 | 0.5 | 0.2 | 0.0 | 0.2 | 0.0 | 0.1 | 0.0 | 0.0 | 0.0 | 0.1 | 0.0 | 0.0 | 0.0 | 0.0 | 0.0 | 0.0 | 0.7 |
| *Nocardia* | 0.0 | 0.0 | 0.0 | 0.0 | 0.1 | 0.0 | 0.1 | 0.1 | 0.4 | 0.3 | 0.0 | 0.0 | 0.0 | 0.1 | 0.0 | 0.0 | 0.0 | 0.1 | 0.0 | 0.0 | 0.0 | 0.0 | 0.0 | 0.0 | 0.0 |
| *Brachybacterium* | 0.0 | 0.0 | 0.1 | 0.0 | 0.0 | 0.0 | 0.1 | 0.1 | 0.0 | 0.0 | 0.0 | 0.0 | 0.0 | 0.6 | 0.0 | 0.0 | 0.0 | 0.0 | 0.0 | 0.0 | 0.0 | 0.0 | 0.0 | 0.0 | 0.0 |
| *Janibacter* | 0.0 | 0.0 | 0.0 | 0.0 | 0.0 | 0.0 | 0.1 | 0.0 | 0.4 | 0.1 | 0.0 | 0.2 | 0.2 | 0.0 | 0.0 | 0.0 | 0.0 | 0.1 | 0.0 | 0.0 | 0.0 | 0.0 | 0.0 | 0.0 | 0.0 |
| *Lysobacter* | 0.0 | 0.0 | 0.0 | 0.0 | 0.0 | 0.0 | 0.0 | 0.1 | 0.0 | 0.0 | 0.0 | 0.0 | 0.0 | 1.8 | 0.0 | 0.0 | 0.0 | 0.0 | 0.0 | 0.0 | 0.0 | 0.0 | 0.0 | 0.0 | 0.0 |
| *Facklamia* | 0.0 | 0.0 | 0.0 | 0.0 | 0.0 | 0.0 | 0.1 | 0.0 | 0.0 | 0.0 | 0.0 | 0.0 | 0.0 | 0.0 | 0.1 | 0.0 | 0.0 | 0.0 | 0.0 | 0.0 | 0.0 | 0.0 | 0.0 | 0.0 | 0.0 |
| *Sphingopyxis* | 0.0 | 0.0 | 0.0 | 0.0 | 0.0 | 0.0 | 0.0 | 0.0 | 0.0 | 0.0 | 0.0 | 0.0 | 0.0 | 0.0 | 0.0 | 0.0 | 0.0 | 0.0 | 0.0 | 0.0 | 0.0 | 0.0 | 0.0 | 0.0 | 0.0 |
| *Flavobacterium* | 0.0 | 0.0 | 0.0 | 0.0 | 0.0 | 0.0 | 0.0 | 0.0 | 0.0 | 0.0 | 0.0 | 0.0 | 0.0 | 0.0 | 0.0 | 0.0 | 0.0 | 0.0 | 0.0 | 0.0 | 0.0 | 0.0 | 0.0 | 0.0 | 0.0 |
| *Stenotrophomonas* | 0.0 | 0.0 | 0.0 | 0.0 | 0.0 | 0.0 | 0.0 | 1.5 | 0.8 | 0.4 | 0.0 | 0.0 | 0.0 | 0.0 | 0.0 | 0.0 | 0.0 | 0.0 | 0.0 | 0.0 | 0.0 | 0.0 | 0.4 | 0.0 | 0.0 |
| *Afipia* | 0.0 | 0.0 | 0.0 | 0.1 | 0.0 | 0.0 | 0.0 | 0.0 | 0.1 | 0.1 | 0.0 | 0.0 | 0.0 | 0.1 | 0.1 | 0.0 | 0.0 | 0.0 | 0.0 | 0.0 | 0.0 | 0.0 | 0.0 | 0.0 | 0.0 |
| *Pseudoglutamicibacter* | 0.0 | 0.0 | 0.0 | 0.0 | 0.1 | 0.0 | 0.1 | 0.0 | 0.0 | 0.1 | 0.0 | 0.0 | 0.0 | 0.0 | 0.0 | 0.0 | 0.0 | 0.0 | 0.0 | 0.0 | 0.0 | 0.0 | 0.0 | 0.0 | 0.0 |
| *Hydrobacter* | 0.0 | 0.0 | 0.0 | 0.0 | 0.0 | 0.0 | 0.0 | 0.0 | 0.0 | 0.0 | 0.0 | 0.0 | 0.1 | 0.1 | 0.0 | 0.0 | 0.0 | 0.1 | 0.0 | 0.0 | 0.0 | 0.0 | 0.0 | 0.0 | 0.0 |
| *Dermabacter* | 0.0 | 0.0 | 0.9 | 0.0 | 0.0 | 0.0 | 0.0 | 0.0 | 0.0 | 0.0 | 0.0 | 0.0 | 0.0 | 0.0 | 0.0 | 0.0 | 0.0 | 0.0 | 0.0 | 0.0 | 0.0 | 0.0 | 0.0 | 0.0 | 0.0 |
| *Pectobacterium* | 0.0 | 0.0 | 0.0 | 0.0 | 0.0 | 0.0 | 0.0 | 0.0 | 0.0 | 0.0 | 0.0 | 0.0 | 0.0 | 0.0 | 0.0 | 0.0 | 0.0 | 0.0 | 0.0 | 0.0 | 0.0 | 0.0 | 0.0 | 0.0 | 0.0 |
| *Stanieria* | 0.0 | 0.0 | 0.0 | 0.0 | 0.1 | 0.0 | 0.0 | 0.0 | 0.0 | 0.0 | 0.0 | 0.0 | 0.0 | 0.0 | 0.0 | 0.0 | 0.1 | 0.0 | 0.2 | 0.0 | 0.0 | 0.3 | 0.0 | 0.0 | 0.0 |
| *Capnocytophaga* | 0.0 | 0.0 | 0.0 | 0.0 | 0.0 | 0.0 | 0.3 | 0.1 | 0.0 | 0.0 | 0.0 | 0.0 | 0.0 | 0.0 | 0.0 | 0.0 | 0.0 | 0.0 | 0.0 | 0.0 | 0.0 | 0.4 | 0.0 | 0.1 | 0.0 |
| Other genera | 4.2 | 4.0 | 5.4 | 3.4 | 5.4 | 6.5 | 5.8 | 4.3 | 4.2 | 4.9 | 2.3 | 2.7 | 3.5 | 8.7 | 4.8 | 8.5 | 6.1 | 5.6 | 6.6 | 29.7 | 2.9 | 3.9 | 3.4 | 3.4 | 5.4 |

| | Subject_16 | | | | | Subject_17 | | | | | Subject_18 | | | | | Subject_20 | | | | | Subject_22 | | | | |
|---|---|---|---|---|---|---|---|---|---|---|---|---|---|---|---|---|---|---|---|---|---|---|---|---|---|
| Taxa | Baseline | Week 2 | Week 4 | Week 8 | Week 12 | Baseline | Week 2 | Week 4 | Week 8 | Week 12 | Baseline | Week 2 | Week 4 | Week 8 | Week 12 | Baseline | Week 2 | Week 4 | Week 8 | Week 12 | Baseline | Week 2 | Week 4 | Week 8 | Week 12 |
| *Cutibacterium* | 32.2 | 33.3 | 31.2 | 31.8 | 30.7 | 31.6 | 34.0 | 50.9 | 62.3 | 66.9 | 23.1 | 46.6 | 46.9 | 38.0 | 50.1 | 46.7 | 51.1 | 62.8 | 61.5 | 59.0 | 17.1 | 26.7 | 28.1 | 32.9 | 30.4 |
| *Staphylococcus* | 35.7 | 25.6 | 18.1 | 12.8 | 21.7 | 36.6 | 25.2 | 21.7 | 16.9 | 16.1 | 66.7 | 28.2 | 21.6 | 15.1 | 13.2 | 42.7 | 29.9 | 16.0 | 12.0 | 13.6 | 63.9 | 53.4 | 40.8 | 20.5 | 24.7 |
| *Corynebacterium* | 23.9 | 26.0 | 25.6 | 22.7 | 23.2 | 4.1 | 5.6 | 10.3 | 3.3 | 4.5 | 4.9 | 9.9 | 8.2 | 14.0 | 3.1 | 0.8 | 0.7 | 0.9 | 0.8 | 0.6 | 2.4 | 2.8 | 4.3 | 5.9 | 16.8 |
| *Streptococcus* | 3.2 | 3.0 | 3.7 | 4.0 | 2.5 | 15.2 | 15.1 | 2.0 | 1.1 | 2.3 | 1.9 | 1.8 | 3.4 | 0.0 | 1.2 | 5.9 | 5.4 | 3.3 | 8.0 | 15.7 | 3.3 | 3.6 | 1.2 | 0.8 | 3.1 |
| *Snodgrassella* | 0.4 | 0.6 | 1.0 | 0.2 | 0.5 | 0.0 | 0.0 | 0.0 | 0.0 | 0.5 | 1.1 | 0.3 | 1.8 | 6.8 | 8.2 | 0.7 | 1.4 | 1.1 | 4.1 | 1.6 | 0.0 | 0.0 | 0.0 | 0.4 | 1.4 |
| *Moraxella* | 0.2 | 0.0 | 0.5 | 1.3 | 0.1 | 0.0 | 0.1 | 0.0 | 0.0 | 0.0 | 0.0 | 0.5 | 0.7 | 0.0 | 0.0 | 0.4 | 0.6 | 7.7 | 1.7 | 0.5 | 1.0 | 1.4 | 12.9 | 11.6 | 2.4 |
| *Finegoldia* | 0.2 | 0.1 | 0.0 | 0.3 | 0.0 | 7.8 | 4.7 | 4.3 | 2.6 | 2.2 | 0.0 | 1.0 | 0.4 | 0.0 | 0.0 | 0.1 | 0.3 | 0.2 | 0.2 | 0.0 | 0.0 | 0.0 | 0.1 | 0.8 | 0.0 |
| *Acinetobacter* | 0.3 | 0.2 | 0.0 | 0.1 | 0.8 | 0.0 | 0.1 | 0.0 | 2.8 | 0.0 | 0.0 | 0.0 | 0.0 | 0.0 | 0.0 | 0.0 | 0.0 | 0.1 | 0.4 | 0.1 | 0.0 | 0.0 | 0.1 | 0.1 | 0.0 |
| *Deinococcus* | 0.0 | 0.0 | 0.4 | 0.0 | 0.0 | 0.0 | 0.0 | 0.0 | 0.0 | 0.0 | 0.0 | 0.0 | 0.0 | 0.0 | 0.0 | 0.0 | 0.0 | 0.2 | 0.0 | 0.0 | 0.7 | 0.8 | 3.3 | 4.8 | 5.3 |
| *Roseomonas* | 0.0 | 0.0 | 0.0 | 0.2 | 0.0 | 0.0 | 0.0 | 0.0 | 0.0 | 0.0 | 0.0 | 0.0 | 0.0 | 0.0 | 0.0 | 0.0 | 0.0 | 0.4 | 0.0 | 0.1 | 0.0 | 0.0 | 0.0 | 0.0 | 1.9 |
| *Neisseria* | 0.0 | 0.1 | 0.3 | 0.0 | 2.0 | 0.1 | 0.7 | 0.0 | 1.0 | 0.1 | 0.0 | 0.1 | 0.3 | 2.1 | 8.5 | 0.0 | 0.3 | 0.3 | 1.0 | 0.3 | 0.0 | 0.5 | 0.0 | 0.1 | 0.2 |
| *Rothia* | 0.0 | 0.2 | 0.0 | 0.7 | 0.8 | 0.0 | 0.2 | 0.0 | 0.0 | 0.1 | 0.2 | 0.0 | 0.0 | 0.0 | 0.0 | 0.0 | 0.2 | 0.1 | 0.2 | 0.4 | 0.0 | 0.3 | 0.1 | 0.4 | 0.0 |
| *Prevotella* | 0.0 | 0.1 | 1.7 | 0.8 | 1.9 | 0.1 | 1.5 | 0.0 | 0.0 | 0.0 | 0.0 | 0.2 | 0.0 | 0.0 | 0.0 | 0.1 | 0.3 | 0.1 | 1.0 | 0.7 | 0.1 | 0.5 | 0.1 | 0.0 | 3.5 |
| *Anaerococcus* | 0.0 | 0.0 | 0.4 | 0.0 | 0.5 | 0.0 | 1.0 | 2.7 | 1.4 | 0.0 | 0.1 | 0.8 | 4.2 | 0.0 | 0.0 | 0.0 | 0.3 | 0.0 | 0.1 | 0.0 | 0.0 | 0.7 | 0.1 | 0.0 | 0.0 |
| *Kocuria* | 0.0 | 0.1 | 0.1 | 0.1 | 0.2 | 0.0 | 0.2 | 0.5 | 0.0 | 2.3 | 0.0 | 0.0 | 0.0 | 0.0 | 0.0 | 0.1 | 0.2 | 0.1 | 0.1 | 0.1 | 0.0 | 0.1 | 0.0 | 0.0 | 1.7 |
| *Haemophilus* | 0.0 | 0.0 | 0.7 | 0.0 | 0.0 | 0.0 | 1.7 | 0.0 | 0.0 | 0.0 | 0.0 | 0.0 | 0.0 | 0.0 | 0.0 | 0.0 | 0.0 | 0.0 | 0.0 | 0.0 | 0.1 | 0.8 | 0.6 | 0.0 | 0.0 |
| *Paracoccus* | 0.0 | 0.0 | 0.2 | 0.0 | 0.0 | 0.0 | 0.1 | 0.0 | 0.0 | 0.0 | 0.0 | 0.0 | 0.0 | 0.0 | 0.0 | 0.0 | 0.0 | 0.0 | 0.0 | 0.0 | 0.0 | 0.0 | 0.0 | 0.0 | 0.0 |
| *Lawsonella* | 0.0 | 1.6 | 0.2 | 0.2 | 0.9 | 0.1 | 0.6 | 1.7 | 0.3 | 0.0 | 0.0 | 0.3 | 0.6 | 0.0 | 0.0 | 0.1 | 0.1 | 0.1 | 0.0 | 0.0 | 0.0 | 0.0 | 0.1 | 0.0 | 0.0 |
| *Bacillus* | 0.2 | 0.6 | 0.3 | 3.5 | 1.0 | 0.0 | 0.2 | 0.1 | 0.0 | 0.0 | 0.0 | 1.5 | 1.1 | 0.1 | 0.0 | 0.0 | 0.3 | 0.1 | 0.0 | 0.1 | 0.0 | 1.2 | 0.9 | 0.1 | 1.1 |
| *Methylobacterium* | 0.1 | 0.0 | 2.8 | 0.4 | 0.0 | 0.0 | 0.1 | 0.0 | 0.0 | 0.0 | 0.0 | 0.0 | 0.0 | 4.1 | 0.0 | 0.0 | 0.0 | 0.2 | 0.1 | 0.0 | 0.0 | 0.0 | 0.0 | 0.0 | 0.0 |
| *Veillonella* | 0.0 | 0.4 | 0.5 | 0.5 | 1.1 | 0.0 | 0.7 | 1.7 | 0.0 | 0.0 | 0.0 | 0.0 | 0.0 | 0.0 | 0.0 | 0.1 | 2.8 | 0.1 | 0.2 | 1.2 | 0.0 | 0.1 | 0.1 | 0.6 | 0.0 |
| *Granulicatella* | 0.0 | 0.1 | 0.7 | 0.3 | 0.0 | 0.0 | 0.9 | 0.0 | 0.0 | 0.0 | 0.0 | 0.0 | 0.0 | 0.0 | 0.0 | 0.0 | 0.2 | 0.1 | 0.1 | 0.0 | 0.0 | 0.2 | 0.1 | 0.0 | 0.0 |
| *Sphingomonas* | 0.0 | 0.0 | 0.0 | 0.8 | 0.0 | 0.0 | 0.0 | 0.0 | 0.0 | 0.0 | 0.0 | 0.0 | 0.0 | 0.0 | 0.0 | 0.0 | 0.0 | 0.8 | 0.2 | 0.1 | 0.0 | 0.1 | 0.1 | 0.6 | 0.0 |
| *Williamsia* | 0.0 | 0.1 | 0.0 | 0.0 | 0.0 | 0.0 | 0.0 | 0.0 | 0.0 | 0.0 | 0.0 | 0.0 | 0.0 | 0.0 | 0.0 | 0.0 | 0.0 | 0.0 | 0.0 | 0.0 | 0.0 | 0.0 | 0.0 | 0.0 | 0.0 |
| *Peptoniphilus* | 0.0 | 0.0 | 0.0 | 0.4 | 1.0 | 0.0 | 0.8 | 0.0 | 0.3 | 0.0 | 0.2 | 1.8 | 1.5 | 4.2 | 0.0 | 0.0 | 0.2 | 0.1 | 0.2 | 0.1 | 0.0 | 0.0 | 0.0 | 0.0 | 0.0 |
| *Arachnia* | 0.0 | 0.3 | 0.2 | 0.1 | 0.2 | 0.1 | 0.2 | 0.3 | 0.4 | 0.4 | 0.0 | 0.1 | 0.3 | 0.2 | 0.1 | 0.0 | 0.3 | 0.3 | 0.3 | 0.2 | 0.0 | 0.2 | 0.1 | 0.2 | 0.1 |
| *Escherichia* | 0.0 | 0.0 | 0.0 | 0.0 | 0.0 | 0.0 | 0.0 | 0.0 | 0.0 | 0.0 | 0.0 | 0.0 | 0.0 | 0.0 | 0.0 | 0.0 | 0.0 | 0.0 | 0.0 | 0.0 | 0.0 | 0.0 | 0.0 | 0.0 | 0.0 |
| *Skermanella* | 0.0 | 0.0 | 0.0 | 0.0 | 0.0 | 0.0 | 0.0 | 0.0 | 0.0 | 0.0 | 0.0 | 0.0 | 0.0 | 0.0 | 0.0 | 0.0 | 0.0 | 0.2 | 0.0 | 0.0 | 0.0 | 0.0 | 0.0 | 0.0 | 0.0 |
| *Microcoleus* | 0.0 | 0.1 | 0.2 | 0.0 | 0.1 | 0.0 | 0.0 | 0.0 | 0.3 | 0.0 | 0.0 | 0.0 | 0.0 | 0.1 | 0.0 | 0.0 | 0.0 | 0.3 | 0.2 | 0.0 | 0.0 | 0.0 | 0.0 | 0.0 | 0.0 |
| *Serratia* | 0.0 | 0.0 | 0.0 | 0.4 | 0.0 | 0.0 | 0.1 | 0.0 | 0.0 | 0.0 | 0.0 | 0.0 | 0.0 | 0.0 | 0.0 | 0.0 | 0.0 | 0.0 | 0.0 | 0.0 | 0.0 | 0.0 | 0.1 | 0.0 | 0.0 |
| *Pseudomonas* | 0.0 | 0.0 | 0.1 | 0.0 | 0.0 | 0.0 | 0.1 | 0.0 | 0.0 | 0.0 | 0.0 | 0.0 | 0.0 | 7.4 | 0.0 | 0.0 | 0.1 | 0.5 | 1.0 | 0.2 | 0.0 | 0.0 | 0.0 | 0.0 | 0.0 |
| *Gemella* | 0.0 | 0.1 | 0.0 | 0.0 | 0.4 | 0.0 | 0.2 | 0.0 | 0.0 | 0.0 | 0.0 | 0.0 | 0.0 | 0.0 | 0.0 | 0.0 | 0.0 | 0.0 | 0.1 | 0.0 | 0.0 | 0.1 | 0.1 | 0.0 | 0.0 |
| *Porphyromonas* | 0.0 | 0.1 | 0.0 | 0.0 | 0.0 | 0.0 | 0.0 | 0.0 | 0.0 | 0.0 | 0.0 | 0.0 | 0.0 | 0.0 | 0.0 | 0.0 | 0.0 | 0.0 | 0.1 | 0.1 | 0.0 | 0.0 | 0.0 | 0.0 | 0.0 |
| *Abiotrophia* | 0.0 | 0.0 | 0.0 | 0.0 | 0.0 | 0.0 | 0.0 | 0.0 | 0.0 | 0.0 | 0.0 | 0.0 | 0.0 | 0.0 | 0.0 | 0.0 | 0.0 | 0.0 | 0.0 | 0.0 | 0.0 | 0.0 | 0.0 | 0.0 | 0.0 |
| *Mycolicibacterium* | 0.0 | 0.1 | 0.1 | 0.1 | 0.1 | 0.1 | 0.1 | 0.1 | 0.1 | 0.1 | 0.1 | 0.1 | 0.7 | 0.6 | 0.0 | 0.0 | 0.0 | 0.1 | 0.0 | 0.0 | 0.0 | 0.0 | 0.0 | 0.5 | 0.1 |
| *Enterococcus* | 0.0 | 0.1 | 0.1 | 0.1 | 0.1 | 0.0 | 0.4 | 0.0 | 0.0 | 0.0 | 0.0 | 0.1 | 0.2 | 0.0 | 0.0 | 0.0 | 0.1 | 0.0 | 0.1 | 0.2 | 0.0 | 0.2 | 0.1 | 0.0 | 0.1 |
| *Actinomyces* | 0.0 | 0.1 | 0.0 | 0.3 | 0.3 | 0.0 | 0.1 | 0.0 | 0.0 | 0.0 | 0.0 | 0.0 | 0.0 | 0.0 | 0.0 | 0.0 | 0.3 | 0.0 | 0.0 | 0.8 | 0.0 | 0.0 | 0.0 | 0.0 | 0.0 |
| *Massilia* | 0.0 | 0.0 | 0.9 | 0.8 | 0.0 | 0.0 | 0.0 | 0.0 | 0.0 | 0.0 | 0.0 | 0.0 | 0.0 | 0.0 | 0.0 | 0.0 | 0.0 | 0.0 | 0.3 | 0.0 | 0.0 | 0.0 | 0.0 | 0.0 | 0.0 |
| *Brevundimonas* | 0.0 | 0.0 | 0.0 | 0.3 | 0.0 | 0.0 | 0.3 | 0.0 | 0.4 | 0.0 | 0.0 | 0.0 | 0.1 | 0.0 | 0.0 | 0.0 | 0.0 | 0.0 | 0.0 | 0.1 | 0.0 | 0.0 | 0.0 | 1.5 | 0.0 |
| *Micrococcus* | 0.0 | 0.0 | 0.0 | 0.0 | 0.0 | 0.0 | 0.0 | 0.2 | 0.0 | 0.0 | 0.0 | 0.0 | 0.0 | 0.0 | 0.0 | 0.0 | 0.0 | 0.0 | 0.1 | 0.1 | 0.0 | 0.0 | 0.0 | 0.0 | 0.0 |
| *Kingella* | 0.0 | 0.0 | 0.0 | 0.0 | 0.0 | 0.0 | 0.0 | 0.0 | 0.0 | 0.0 | 0.0 | 0.0 | 0.1 | 0.6 | 2.7 | 0.0 | 0.1 | 0.0 | 0.2 | 0.1 | 0.0 | 0.0 | 0.0 | 0.0 | 0.1 |
| *Dermacoccus* | 0.0 | 0.0 | 0.0 | 0.0 | 0.0 | 0.0 | 0.0 | 0.0 | 0.0 | 0.0 | 0.0 | 0.0 | 0.0 | 0.0 | 0.0 | 0.0 | 0.0 | 0.0 | 0.0 | 0.0 | 0.0 | 0.0 | 0.0 | 0.0 | 0.0 |
| *Leptotrichia* | 0.0 | 0.3 | 0.0 | 0.2 | 0.0 | 0.0 | 0.2 | 0.0 | 0.0 | 0.0 | 0.0 | 0.0 | 0.0 | 0.0 | 0.0 | 0.0 | 0.0 | 0.1 | 0.0 | 0.0 | 0.0 | 0.0 | 0.0 | 0.0 | 0.0 |
| *Fusobacterium* | 0.0 | 0.1 | 0.8 | 0.0 | 0.0 | 0.0 | 0.1 | 0.0 | 0.0 | 0.0 | 0.0 | 0.0 | 0.0 | 0.0 | 0.0 | 0.0 | 0.1 | 0.0 | 0.2 | 0.2 | 0.0 | 0.0 | 0.0 | 0.0 | 0.0 |
| *Eikenella* | 0.0 | 0.0 | 0.1 | 0.0 | 0.0 | 0.0 | 0.0 | 0.0 | 0.0 | 0.0 | 0.0 | 0.0 | 0.1 | 0.6 | 2.7 | 0.0 | 0.0 | 0.0 | 0.1 | 0.1 | 0.0 | 0.0 | 0.0 | 0.0 | 0.1 |
| *Mycobacterium* | 0.0 | 0.2 | 0.1 | 0.1 | 0.1 | 0.1 | 0.0 | 0.1 | 0.0 | 0.1 | 0.0 | 0.0 | 0.1 | 1.3 | 0.0 | 0.0 | 0.0 | 0.2 | 0.0 | 0.0 | 0.0 | 0.0 | 0.0 | 0.2 | 0.1 |
| *Gordonia* | 0.0 | 0.1 | 0.1 | 0.1 | 0.1 | 0.0 | 0.1 | 0.1 | 0.0 | 0.0 | 0.0 | 0.0 | 0.0 | 0.1 | 0.0 | 0.0 | 0.0 | 0.0 | 0.0 | 0.0 | 0.0 | 0.0 | 0.0 | 0.0 | 0.0 |
| *Tessaracoccus* | 0.0 | 0.1 | 0.1 | 0.0 | 0.1 | 0.0 | 0.1 | 0.2 | 0.2 | 0.2 | 0.0 | 0.1 | 0.1 | 0.1 | 0.0 | 0.0 | 0.2 | 0.1 | 0.1 | 0.1 | 0.0 | 0.1 | 0.0 | 0.1 | 0.1 |
| *Rhodococcus* | 0.0 | 0.2 | 0.3 | 0.2 | 0.2 | 0.0 | 0.1 | 0.1 | 0.0 | 0.0 | 0.0 | 0.1 | 0.1 | 0.1 | 0.0 | 0.0 | 0.0 | 0.0 | 0.0 | 0.0 | 0.0 | 0.0 | 0.0 | 0.3 | 0.1 |
| *Metabacillus* | 0.0 | 0.2 | 0.0 | 0.0 | 0.0 | 0.0 | 0.0 | 0.0 | 0.0 | 0.0 | 0.0 | 0.1 | 0.0 | 0.0 | 0.0 | 0.0 | 0.0 | 0.0 | 0.0 | 0.0 | 0.0 | 0.1 | 0.1 | 0.0 | 2.0 |
| *Prevotellamassilia* | 0.0 | 0.0 | 0.0 | 0.0 | 0.0 | 0.0 | 0.0 | 0.0 | 0.0 | 0.0 | 0.0 | 0.0 | 0.0 | 0.0 | 0.0 | 0.0 | 0.0 | 0.0 | 0.0 | 0.0 | 0.0 | 1.5 | 0.5 | 0.0 | 0.0 |
| *Povalibacter* | 0.0 | 0.0 | 0.3 | 0.3 | 0.4 | 0.0 | 0.0 | 0.0 | 0.8 | 0.2 | 0.0 | 0.0 | 0.3 | 0.0 | 0.5 | 0.0 | 0.0 | 0.0 | 0.0 | 0.0 | 0.0 | 0.0 | 0.0 | 0.0 | 0.3 |
| *Loriellopsis* | 0.0 | 0.1 | 0.0 | 0.1 | 0.0 | 0.0 | 0.0 | 0.0 | 0.0 | 0.0 | 0.0 | 0.0 | 0.0 | 0.0 | 0.0 | 0.0 | 0.0 | 0.3 | 0.1 | 0.0 | 0.0 | 0.0 | 0.0 | 0.0 | 0.0 |
| *Microbacterium* | 0.0 | 0.0 | 0.0 | 1.9 | 0.0 | 0.0 | 0.0 | 0.0 | 0.3 | 0.0 | 0.0 | 0.0 | 0.7 | 0.0 | 0.0 | 0.0 | 0.0 | 0.0 | 0.1 | 0.0 | 0.0 | 0.0 | 0.0 | 0.0 | 0.0 |
| *Methylorubrum* | 0.0 | 0.0 | 0.0 | 0.8 | 0.0 | 0.0 | 0.0 | 0.0 | 0.0 | 0.0 | 0.0 | 0.0 | 0.0 | 0.0 | 0.0 | 0.0 | 0.0 | 0.1 | 0.1 | 0.0 | 0.0 | 0.0 | 0.0 | 0.0 | 0.0 |
| *Chryseobacterium* | 0.0 | 0.0 | 0.4 | 0.5 | 0.0 | 0.0 | 0.0 | 0.0 | 0.0 | 0.0 | 0.0 | 0.0 | 0.0 | 0.0 | 0.0 | 0.0 | 0.0 | 0.0 | 0.0 | 0.0 | 0.0 | 0.0 | 0.0 | 0.0 | 0.0 |
| *Brevibacterium* | 0.0 | 0.0 | 0.0 | 1.1 | 1.5 | 0.0 | 0.0 | 0.0 | 0.0 | 0.0 | 0.0 | 0.0 | 0.0 | 0.0 | 0.0 | 0.0 | 0.0 | 0.0 | 0.0 | 0.0 | 0.0 | 0.0 | 0.1 | 0.0 | 0.0 |
| *Nocardioides* | 0.0 | 0.1 | 0.1 | 0.0 | 0.1 | 0.0 | 0.1 | 0.1 | 0.2 | 0.2 | 0.0 | 0.1 | 0.1 | 0.1 | 0.1 | 0.0 | 0.1 | 0.1 | 0.1 | 0.2 | 0.0 | 0.1 | 0.0 | 0.1 | 0.1 |
| *Mammaliicoccus* | 0.0 | 0.1 | 0.1 | 0.0 | 0.1 | 0.0 | 0.1 | 0.0 | 0.0 | 0.0 | 0.0 | 0.7 | 0.5 | 0.0 | 0.0 | 0.0 | 0.1 | 0.0 | 0.0 | 0.0 | 0.0 | 0.3 | 0.3 | 0.0 | 0.1 |
| *Lactobacillus* | 0.0 | 0.0 | 0.0 | 0.0 | 0.0 | 0.0 | 0.0 | 0.0 | 0.0 | 0.0 | 0.0 | 0.0 | 0.0 | 0.0 | 0.0 | 0.0 | 0.0 | 0.0 | 0.1 | 0.0 | 0.0 | 0.0 | 0.0 | 0.0 | 0.0 |
| *Steroidobacter* | 0.0 | 0.0 | 0.3 | 0.2 | 0.4 | 0.0 | 0.0 | 0.0 | 0.6 | 0.2 | 0.0 | 0.0 | 0.3 | 0.0 | 0.4 | 0.0 | 0.0 | 0.0 | 0.0 | 0.0 | 0.0 | 0.0 | 0.0 | 0.0 | 0.3 |
| *Rahnella* | 0.0 | 0.1 | 0.0 | 0.0 | 0.0 | 0.0 | 0.0 | 0.0 | 0.0 | 0.0 | 0.0 | 0.0 | 0.0 | 0.0 | 0.0 | 0.0 | 0.0 | 0.0 | 0.0 | 0.0 | 0.0 | 0.0 | 0.0 | 0.0 | 0.0 |
| *Macrococcus* | 0.1 | 0.1 | 0.1 | 0.1 | 0.1 | 0.0 | 0.0 | 0.0 | 0.0 | 0.0 | 0.0 | 0.5 | 0.4 | 0.0 | 0.0 | 0.0 | 0.1 | 0.0 | 0.0 | 0.0 | 0.0 | 0.4 | 0.3 | 0.0 | 0.1 |
| *Pseudonocardia* | 0.0 | 0.2 | 0.1 | 0.1 | 0.1 | 0.0 | 0.1 | 0.1 | 0.1 | 0.1 | 0.0 | 0.0 | 0.1 | 0.0 | 0.3 | 0.0 | 0.0 | 0.0 | 0.0 | 0.1 | 0.0 | 0.0 | 0.0 | 0.0 | 0.1 |
| *Alysiella* | 0.0 | 0.1 | 0.1 | 0.0 | 0.0 | 0.0 | 0.0 | 0.0 | 0.0 | 0.0 | 0.0 | 0.0 | 0.0 | 0.2 | 0.5 | 0.0 | 0.0 | 0.0 | 0.0 | 0.0 | 0.0 | 0.0 | 0.0 | 0.0 | 0.0 |
| *Dialister* | 0.0 | 0.0 | 0.0 | 0.0 | 0.0 | 0.0 | 0.1 | 0.0 | 0.0 | 0.0 | 0.0 | 0.1 | 0.0 | 0.0 | 0.0 | 0.0 | 0.0 | 0.0 | 0.1 | 0.0 | 0.0 | 0.0 | 0.0 | 0.0 | 0.0 |
| *Psychrobacter* | 0.0 | 0.0 | 0.0 | 0.4 | 0.0 | 0.0 | 0.0 | 0.0 | 0.0 | 0.0 | 0.0 | 0.0 | 0.0 | 0.0 | 0.0 | 0.0 | 0.0 | 0.1 | 0.1 | 0.0 | 0.0 | 0.0 | 0.2 | 0.2 | 0.0 |
| *Pantoea* | 0.0 | 0.0 | 0.0 | 0.0 | 0.0 | 0.0 | 0.0 | 0.0 | 0.0 | 0.0 | 0.0 | 0.0 | 0.0 | 0.0 | 0.0 | 0.0 | 0.0 | 0.0 | 0.0 | 0.0 | 0.0 | 0.0 | 0.0 | 0.0 | 0.0 |
| *Cupriavidus* | 0.0 | 0.0 | 0.2 | 0.1 | 0.3 | 0.0 | 0.0 | 0.1 | 0.2 | 0.3 | 0.0 | 0.0 | 0.1 | 0.2 | 0.1 | 0.0 | 0.1 | 0.0 | 0.0 | 0.0 | 0.0 | 0.0 | 0.0 | 0.2 | 0.1 |
| *Simonsiella* | 0.0 | 0.0 | 0.0 | 0.0 | 0.0 | 0.0 | 0.0 | 0.0 | 0.0 | 0.0 | 0.0 | 0.0 | 0.1 | 0.4 | 1.1 | 0.0 | 0.0 | 0.0 | 0.1 | 0.0 | 0.0 | 0.0 | 0.0 | 0.0 | 0.1 |
| *Lautropia* | 0.0 | 0.0 | 0.0 | 0.0 | 0.0 | 0.0 | 0.0 | 0.0 | 0.0 | 0.0 | 0.0 | 0.0 | 0.0 | 0.0 | 0.0 | 0.0 | 0.0 | 0.0 | 0.0 | 0.0 | 0.0 | 0.0 | 0.0 | 0.0 | 0.0 |
| *Erwinia* | 0.0 | 0.0 | 0.0 | 0.0 | 0.0 | 0.0 | 0.0 | 0.0 | 0.0 | 0.0 | 0.0 | 0.0 | 0.0 | 0.0 | 0.0 | 0.0 | 0.0 | 0.0 | 0.0 | 0.0 | 0.0 | 0.0 | 0.0 | 0.0 | 0.0 |
| *Nigerium* | 0.0 | 0.1 | 0.1 | 0.1 | 0.1 | 0.0 | 0.1 | 0.1 | 0.1 | 0.2 | 0.0 | 0.0 | 0.1 | 0.1 | 0.0 | 0.0 | 0.1 | 0.1 | 0.1 | 0.0 | 0.0 | 0.0 | 0.0 | 0.1 | 0.1 |
| *Aliterella* | 0.0 | 0.0 | 0.0 | 0.0 | 0.0 | 0.0 | 0.0 | 0.0 | 0.0 | 0.0 | 0.0 | 0.0 | 0.0 | 0.0 | 0.0 | 0.0 | 0.1 | 0.2 | 0.0 | 0.0 | 0.0 | 0.0 | 0.0 | 0.0 | 0.0 |
| *Aggregatibacter* | 0.0 | 0.0 | 0.0 | 0.0 | 0.0 | 0.0 | 0.3 | 0.0 | 0.0 | 0.0 | 0.0 | 0.0 | 0.0 | 0.0 | 0.0 | 0.0 | 0.0 | 0.0 | 0.0 | 0.0 | 0.0 | 0.0 | 0.2 | 0.0 | 0.0 |
| *Dietzia* | 0.0 | 0.2 | 0.1 | 0.1 | 0.2 | 0.0 | 0.1 | 0.1 | 0.0 | 0.0 | 0.0 | 0.1 | 0.1 | 0.0 | 0.0 | 0.0 | 0.0 | 0.0 | 0.0 | 0.0 | 0.0 | 0.0 | 0.0 | 0.0 | 0.0 |
| *Bifidobacterium* | 0.0 | 0.1 | 0.0 | 0.0 | 0.0 | 0.0 | 0.0 | 0.0 | 0.1 | 0.0 | 0.0 | 0.0 | 0.0 | 0.0 | 0.0 | 0.0 | 0.0 | 0.0 | 0.0 | 0.0 | 0.0 | 0.0 | 0.0 | 0.0 | 0.0 |
| *Vitreoscilla* | 0.0 | 0.0 | 0.0 | 0.0 | 0.0 | 0.0 | 0.0 | 0.0 | 0.0 | 0.0 | 0.0 | 0.0 | 0.1 | 0.5 | 2.2 | 0.0 | 0.0 | 0.0 | 0.1 | 0.0 | 0.0 | 0.0 | 0.0 | 0.0 | 0.1 |
| *Tsukamurella* | 0.0 | 0.1 | 0.1 | 0.1 | 0.1 | 0.0 | 0.0 | 0.1 | 0.0 | 0.0 | 0.0 | 0.0 | 0.0 | 0.0 | 0.0 | 0.0 | 0.0 | 0.0 | 0.0 | 0.0 | 0.0 | 0.0 | 0.0 | 0.0 | 0.0 |
| *Schaalia* | 0.0 | 0.0 | 0.0 | 0.0 | 0.0 | 0.0 | 0.2 | 0.0 | 0.0 | 0.0 | 0.0 | 0.1 | 0.0 | 0.0 | 0.0 | 0.0 | 0.0 | 0.0 | 0.0 | 0.3 | 0.0 | 0.0 | 0.0 | 0.0 | 0.0 |
| *Gloeocapsopsis* | 0.0 | 0.0 | 0.0 | 0.2 | 0.0 | 0.0 | 0.0 | 0.0 | 0.1 | 0.0 | 0.0 | 0.0 | 0.0 | 0.0 | 0.0 | 0.0 | 0.0 | 0.1 | 0.0 | 0.0 | 0.0 | 0.0 | 0.0 | 0.0 | 0.0 |
| *Klebsiella* | 0.0 | 0.1 | 0.4 | 0.0 | 0.1 | 0.0 | 0.0 | 0.0 | 0.0 | 0.0 | 0.0 | 0.9 | 0.0 | 0.0 | 0.0 | 0.0 | 0.0 | 0.0 | 0.0 | 0.0 | 0.0 | 0.0 | 0.0 | 0.0 | 0.0 |
| *Rhizobium* | 0.0 | 0.0 | 0.0 | 0.0 | 0.0 | 0.0 | 0.2 | 0.0 | 0.0 | 0.0 | 0.0 | 0.0 | 0.0 | 0.0 | 0.0 | 0.0 | 0.0 | 0.0 | 0.0 | 0.0 | 0.0 | 0.0 | 0.0 | 0.1 | 0.0 |
| *Nocardia* | 0.0 | 0.1 | 0.0 | 0.0 | 0.0 | 0.0 | 0.0 | 0.1 | 0.0 | 0.0 | 0.0 | 0.0 | 0.0 | 0.1 | 0.0 | 0.0 | 0.0 | 0.0 | 0.0 | 0.0 | 0.0 | 0.0 | 0.0 | 0.0 | 0.0 |
| *Brachybacterium* | 0.0 | 0.0 | 0.1 | 0.0 | 0.0 | 0.0 | 0.0 | 0.0 | 0.0 | 0.0 | 0.0 | 0.0 | 0.0 | 0.0 | 0.0 | 0.0 | 0.0 | 0.0 | 0.0 | 0.0 | 0.0 | 0.0 | 0.0 | 0.0 | 0.0 |
| *Janibacter* | 0.0 | 0.0 | 0.0 | 0.0 | 0.0 | 0.0 | 0.0 | 0.0 | 0.0 | 0.0 | 0.0 | 0.0 | 0.0 | 0.0 | 0.0 | 0.0 | 0.0 | 0.0 | 0.0 | 0.0 | 0.0 | 0.0 | 0.0 | 0.0 | 0.0 |
| *Lysobacter* | 0.0 | 0.0 | 0.0 | 0.0 | 0.0 | 0.0 | 0.0 | 0.0 | 0.0 | 0.0 | 0.0 | 0.0 | 0.0 | 0.0 | 0.0 | 0.0 | 0.0 | 0.0 | 0.0 | 0.0 | 0.0 | 0.0 | 0.0 | 0.0 | 0.0 |
| *Facklamia* | 0.0 | 0.0 | 0.0 | 0.0 | 0.0 | 0.0 | 0.0 | 0.0 | 0.0 | 0.0 | 0.0 | 0.0 | 0.0 | 0.0 | 0.0 | 0.0 | 0.0 | 0.0 | 0.0 | 0.0 | 0.0 | 0.0 | 0.0 | 0.0 | 0.0 |
| *Sphingopyxis* | 0.0 | 0.0 | 0.0 | 0.0 | 0.0 | 0.0 | 0.0 | 0.0 | 0.0 | 0.0 | 0.0 | 0.0 | 0.0 | 0.0 | 0.0 | 0.0 | 0.0 | 0.0 | 0.0 | 0.0 | 0.0 | 0.0 | 0.0 | 0.0 | 0.0 |
| *Flavobacterium* | 0.0 | 0.0 | 0.0 | 0.0 | 0.0 | 0.0 | 0.0 | 0.0 | 0.0 | 0.0 | 0.0 | 0.0 | 0.0 | 0.0 | 0.0 | 0.0 | 0.0 | 0.0 | 0.0 | 0.0 | 0.0 | 0.0 | 0.0 | 0.0 | 0.0 |
| *Stenotrophomonas* | 0.0 | 0.0 | 0.0 | 0.0 | 0.0 | 0.0 | 0.0 | 0.0 | 0.0 | 0.0 | 0.0 | 0.0 | 0.0 | 0.0 | 0.0 | 0.0 | 0.0 | 0.0 | 0.2 | 0.0 | 0.0 | 0.0 | 0.0 | 0.0 | 0.0 |
| *Afipia* | 0.0 | 0.0 | 0.0 | 0.1 | 0.0 | 0.0 | 0.0 | 0.0 | 0.1 | 0.0 | 0.0 | 0.0 | 0.2 | 0.0 | 0.2 | 0.0 | 0.0 | 0.0 | 0.0 | 0.0 | 0.0 | 0.0 | 0.0 | 0.2 | 0.1 |
| *Pseudoglutamicibacter* | 0.0 | 0.0 | 0.0 | 0.0 | 0.1 | 0.0 | 0.0 | 0.0 | 0.0 | 0.0 | 0.0 | 0.0 | 0.0 | 0.0 | 0.0 | 0.0 | 0.0 | 0.0 | 0.0 | 0.0 | 0.0 | 0.0 | 0.0 | 0.0 | 0.0 |
| *Hydrobacter* | 0.0 | 0.0 | 0.0 | 0.0 | 0.0 | 0.0 | 0.0 | 0.4 | 0.0 | 0.0 | 0.0 | 0.0 | 0.0 | 0.1 | 0.0 | 0.0 | 0.0 | 0.0 | 0.0 | 0.0 | 0.0 | 0.0 | 0.0 | 0.0 | 0.1 |
| *Dermabacter* | 0.0 | 0.1 | 1.7 | 0.0 | 0.3 | 0.0 | 0.1 | 0.0 | 0.0 | 0.0 | 0.0 | 0.0 | 0.0 | 0.0 | 0.0 | 0.0 | 0.0 | 0.0 | 0.0 | 0.0 | 0.0 | 0.1 | 0.1 | 0.0 | 0.0 |
| *Pectobacterium* | 0.0 | 0.0 | 0.0 | 0.0 | 0.0 | 0.0 | 0.0 | 0.0 | 0.0 | 0.0 | 0.0 | 0.1 | 0.0 | 0.0 | 0.0 | 0.0 | 0.0 | 0.0 | 0.0 | 0.0 | 0.0 | 0.0 | 0.0 | 0.0 | 0.0 |
| *Stanieria* | 0.0 | 0.0 | 0.0 | 0.0 | 0.0 | 0.0 | 0.0 | 0.0 | 0.0 | 0.0 | 0.0 | 0.0 | 0.0 | 0.0 | 0.0 | 0.0 | 0.0 | 0.0 | 0.0 | 0.0 | 0.0 | 0.0 | 0.0 | 0.0 | 0.0 |
| *Capnocytophaga* | 0.0 | 0.0 | 0.0 | 0.0 | 0.0 | 0.0 | 0.1 | 0.0 | 0.0 | 0.0 | 0.0 | 0.0 | 0.0 | 0.0 | 0.0 | 0.0 | 0.0 | 0.0 | 0.2 | 0.0 | 0.0 | 0.0 | 0.0 | 0.0 | 0.0 |
| Other genera | 3.4 | 3.7 | 3.5 | 9.6 | 4.8 | 4.1 | 2.8 | 1.7 | 3.7 | 2.8 | 1.6 | 3.2 | 4.0 | 2.6 | 4.2 | 2.2 | 3.3 | 2.0 | 3.8 | 3.0 | 11.4 | 3.1 | 4.1 | 16.1 | 2.9 |

| | Subject_23 | | | | | Subject_24 | | | | | Subject_25 | | | | | Subject_26 | | | | | Subject_27 | | | | |
|---|---|---|---|---|---|---|---|---|---|---|---|---|---|---|---|---|---|---|---|---|---|---|---|---|---|
| Taxa | Baseline | Week 2 | Week 4 | Week 8 | Week 12 | Baseline | Week 2 | Week 4 | Week 8 | Week 12 | Baseline | Week 2 | Week 4 | Week 8 | Week 12 | Baseline | Week 2 | Week 4 | Week 8 | Week 12 | Baseline | Week 2 | Week 4 | Week 8 | Week 12 |
| *Cutibacterium* | 16.0 | 4.9 | 12.4 | 19.2 | 62.8 | 27.8 | 29.9 | 27.0 | 49.3 | 47.6 | 17.3 | 29.8 | 32.6 | 36.2 | 32.2 | 3.5 | 2.1 | 2.9 | 8.7 | 8.8 | 14.2 | 13.5 | 7.9 | 11.8 | 27.7 |
| *Staphylococcus* | 43.8 | 22.2 | 22.4 | 26.7 | 7.1 | 53.0 | 30.2 | 20.8 | 20.5 | 15.0 | 41.7 | 22.8 | 23.4 | 20.5 | 12.5 | 23.7 | 8.1 | 13.0 | 9.3 | 4.6 | 41.1 | 26.6 | 21.5 | 9.9 | 4.9 |
| *Corynebacterium* | 10.6 | 16.9 | 28.2 | 13.9 | 9.6 | 2.5 | 3.5 | 12.0 | 4.0 | 2.3 | 14.8 | 12.0 | 11.8 | 8.5 | 21.1 | 4.8 | 2.2 | 4.8 | 8.8 | 0.9 | 16.7 | 21.6 | 17.3 | 1.9 | 14.0 |
| *Streptococcus* | 13.3 | 13.7 | 1.2 | 1.7 | 5.0 | 8.3 | 10.7 | 7.0 | 6.7 | 11.8 | 4.4 | 4.2 | 4.2 | 7.6 | 11.5 | 49.7 | 28.9 | 32.1 | 48.9 | 18.2 | 12.2 | 8.6 | 19.9 | 23.5 | 23.1 |
| *Snodgrassella* | 0.0 | 0.0 | 0.2 | 0.2 | 0.2 | 0.0 | 0.0 | 0.0 | 0.0 | 0.0 | 0.0 | 0.0 | 0.0 | 0.2 | 0.0 | 0.0 | 0.1 | 0.1 | 0.0 | 0.1 | 0.0 | 0.0 | 0.0 | 0.0 | 0.0 |
| *Moraxella* | 4.7 | 4.6 | 0.8 | 0.2 | 0.6 | 0.1 | 0.0 | 3.4 | 2.2 | 5.0 | 12.8 | 11.2 | 5.7 | 2.8 | 1.6 | 0.8 | 0.8 | 2.3 | 0.1 | 3.2 | 8.2 | 8.2 | 1.6 | 4.6 | 9.6 |
| *Finegoldia* | 1.1 | 0.6 | 0.3 | 0.9 | 1.2 | 0.0 | 0.0 | 0.5 | 1.5 | 0.6 | 1.3 | 1.0 | 1.5 | 9.1 | 1.4 | 0.1 | 0.1 | 0.3 | 1.9 | 0.4 | 0.0 | 0.3 | 0.2 | 0.0 | 0.0 |
| *Acinetobacter* | 0.0 | 0.6 | 0.0 | 0.0 | 0.0 | 0.8 | 2.8 | 2.4 | 0.8 | 0.3 | 0.1 | 0.1 | 0.5 | 0.0 | 0.0 | 0.0 | 0.3 | 0.2 | 0.0 | 0.0 | 0.0 | 0.2 | 0.0 | 0.0 | 0.0 |
| *Deinococcus* | 0.0 | 0.0 | 0.0 | 0.0 | 0.0 | 0.0 | 0.0 | 1.0 | 0.0 | 0.0 | 0.0 | 0.0 | 0.0 | 0.0 | 0.0 | 0.0 | 0.0 | 0.0 | 0.0 | 0.0 | 0.0 | 0.0 | 0.4 | 0.0 | 0.1 |
| *Roseomonas* | 0.0 | 0.0 | 3.1 | 1.2 | 1.6 | 0.0 | 0.1 | 0.0 | 0.0 | 0.8 | 0.0 | 0.0 | 0.0 | 0.0 | 0.0 | 0.0 | 0.0 | 0.2 | 0.0 | 0.5 | 0.0 | 0.1 | 0.1 | 0.0 | 0.0 |
| *Neisseria* | 0.0 | 0.3 | 0.1 | 0.0 | 0.0 | 0.0 | 0.0 | 0.0 | 0.0 | 0.1 | 0.0 | 0.1 | 0.0 | 0.1 | 0.1 | 0.0 | 7.9 | 2.4 | 0.1 | 3.1 | 0.0 | 1.4 | 3.1 | 2.1 | 1.5 |
| *Rothia* | 1.6 | 1.5 | 0.3 | 1.3 | 0.0 | 0.0 | 0.0 | 1.2 | 2.7 | 1.5 | 0.0 | 0.3 | 0.5 | 0.7 | 0.9 | 4.8 | 5.8 | 7.5 | 15.9 | 3.2 | 0.7 | 1.6 | 2.7 | 8.0 | 3.4 |
| *Prevotella* | 0.0 | 0.3 | 0.0 | 0.0 | 1.1 | 0.0 | 0.0 | 2.2 | 0.0 | 0.1 | 0.1 | 0.5 | 0.2 | 0.5 | 0.7 | 1.0 | 2.1 | 1.7 | 0.6 | 2.0 | 0.1 | 0.8 | 1.2 | 0.5 | 0.1 |
| *Anaerococcus* | 0.1 | 0.2 | 0.4 | 0.2 | 0.3 | 0.2 | 2.0 | 1.2 | 1.1 | 0.5 | 1.0 | 2.4 | 1.1 | 1.5 | 10.0 | 0.0 | 0.0 | 0.6 | 0.9 | 0.4 | 0.0 | 0.2 | 0.4 | 0.0 | 0.0 |
| *Kocuria* | 0.0 | 0.3 | 0.6 | 0.1 | 0.0 | 0.1 | 0.0 | 0.2 | 0.3 | 1.2 | 0.0 | 0.1 | 0.0 | 0.1 | 0.1 | 0.0 | 0.9 | 2.0 | 0.2 | 2.2 | 0.0 | 0.6 | 0.5 | 2.1 | 0.4 |
| *Haemophilus* | 0.0 | 0.0 | 0.0 | 0.0 | 0.0 | 0.0 | 0.0 | 0.0 | 0.0 | 0.4 | 0.0 | 0.0 | 0.0 | 0.4 | 0.0 | 0.0 | 2.0 | 2.6 | 0.0 | 8.3 | 0.1 | 0.9 | 3.4 | 8.9 | 1.2 |
| *Paracoccus* | 0.0 | 0.3 | 0.3 | 0.0 | 0.0 | 0.0 | 0.4 | 0.0 | 0.1 | 3.7 | 0.0 | 0.0 | 0.0 | 0.0 | 0.0 | 0.0 | 0.0 | 0.3 | 0.0 | 0.0 | 0.0 | 1.8 | 0.1 | 0.1 | 0.0 |
| *Lawsonella* | 0.0 | 0.0 | 0.0 | 0.3 | 0.0 | 0.0 | 0.0 | 0.5 | 0.9 | 0.4 | 0.0 | 0.2 | 0.2 | 1.3 | 0.4 | 0.1 | 0.0 | 0.1 | 0.7 | 0.6 | 0.0 | 0.8 | 0.5 | 0.0 | 1.6 |
| *Bacillus* | 0.1 | 0.7 | 0.3 | 0.4 | 0.1 | 0.0 | 0.7 | 0.4 | 0.8 | 0.2 | 0.0 | 0.4 | 0.4 | 0.1 | 0.2 | 0.0 | 0.4 | 0.6 | 0.0 | 0.2 | 0.0 | 0.5 | 0.8 | 0.1 | 0.1 |
| *Methylobacterium* | 0.2 | 0.6 | 0.7 | 3.1 | 0.4 | 0.0 | 0.0 | 0.0 | 0.2 | 0.0 | 0.0 | 0.1 | 0.1 | 0.0 | 0.1 | 0.0 | 0.0 | 0.0 | 0.0 | 0.2 | 0.0 | 0.0 | 0.1 | 0.0 | 0.0 |
| *Veillonella* | 0.0 | 0.0 | 0.0 | 0.0 | 0.0 | 0.0 | 0.0 | 0.0 | 0.2 | 0.1 | 0.0 | 1.5 | 0.2 | 0.9 | 0.1 | 0.2 | 2.9 | 0.8 | 0.3 | 4.0 | 0.0 | 0.4 | 0.7 | 4.0 | 1.0 |
| *Granulicatella* | 0.0 | 1.2 | 0.0 | 0.0 | 0.0 | 0.0 | 0.0 | 0.0 | 0.0 | 0.4 | 0.0 | 0.0 | 0.0 | 0.6 | 0.3 | 0.8 | 2.9 | 2.4 | 0.1 | 2.3 | 0.1 | 0.5 | 1.7 | 6.2 | 2.7 |
| *Sphingomonas* | 0.1 | 0.3 | 0.7 | 0.6 | 1.0 | 0.1 | 0.6 | 1.7 | 0.0 | 0.0 | 0.0 | 1.0 | 0.4 | 0.0 | 0.0 | 0.0 | 0.0 | 0.5 | 0.0 | 0.4 | 0.0 | 0.5 | 0.2 | 0.3 | 0.0 |
| *Williamsia* | 0.0 | 0.0 | 0.0 | 0.0 | 0.0 | 0.0 | 0.0 | 0.0 | 0.0 | 0.0 | 0.0 | 0.0 | 0.0 | 0.0 | 0.0 | 0.0 | 0.0 | 0.4 | 0.0 | 0.5 | 0.0 | 0.1 | 0.0 | 0.4 | 0.0 |
| *Peptoniphilus* | 0.0 | 0.0 | 0.0 | 0.0 | 0.7 | 0.0 | 0.0 | 0.4 | 0.3 | 0.2 | 0.2 | 0.6 | 0.8 | 2.4 | 1.1 | 0.0 | 0.0 | 0.4 | 0.6 | 0.1 | 0.0 | 0.2 | 0.1 | 0.0 | 0.0 |
| *Arachnia* | 0.0 | 0.1 | 0.0 | 0.1 | 0.7 | 0.0 | 0.1 | 0.2 | 0.2 | 0.5 | 0.0 | 0.2 | 0.1 | 0.3 | 0.1 | 0.0 | 0.1 | 0.1 | 0.1 | 0.3 | 0.0 | 0.1 | 0.0 | 0.0 | 0.2 |
| *Escherichia* | 0.0 | 0.0 | 0.0 | 0.0 | 0.0 | 0.0 | 0.0 | 0.5 | 0.0 | 0.0 | 0.0 | 0.0 | 0.0 | 0.0 | 0.0 | 0.0 | 0.0 | 0.0 | 0.0 | 0.0 | 0.0 | 0.0 | 0.0 | 0.0 | 0.0 |
| *Skermanella* | 0.0 | 0.0 | 0.3 | 1.1 | 0.0 | 0.0 | 0.0 | 0.0 | 0.0 | 0.1 | 0.0 | 0.0 | 0.1 | 0.1 | 0.0 | 0.0 | 0.0 | 0.0 | 0.0 | 0.0 | 0.0 | 0.0 | 0.0 | 0.0 | 0.0 |
| *Microcoleus* | 0.1 | 7.2 | 1.0 | 0.6 | 0.6 | 0.0 | 0.3 | 0.8 | 0.5 | 0.0 | 0.0 | 0.3 | 0.0 | 0.0 | 0.0 | 0.0 | 0.0 | 0.0 | 0.0 | 0.0 | 0.0 | 0.0 | 0.0 | 0.0 | 0.0 |
| *Serratia* | 0.0 | 0.1 | 0.0 | 1.3 | 0.0 | 0.0 | 0.0 | 0.0 | 0.4 | 0.4 | 0.0 | 0.0 | 0.0 | 0.1 | 0.1 | 0.0 | 0.0 | 0.0 | 0.8 | 0.0 | 0.0 | 0.2 | 0.7 | 0.0 | 0.0 |
| *Pseudomonas* | 0.0 | 1.7 | 1.0 | 3.4 | 2.0 | 0.0 | 0.1 | 0.0 | 0.0 | 0.0 | 0.0 | 0.3 | 0.0 | 0.0 | 0.1 | 0.0 | 1.2 | 0.0 | 0.0 | 0.1 | 0.0 | 0.0 | 0.0 | 0.0 | 0.0 |
| *Gemella* | 0.0 | 0.0 | 0.0 | 0.0 | 0.0 | 0.0 | 0.0 | 0.0 | 0.1 | 0.1 | 0.0 | 0.0 | 0.0 | 0.3 | 0.2 | 1.1 | 2.1 | 1.5 | 0.1 | 2.9 | 0.4 | 1.1 | 2.6 | 6.0 | 1.4 |
| *Porphyromonas* | 0.0 | 0.0 | 0.0 | 0.0 | 0.0 | 0.0 | 0.0 | 0.0 | 0.0 | 0.0 | 0.0 | 0.0 | 0.3 | 0.0 | 0.1 | 0.6 | 5.3 | 1.9 | 0.0 | 4.0 | 0.0 | 0.1 | 0.2 | 0.0 | 0.0 |
| *Abiotrophia* | 0.0 | 0.0 | 0.0 | 0.0 | 0.0 | 0.0 | 0.0 | 0.0 | 0.0 | 0.0 | 0.0 | 0.0 | 0.0 | 0.0 | 0.0 | 0.0 | 4.8 | 0.6 | 0.0 | 12.9 | 0.0 | 0.1 | 0.3 | 0.0 | 0.1 |
| *Mycolicibacterium* | 0.0 | 0.0 | 1.8 | 0.0 | 0.1 | 0.0 | 0.0 | 0.1 | 0.1 | 0.0 | 0.0 | 0.1 | 0.0 | 0.1 | 0.1 | 0.0 | 0.0 | 0.0 | 0.1 | 0.2 | 0.0 | 0.1 | 0.2 | 0.0 | 0.1 |
| *Enterococcus* | 0.0 | 0.4 | 0.0 | 0.0 | 0.0 | 0.0 | 0.2 | 0.2 | 0.4 | 0.1 | 0.0 | 0.2 | 0.1 | 0.1 | 0.1 | 0.0 | 1.9 | 1.2 | 0.0 | 0.8 | 0.0 | 0.3 | 0.9 | 2.5 | 0.6 |
| *Actinomyces* | 0.0 | 0.0 | 0.0 | 0.0 | 0.3 | 0.0 | 0.0 | 0.0 | 0.3 | 0.2 | 0.0 | 0.1 | 0.1 | 0.1 | 0.2 | 0.2 | 2.4 | 0.4 | 0.1 | 0.4 | 0.0 | 0.3 | 1.2 | 0.1 | 0.1 |
| *Massilia* | 0.0 | 0.0 | 0.3 | 0.0 | 0.0 | 0.0 | 0.0 | 0.0 | 0.0 | 0.0 | 2.0 | 2.5 | 4.8 | 0.7 | 0.4 | 0.0 | 0.0 | 0.0 | 0.0 | 0.0 | 0.0 | 0.1 | 0.0 | 0.0 | 0.0 |
| *Brevundimonas* | 0.0 | 0.1 | 0.0 | 0.0 | 0.0 | 0.0 | 0.0 | 0.0 | 0.0 | 0.0 | 0.0 | 0.3 | 0.0 | 0.0 | 0.0 | 0.0 | 0.0 | 0.0 | 0.0 | 0.3 | 0.0 | 0.4 | 0.1 | 0.9 | 0.2 |
| *Micrococcus* | 0.0 | 0.4 | 0.2 | 0.6 | 0.0 | 0.0 | 0.0 | 0.6 | 0.0 | 0.2 | 0.0 | 0.1 | 0.0 | 0.0 | 0.0 | 0.0 | 0.1 | 0.6 | 0.1 | 0.0 | 0.0 | 0.2 | 0.1 | 0.1 | 0.6 |
| *Kingella* | 0.0 | 0.0 | 0.0 | 0.0 | 0.0 | 0.0 | 0.0 | 0.0 | 0.0 | 0.0 | 0.0 | 0.2 | 0.1 | 0.0 | 0.0 | 0.0 | 0.1 | 0.0 | 0.0 | 0.0 | 0.0 | 0.0 | 0.0 | 0.0 | 0.0 |
| *Dermacoccus* | 0.0 | 0.0 | 0.0 | 0.0 | 0.0 | 0.0 | 0.0 | 0.0 | 0.0 | 0.2 | 0.0 | 0.0 | 0.0 | 0.1 | 0.0 | 0.0 | 0.1 | 1.1 | 0.2 | 0.5 | 0.1 | 0.5 | 0.1 | 0.1 | 0.1 |
| *Leptotrichia* | 0.0 | 0.1 | 0.0 | 0.0 | 0.0 | 0.0 | 0.0 | 0.0 | 0.0 | 0.1 | 0.0 | 0.0 | 0.5 | 0.0 | 0.1 | 0.0 | 1.7 | 0.4 | 0.0 | 1.5 | 0.0 | 0.3 | 0.2 | 0.0 | 0.0 |
| *Fusobacterium* | 0.0 | 0.0 | 0.0 | 0.0 | 0.0 | 0.0 | 0.0 | 0.0 | 0.1 | 0.0 | 0.0 | 0.1 | 0.3 | 0.1 | 0.1 | 0.8 | 3.2 | 0.9 | 0.1 | 1.8 | 0.0 | 0.1 | 0.5 | 0.2 | 0.1 |
| *Eikenella* | 0.0 | 0.0 | 0.0 | 0.0 | 0.0 | 0.0 | 0.0 | 0.0 | 0.0 | 0.0 | 0.0 | 0.0 | 0.0 | 0.0 | 0.0 | 0.0 | 0.2 | 0.1 | 0.0 | 0.0 | 0.0 | 0.0 | 0.1 | 0.0 | 0.0 |
| *Mycobacterium* | 0.0 | 0.0 | 0.4 | 0.1 | 0.1 | 0.0 | 0.0 | 0.1 | 0.1 | 0.0 | 0.0 | 0.1 | 0.0 | 0.1 | 0.0 | 0.0 | 0.0 | 0.0 | 0.0 | 0.0 | 0.0 | 0.1 | 0.1 | 0.0 | 0.1 |
| *Gordonia* | 0.0 | 0.1 | 0.1 | 0.0 | 0.1 | 0.0 | 2.0 | 1.0 | 0.4 | 0.1 | 0.0 | 0.1 | 0.0 | 0.1 | 0.1 | 0.0 | 0.0 | 0.1 | 0.0 | 0.1 | 0.0 | 0.2 | 0.1 | 0.0 | 0.1 |
| *Tessaracoccus* | 0.0 | 0.0 | 0.0 | 0.0 | 0.4 | 0.0 | 0.1 | 0.1 | 0.1 | 0.1 | 0.0 | 0.2 | 0.3 | 0.2 | 0.1 | 0.0 | 0.0 | 0.0 | 0.0 | 0.1 | 0.0 | 0.1 | 0.1 | 0.0 | 0.1 |
| *Rhodococcus* | 0.0 | 0.0 | 0.1 | 0.1 | 0.1 | 0.0 | 0.1 | 0.9 | 0.1 | 0.1 | 0.0 | 0.1 | 0.0 | 0.1 | 0.1 | 0.0 | 0.0 | 0.1 | 0.0 | 0.0 | 0.0 | 0.1 | 0.3 | 0.0 | 0.1 |
| *Metabacillus* | 0.0 | 0.1 | 0.0 | 0.0 | 0.0 | 0.0 | 0.1 | 0.0 | 0.0 | 0.0 | 0.0 | 0.0 | 0.0 | 0.0 | 0.0 | 0.0 | 0.0 | 0.0 | 0.0 | 0.0 | 0.0 | 0.0 | 0.0 | 0.0 | 0.0 |
| *Prevotellamassilia* | 0.0 | 0.0 | 0.0 | 0.0 | 0.0 | 0.0 | 0.0 | 0.0 | 0.0 | 0.0 | 0.0 | 0.0 | 0.0 | 0.0 | 0.0 | 0.0 | 0.5 | 4.2 | 0.0 | 0.3 | 0.0 | 0.0 | 0.0 | 0.0 | 0.0 |
| *Povalibacter* | 0.0 | 0.0 | 0.0 | 0.3 | 0.0 | 0.0 | 0.7 | 0.3 | 0.2 | 0.1 | 0.0 | 0.2 | 0.6 | 0.4 | 0.1 | 0.0 | 0.0 | 0.0 | 0.1 | 0.1 | 0.0 | 0.0 | 0.2 | 0.0 | 0.0 |
| *Loriellopsis* | 0.0 | 2.8 | 0.4 | 0.2 | 0.2 | 0.0 | 0.1 | 0.0 | 0.1 | 0.0 | 0.0 | 0.1 | 0.0 | 0.0 | 0.0 | 0.0 | 0.0 | 0.0 | 0.0 | 0.0 | 0.0 | 0.0 | 0.0 | 0.0 | 0.0 |
| *Microbacterium* | 0.0 | 0.0 | 0.0 | 0.0 | 0.0 | 0.0 | 1.2 | 0.2 | 0.7 | 0.0 | 0.0 | 0.1 | 0.0 | 0.0 | 0.0 | 0.0 | 0.0 | 0.1 | 0.0 | 0.0 | 0.0 | 0.0 | 0.0 | 0.1 | 0.1 |
| *Methylorubrum* | 0.0 | 0.2 | 0.0 | 0.0 | 0.0 | 0.0 | 0.2 | 0.0 | 0.0 | 0.0 | 0.0 | 0.0 | 0.0 | 0.0 | 0.0 | 0.0 | 0.0 | 0.0 | 0.0 | 0.1 | 0.0 | 0.0 | 0.0 | 0.0 | 0.0 |
| *Chryseobacterium* | 0.0 | 0.1 | 0.0 | 0.0 | 0.0 | 0.0 | 0.3 | 0.0 | 0.0 | 0.0 | 0.0 | 0.0 | 0.0 | 0.0 | 0.0 | 0.0 | 0.1 | 0.5 | 0.0 | 0.0 | 0.0 | 0.0 | 0.1 | 0.0 | 0.0 |
| *Brevibacterium* | 0.0 | 0.0 | 0.0 | 0.0 | 0.0 | 0.0 | 0.0 | 0.0 | 0.4 | 0.6 | 0.0 | 0.0 | 0.0 | 0.0 | 0.0 | 0.0 | 0.0 | 0.1 | 0.0 | 0.0 | 0.0 | 0.0 | 0.2 | 0.1 | 0.2 |
| *Nocardioides* | 0.0 | 0.0 | 0.0 | 0.0 | 0.3 | 0.0 | 0.1 | 0.1 | 0.1 | 0.1 | 0.0 | 0.1 | 0.0 | 0.1 | 0.0 | 0.0 | 0.0 | 0.0 | 0.0 | 0.0 | 0.0 | 0.1 | 0.0 | 0.0 | 0.1 |
| *Mammaliicoccus* | 0.0 | 0.1 | 0.1 | 0.1 | 0.0 | 0.0 | 0.1 | 0.1 | 0.0 | 0.0 | 0.0 | 0.1 | 0.1 | 0.0 | 0.0 | 0.0 | 0.1 | 0.1 | 0.0 | 0.0 | 0.0 | 0.2 | 0.3 | 0.0 | 0.1 |
| *Lactobacillus* | 0.0 | 0.0 | 0.2 | 0.0 | 0.0 | 0.0 | 0.0 | 0.6 | 0.0 | 0.0 | 0.0 | 0.2 | 0.5 | 0.1 | 0.0 | 0.0 | 0.0 | 0.1 | 0.0 | 0.0 | 0.0 | 0.0 | 0.0 | 0.0 | 0.0 |
| *Steroidobacter* | 0.0 | 0.0 | 0.0 | 0.2 | 0.0 | 0.0 | 0.5 | 0.2 | 0.1 | 0.0 | 0.0 | 0.1 | 0.4 | 0.2 | 0.1 | 0.0 | 0.0 | 0.0 | 0.0 | 0.1 | 0.0 | 0.0 | 0.1 | 0.0 | 0.0 |
| *Rahnella* | 0.0 | 2.7 | 0.2 | 5.4 | 0.0 | 0.0 | 0.0 | 0.0 | 0.0 | 0.0 | 0.0 | 0.0 | 0.0 | 0.0 | 0.0 | 0.0 | 0.0 | 0.0 | 0.0 | 0.0 | 0.0 | 0.0 | 0.0 | 0.0 | 0.0 |
| *Macrococcus* | 0.0 | 0.1 | 0.1 | 0.1 | 0.0 | 0.0 | 0.1 | 0.1 | 0.0 | 0.0 | 0.0 | 0.1 | 0.1 | 0.0 | 0.0 | 0.0 | 0.1 | 0.1 | 0.0 | 0.0 | 0.0 | 0.2 | 0.2 | 0.0 | 0.0 |
| *Pseudonocardia* | 0.0 | 0.0 | 0.0 | 0.0 | 0.1 | 0.0 | 0.0 | 0.1 | 0.0 | 0.1 | 0.0 | 0.0 | 0.0 | 0.1 | 0.0 | 0.0 | 0.0 | 0.0 | 0.0 | 0.0 | 0.0 | 0.1 | 0.1 | 0.0 | 0.1 |
| *Alysiella* | 0.0 | 0.0 | 0.0 | 0.0 | 0.0 | 0.0 | 0.0 | 0.0 | 0.0 | 0.0 | 0.0 | 0.0 | 0.0 | 0.0 | 0.0 | 0.0 | 0.0 | 0.0 | 0.0 | 0.0 | 0.0 | 0.0 | 0.0 | 0.0 | 0.0 |
| *Dialister* | 0.0 | 0.0 | 0.0 | 0.0 | 0.0 | 0.0 | 0.0 | 0.0 | 0.0 | 0.0 | 0.0 | 0.0 | 0.4 | 0.4 | 0.0 | 0.0 | 0.0 | 0.1 | 0.0 | 0.0 | 0.0 | 0.0 | 0.0 | 0.0 | 0.0 |
| *Psychrobacter* | 0.0 | 0.1 | 0.0 | 0.0 | 0.0 | 0.0 | 0.0 | 0.1 | 0.0 | 0.1 | 0.0 | 0.2 | 0.1 | 0.0 | 0.0 | 0.0 | 0.0 | 0.0 | 0.0 | 0.1 | 0.0 | 0.4 | 0.1 | 0.1 | 0.2 |
| *Pantoea* | 0.0 | 0.0 | 0.0 | 0.1 | 0.0 | 0.0 | 0.0 | 0.0 | 0.0 | 0.0 | 0.0 | 0.0 | 0.0 | 0.0 | 0.0 | 0.0 | 0.0 | 0.0 | 0.0 | 0.0 | 0.0 | 0.0 | 0.0 | 0.0 | 0.0 |
| *Cupriavidus* | 0.0 | 0.1 | 0.3 | 0.4 | 0.4 | 0.0 | 0.6 | 0.9 | 0.3 | 0.0 | 0.0 | 0.1 | 0.1 | 0.0 | 0.0 | 0.0 | 0.0 | 0.0 | 0.0 | 0.0 | 0.0 | 0.0 | 0.0 | 0.0 | 0.0 |
| *Simonsiella* | 0.0 | 0.0 | 0.0 | 0.0 | 0.0 | 0.0 | 0.0 | 0.0 | 0.0 | 0.0 | 0.0 | 0.0 | 0.0 | 0.0 | 0.0 | 0.0 | 0.0 | 0.0 | 0.0 | 0.0 | 0.0 | 0.0 | 0.0 | 0.0 | 0.0 |
| *Lautropia* | 0.0 | 0.0 | 0.0 | 0.0 | 0.0 | 0.0 | 0.0 | 0.0 | 0.0 | 0.0 | 0.0 | 0.0 | 0.0 | 0.0 | 0.0 | 0.0 | 0.2 | 0.2 | 0.0 | 0.0 | 0.0 | 0.1 | 0.0 | 0.1 | 0.0 |
| *Erwinia* | 0.0 | 0.0 | 0.0 | 0.2 | 0.0 | 0.0 | 0.0 | 0.0 | 0.0 | 0.0 | 0.0 | 0.0 | 0.0 | 0.0 | 0.0 | 0.0 | 0.0 | 0.0 | 0.0 | 0.0 | 0.0 | 0.0 | 0.0 | 0.0 | 0.0 |
| *Nigerium* | 0.0 | 0.0 | 0.0 | 0.0 | 0.2 | 0.0 | 0.1 | 0.1 | 0.1 | 0.1 | 0.0 | 0.0 | 0.0 | 0.1 | 0.0 | 0.0 | 0.0 | 0.0 | 0.0 | 0.0 | 0.0 | 0.0 | 0.0 | 0.0 | 0.0 |
| *Aliterella* | 0.0 | 1.2 | 0.2 | 0.1 | 0.1 | 0.0 | 0.0 | 0.1 | 0.1 | 0.0 | 0.0 | 0.0 | 0.0 | 0.0 | 0.0 | 0.0 | 0.0 | 0.0 | 0.0 | 0.0 | 0.0 | 0.0 | 0.0 | 0.0 | 0.1 |
| *Aggregatibacter* | 0.0 | 0.0 | 0.0 | 0.0 | 0.0 | 0.0 | 0.0 | 0.0 | 0.0 | 0.0 | 0.0 | 0.0 | 0.0 | 0.0 | 0.1 | 0.0 | 0.3 | 0.2 | 0.0 | 2.1 | 0.0 | 0.1 | 0.2 | 0.0 | 0.0 |
| *Dietzia* | 0.0 | 0.0 | 0.1 | 0.0 | 0.0 | 0.0 | 0.0 | 0.1 | 0.1 | 0.0 | 0.0 | 0.1 | 0.0 | 0.1 | 0.1 | 0.0 | 0.0 | 0.0 | 0.0 | 0.0 | 0.0 | 0.1 | 0.1 | 0.0 | 0.1 |
| *Bifidobacterium* | 0.0 | 0.0 | 0.0 | 0.0 | 0.0 | 0.0 | 0.0 | 0.0 | 0.0 | 0.0 | 0.0 | 0.0 | 0.3 | 0.0 | 0.2 | 0.0 | 0.0 | 0.1 | 0.0 | 0.0 | 0.0 | 0.0 | 0.0 | 0.0 | 0.0 |
| *Vitreoscilla* | 0.0 | 0.0 | 0.0 | 0.0 | 0.0 | 0.0 | 0.0 | 0.0 | 0.0 | 0.0 | 0.0 | 0.0 | 0.0 | 0.0 | 0.0 | 0.0 | 0.0 | 0.0 | 0.0 | 0.0 | 0.0 | 0.0 | 0.0 | 0.0 | 0.0 |
| *Tsukamurella* | 0.0 | 0.0 | 0.0 | 0.0 | 0.0 | 0.0 | 0.0 | 0.0 | 0.1 | 0.0 | 0.0 | 0.1 | 0.0 | 0.1 | 0.0 | 0.0 | 0.0 | 0.0 | 0.0 | 0.0 | 0.0 | 0.2 | 0.1 | 0.0 | 0.1 |
| *Schaalia* | 0.0 | 0.0 | 0.0 | 0.0 | 0.0 | 0.0 | 0.0 | 0.0 | 0.1 | 0.0 | 0.0 | 0.0 | 0.0 | 0.0 | 0.1 | 0.1 | 0.6 | 0.1 | 0.2 | 0.6 | 0.0 | 0.1 | 0.2 | 0.1 | 0.1 |
| *Gloeocapsopsis* | 0.0 | 0.9 | 0.2 | 0.1 | 0.1 | 0.0 | 0.1 | 0.5 | 0.1 | 0.0 | 0.0 | 0.0 | 0.2 | 0.0 | 0.0 | 0.0 | 0.0 | 0.0 | 0.0 | 0.0 | 0.0 | 0.0 | 0.0 | 0.0 | 0.0 |
| *Klebsiella* | 0.0 | 0.0 | 0.0 | 2.7 | 0.0 | 0.0 | 0.0 | 0.0 | 0.0 | 0.0 | 0.0 | 0.0 | 0.0 | 0.0 | 0.0 | 0.0 | 0.0 | 0.0 | 0.0 | 0.0 | 0.0 | 0.0 | 0.0 | 0.0 | 0.0 |
| *Rhizobium* | 0.0 | 0.0 | 0.3 | 0.0 | 0.0 | 0.0 | 0.0 | 0.0 | 0.2 | 0.0 | 0.0 | 0.0 | 0.0 | 0.0 | 0.0 | 0.0 | 0.0 | 0.0 | 0.0 | 0.0 | 0.0 | 0.0 | 0.0 | 0.0 | 0.0 |
| *Nocardia* | 0.0 | 0.0 | 0.1 | 0.0 | 0.0 | 0.0 | 0.0 | 0.1 | 0.1 | 0.0 | 0.0 | 0.0 | 0.0 | 0.0 | 0.0 | 0.0 | 0.0 | 0.0 | 0.0 | 0.0 | 0.0 | 0.1 | 0.0 | 0.0 | 0.0 |
| *Brachybacterium* | 0.0 | 0.0 | 0.0 | 0.0 | 0.0 | 0.0 | 0.0 | 2.3 | 0.0 | 0.1 | 0.0 | 0.0 | 0.0 | 0.0 | 0.0 | 0.0 | 0.0 | 0.0 | 0.0 | 0.7 | 0.0 | 0.0 | 0.0 | 0.0 | 0.0 |
| *Janibacter* | 0.0 | 0.0 | 0.0 | 0.0 | 0.0 | 0.0 | 0.0 | 0.0 | 0.0 | 0.0 | 0.0 | 0.2 | 0.0 | 0.0 | 0.0 | 0.0 | 0.0 | 0.0 | 0.0 | 0.4 | 0.0 | 0.0 | 0.0 | 0.1 | 0.1 |
| *Lysobacter* | 0.0 | 0.0 | 0.0 | 0.0 | 0.0 | 0.0 | 0.0 | 0.0 | 0.0 | 0.0 | 0.0 | 0.0 | 0.0 | 0.0 | 0.0 | 0.0 | 0.0 | 0.0 | 0.0 | 0.0 | 0.0 | 0.0 | 0.0 | 0.0 | 0.1 |
| *Facklamia* | 0.0 | 0.0 | 0.0 | 0.0 | 0.0 | 0.0 | 0.0 | 0.8 | 0.0 | 0.0 | 0.0 | 0.5 | 1.4 | 0.2 | 0.0 | 0.0 | 0.2 | 0.0 | 0.0 | 0.2 | 0.0 | 0.1 | 0.0 | 0.0 | 0.0 |
| *Sphingopyxis* | 0.0 | 0.0 | 0.0 | 0.0 | 0.0 | 0.0 | 0.0 | 0.0 | 0.0 | 0.0 | 0.0 | 0.0 | 0.0 | 0.0 | 0.0 | 0.0 | 0.0 | 0.0 | 0.0 | 0.0 | 0.0 | 0.0 | 0.0 | 0.0 | 0.0 |
| *Flavobacterium* | 0.0 | 0.0 | 0.4 | 0.0 | 0.0 | 0.0 | 0.0 | 0.0 | 0.0 | 0.0 | 0.0 | 0.0 | 0.0 | 0.0 | 0.0 | 0.0 | 0.0 | 0.0 | 0.0 | 0.0 | 0.0 | 0.0 | 0.0 | 0.0 | 0.0 |
| *Stenotrophomonas* | 0.0 | 0.2 | 0.0 | 0.0 | 0.1 | 0.0 | 0.0 | 0.0 | 0.0 | 0.0 | 0.0 | 0.0 | 0.0 | 0.0 | 0.0 | 0.0 | 0.0 | 0.0 | 0.0 | 0.0 | 0.0 | 0.0 | 0.0 | 0.0 | 0.0 |
| *Afipia* | 0.0 | 0.0 | 0.5 | 0.1 | 0.0 | 0.0 | 0.4 | 0.3 | 0.0 | 0.0 | 0.0 | 0.1 | 0.1 | 0.0 | 0.0 | 0.0 | 0.0 | 0.0 | 0.0 | 0.0 | 0.0 | 0.0 | 0.1 | 0.0 | 0.0 |
| *Pseudoglutamicibacter* | 0.0 | 0.0 | 0.0 | 0.2 | 0.0 | 0.0 | 0.0 | 0.1 | 0.0 | 0.2 | 0.0 | 0.0 | 0.0 | 0.0 | 0.0 | 0.0 | 0.1 | 0.1 | 0.0 | 0.0 | 0.0 | 0.1 | 0.0 | 0.1 | 0.0 |
| *Hydrobacter* | 0.0 | 0.0 | 0.0 | 0.0 | 0.0 | 0.0 | 0.0 | 0.0 | 0.2 | 0.0 | 0.0 | 0.0 | 0.1 | 0.1 | 0.1 | 0.0 | 0.0 | 0.0 | 0.0 | 0.3 | 0.0 | 0.0 | 0.0 | 0.0 | 0.0 |
| *Dermabacter* | 0.0 | 0.0 | 0.0 | 0.0 | 0.0 | 0.0 | 0.0 | 0.0 | 0.0 | 0.0 | 0.0 | 0.1 | 0.0 | 0.0 | 0.5 | 0.0 | 0.0 | 0.0 | 0.0 | 0.0 | 0.0 | 0.0 | 0.0 | 0.0 | 0.0 |
| *Pectobacterium* | 0.0 | 0.2 | 0.0 | 0.5 | 0.0 | 0.0 | 0.0 | 0.0 | 0.0 | 0.0 | 0.0 | 0.0 | 0.0 | 0.0 | 0.0 | 0.0 | 0.0 | 0.0 | 0.0 | 0.0 | 0.0 | 0.0 | 0.0 | 0.0 | 0.0 |
| *Stanieria* | 0.0 | 0.9 | 0.1 | 0.1 | 0.1 | 0.0 | 0.0 | 0.0 | 0.1 | 0.0 | 0.0 | 0.0 | 0.0 | 0.0 | 0.0 | 0.0 | 0.0 | 0.0 | 0.0 | 0.0 | 0.0 | 0.0 | 0.0 | 0.0 | 0.0 |
| *Capnocytophaga* | 0.0 | 0.0 | 0.0 | 0.0 | 0.0 | 0.0 | 0.0 | 0.0 | 0.0 | 0.0 | 0.0 | 0.0 | 0.0 | 0.0 | 0.0 | 0.0 | 0.4 | 0.3 | 0.0 | 0.1 | 0.0 | 0.1 | 0.4 | 0.0 | 0.1 |
| Other genera | 8.1 | 10.4 | 19.2 | 11.5 | 2.0 | 7.2 | 10.9 | 6.1 | 2.6 | 3.5 | 4.3 | 4.3 | 4.6 | 2.1 | 2.4 | 7.6 | 6.4 | 5.8 | 0.6 | 4.5 | 6.0 | 4.1 | 5.2 | 4.3 | 2.6 |

| | Subject_28 | | | | | Subject_30 | | | | | Subject_31 | | | | | Subject_32 | | | | | Subject_33 | | | | |
|---|---|---|---|---|---|---|---|---|---|---|---|---|---|---|---|---|---|---|---|---|---|---|---|---|---|
| Taxa | Baseline | Week 2 | Week 4 | Week 8 | Week 12 | Baseline | Week 2 | Week 4 | Week 8 | Week 12 | Baseline | Week 2 | Week 4 | Week 8 | Week 12 | Baseline | Week 2 | Week 4 | Week 8 | Week 12 | Baseline | Week 2 | Week 4 | Week 8 | Week 12 |
| *Cutibacterium* | 20.6 | 18.9 | 42.7 | 41.5 | 60.8 | 17.7 | 25.0 | 17.7 | 47.7 | 59.1 | 14.3 | 13.4 | 16.7 | 35.6 | 28.4 | 26.0 | 28.0 | 25.9 | 46.8 | 60.0 | 18.2 | 18.3 | 18.0 | 30.5 | 27.1 |
| *Staphylococcus* | 44.7 | 37.1 | 14.0 | 13.4 | 17.9 | 60.9 | 36.5 | 21.2 | 13.1 | 13.8 | 34.7 | 25.5 | 16.6 | 12.8 | 13.8 | 44.9 | 35.5 | 30.4 | 18.4 | 15.3 | 60.4 | 31.8 | 31.8 | 35.6 | 18.9 |
| *Corynebacterium* | 20.8 | 23.6 | 23.6 | 20.6 | 5.5 | 4.1 | 5.2 | 13.2 | 8.9 | 4.2 | 12.6 | 10.4 | 24.6 | 18.1 | 19.0 | 1.5 | 1.2 | 8.3 | 1.7 | 3.2 | 4.1 | 3.7 | 7.9 | 3.8 | 4.0 |
| *Streptococcus* | 8.3 | 6.5 | 7.8 | 10.1 | 7.2 | 10.5 | 10.4 | 18.0 | 13.1 | 4.3 | 30.5 | 24.3 | 14.6 | 10.9 | 19.8 | 13.9 | 12.3 | 15.3 | 12.7 | 6.8 | 13.1 | 11.0 | 4.5 | 5.3 | 13.2 |
| *Snodgrassella* | 0.1 | 0.4 | 1.5 | 0.4 | 0.1 | 1.6 | 6.3 | 0.2 | 4.9 | 0.2 | 0.2 | 0.1 | 0.1 | 0.3 | 0.0 | 0.0 | 0.0 | 0.0 | 0.1 | 0.1 | 0.0 | 0.0 | 0.0 | 6.7 | 0.1 |
| *Moraxella* | 0.1 | 0.3 | 0.0 | 0.2 | 0.1 | 0.6 | 1.4 | 2.6 | 0.1 | 0.5 | 0.2 | 0.3 | 1.1 | 2.6 | 0.3 | 8.8 | 12.3 | 5.2 | 4.5 | 4.9 | 0.1 | 0.1 | 0.5 | 0.1 | 0.0 |
| *Finegoldia* | 0.1 | 1.0 | 0.0 | 0.3 | 0.1 | 0.2 | 0.0 | 0.3 | 0.0 | 2.3 | 0.1 | 0.2 | 1.3 | 0.1 | 0.1 | 0.0 | 0.0 | 0.0 | 0.0 | 0.0 | 0.1 | 0.2 | 1.7 | 0.3 | 2.0 |
| *Acinetobacter* | 0.2 | 0.3 | 0.0 | 0.1 | 0.0 | 0.0 | 0.1 | 0.0 | 0.0 | 0.0 | 0.0 | 0.0 | 0.1 | 0.0 | 0.0 | 0.0 | 0.1 | 0.5 | 0.0 | 0.0 | 0.0 | 0.0 | 0.0 | 0.0 | 0.1 |
| *Deinococcus* | 0.0 | 0.1 | 0.0 | 0.2 | 0.0 | 0.0 | 0.0 | 0.2 | 0.3 | 0.7 | 0.0 | 0.0 | 0.1 | 0.2 | 0.0 | 0.0 | 0.3 | 0.0 | 0.1 | 0.1 | 0.0 | 0.0 | 0.3 | 0.0 | 0.1 |
| *Roseomonas* | 0.0 | 0.0 | 0.0 | 0.0 | 0.0 | 0.0 | 0.0 | 0.0 | 0.0 | 0.1 | 0.0 | 0.0 | 0.0 | 0.0 | 0.0 | 0.0 | 0.0 | 0.0 | 0.3 | 0.0 | 0.0 | 0.0 | 0.0 | 0.0 | 0.0 |
| *Neisseria* | 0.0 | 0.3 | 0.2 | 0.4 | 0.1 | 0.0 | 2.6 | 0.5 | 2.7 | 0.1 | 0.0 | 2.8 | 1.6 | 0.3 | 1.3 | 0.0 | 2.0 | 2.7 | 0.4 | 0.1 | 0.0 | 0.6 | 0.0 | 2.7 | 0.6 |
| *Rothia* | 0.1 | 1.2 | 1.9 | 4.2 | 1.2 | 0.0 | 0.6 | 1.1 | 0.0 | 0.0 | 3.4 | 3.2 | 1.5 | 2.0 | 1.4 | 0.0 | 0.3 | 1.5 | 0.7 | 1.3 | 0.0 | 0.2 | 0.0 | 0.0 | 1.3 |
| *Prevotella* | 0.2 | 0.2 | 0.6 | 0.0 | 0.0 | 0.0 | 0.0 | 0.0 | 0.0 | 0.5 | 0.1 | 1.2 | 1.4 | 0.6 | 1.0 | 0.0 | 0.6 | 0.0 | 0.1 | 0.0 | 0.0 | 2.4 | 0.9 | 0.0 | 2.6 |
| *Anaerococcus* | 0.1 | 0.5 | 0.0 | 0.3 | 0.5 | 0.0 | 0.0 | 0.3 | 0.1 | 4.0 | 0.1 | 1.3 | 1.6 | 1.8 | 0.5 | 0.0 | 0.2 | 0.3 | 0.0 | 0.1 | 0.1 | 2.4 | 1.1 | 0.0 | 0.4 |
| *Kocuria* | 0.0 | 0.4 | 0.4 | 0.3 | 0.2 | 0.0 | 0.1 | 1.5 | 0.1 | 0.2 | 0.0 | 0.7 | 0.2 | 0.3 | 0.1 | 0.0 | 0.0 | 0.3 | 0.1 | 0.2 | 0.0 | 0.0 | 0.3 | 0.0 | 0.2 |
| *Haemophilus* | 0.0 | 0.1 | 0.0 | 0.3 | 0.3 | 0.0 | 0.3 | 0.9 | 0.0 | 0.2 | 0.0 | 0.7 | 0.4 | 0.3 | 1.3 | 0.0 | 0.2 | 0.2 | 0.0 | 1.0 | 0.0 | 3.1 | 0.5 | 0.0 | 3.6 |
| *Paracoccus* | 0.0 | 0.0 | 0.0 | 0.0 | 0.0 | 0.0 | 0.3 | 4.8 | 0.1 | 0.2 | 0.0 | 0.2 | 0.3 | 0.0 | 0.0 | 0.0 | 0.0 | 0.2 | 0.0 | 0.0 | 0.0 | 0.0 | 0.0 | 0.0 | 0.0 |
| *Lawsonella* | 0.1 | 0.2 | 0.7 | 0.1 | 0.5 | 0.0 | 0.0 | 0.1 | 0.1 | 1.6 | 0.0 | 0.0 | 0.1 | 0.3 | 0.2 | 0.2 | 0.5 | 0.5 | 1.5 | 0.8 | 0.0 | 0.1 | 0.1 | 1.0 | 0.2 |
| *Bacillus* | 0.0 | 0.7 | 0.2 | 0.2 | 0.1 | 0.0 | 0.7 | 0.7 | 0.0 | 0.2 | 0.0 | 0.3 | 0.3 | 0.3 | 0.2 | 0.0 | 0.1 | 0.4 | 0.0 | 0.0 | 0.0 | 0.6 | 0.8 | 0.8 | 0.3 |
| *Methylobacterium* | 0.0 | 0.0 | 0.0 | 0.0 | 0.1 | 0.1 | 0.3 | 0.9 | 0.1 | 0.3 | 0.0 | 0.0 | 0.0 | 0.0 | 0.0 | 0.0 | 0.0 | 0.0 | 0.3 | 0.0 | 0.0 | 0.0 | 0.0 | 0.0 | 0.0 |
| *Veillonella* | 0.0 | 0.0 | 0.0 | 0.0 | 0.0 | 0.0 | 0.3 | 0.7 | 0.0 | 0.1 | 0.0 | 0.7 | 0.5 | 1.1 | 1.1 | 0.0 | 0.0 | 0.0 | 0.4 | 0.3 | 0.0 | 0.4 | 1.7 | 0.0 | 1.8 |
| *Granulicatella* | 0.0 | 0.2 | 0.0 | 0.2 | 0.1 | 0.0 | 0.0 | 1.2 | 0.1 | 0.2 | 0.0 | 0.4 | 1.4 | 0.6 | 1.0 | 0.0 | 0.5 | 0.6 | 0.1 | 0.4 | 0.0 | 0.1 | 0.6 | 0.0 | 3.6 |
| *Sphingomonas* | 0.0 | 0.0 | 0.0 | 0.0 | 0.0 | 0.0 | 0.1 | 0.2 | 0.5 | 0.2 | 0.0 | 0.0 | 0.0 | 0.0 | 0.0 | 0.0 | 0.0 | 0.0 | 0.8 | 0.0 | 0.0 | 0.0 | 0.1 | 0.0 | 0.0 |
| *Williamsia* | 0.0 | 0.0 | 0.0 | 0.0 | 0.0 | 0.0 | 0.0 | 0.1 | 0.0 | 0.0 | 0.0 | 0.2 | 0.4 | 0.1 | 0.0 | 0.0 | 0.1 | 0.9 | 1.3 | 0.0 | 0.0 | 0.0 | 0.0 | 0.0 | 0.0 |
| *Peptoniphilus* | 0.0 | 0.0 | 0.2 | 0.0 | 0.1 | 0.0 | 0.1 | 0.4 | 0.0 | 0.9 | 0.1 | 0.6 | 0.6 | 0.2 | 0.1 | 0.0 | 0.1 | 0.1 | 0.0 | 0.0 | 0.0 | 0.6 | 1.3 | 0.0 | 0.4 |
| *Arachnia* | 0.0 | 0.1 | 0.2 | 0.3 | 0.5 | 0.0 | 0.2 | 0.1 | 0.2 | 0.4 | 0.0 | 0.1 | 0.4 | 0.3 | 0.6 | 0.1 | 0.2 | 0.4 | 0.6 | 0.5 | 0.0 | 0.1 | 0.1 | 0.1 | 0.2 |
| *Escherichia* | 0.0 | 0.0 | 0.0 | 0.0 | 0.0 | 0.0 | 0.0 | 0.0 | 0.0 | 0.0 | 0.0 | 0.0 | 0.0 | 0.0 | 0.0 | 0.0 | 0.0 | 0.0 | 0.0 | 0.0 | 0.0 | 0.0 | 0.0 | 0.0 | 0.0 |
| *Skermanella* | 0.0 | 0.0 | 0.0 | 0.0 | 0.0 | 0.0 | 0.0 | 0.0 | 0.0 | 0.2 | 0.0 | 0.0 | 0.0 | 0.0 | 0.0 | 0.0 | 0.0 | 0.0 | 0.0 | 0.0 | 0.0 | 0.0 | 0.0 | 0.0 | 0.0 |
| *Microcoleus* | 0.0 | 0.1 | 0.0 | 0.0 | 0.2 | 0.0 | 0.0 | 0.0 | 0.1 | 0.0 | 0.0 | 0.3 | 0.0 | 0.1 | 0.0 | 0.0 | 0.0 | 0.0 | 0.0 | 0.2 | 0.0 | 0.0 | 0.0 | 2.7 | 0.2 |
| *Serratia* | 0.0 | 0.1 | 0.3 | 0.3 | 0.1 | 0.0 | 0.0 | 0.3 | 0.4 | 0.0 | 0.0 | 0.0 | 0.6 | 0.3 | 0.0 | 0.0 | 0.0 | 0.3 | 2.2 | 0.2 | 0.0 | 0.1 | 0.7 | 0.0 | 0.0 |
| *Pseudomonas* | 0.0 | 0.0 | 0.2 | 0.0 | 0.0 | 0.0 | 0.1 | 0.2 | 0.0 | 0.7 | 0.0 | 0.1 | 0.0 | 0.0 | 0.1 | 0.0 | 0.2 | 0.1 | 0.2 | 0.0 | 0.0 | 1.4 | 2.1 | 0.0 | 0.1 |
| *Gemella* | 0.0 | 0.3 | 0.0 | 0.2 | 0.1 | 0.0 | 0.0 | 0.6 | 0.0 | 0.1 | 0.0 | 0.8 | 0.3 | 0.0 | 0.4 | 0.0 | 0.0 | 0.0 | 0.0 | 0.2 | 0.0 | 0.0 | 0.0 | 0.0 | 1.2 |
| *Porphyromonas* | 0.0 | 0.0 | 0.1 | 0.0 | 0.0 | 0.0 | 0.0 | 0.0 | 0.0 | 0.0 | 0.0 | 0.4 | 0.4 | 0.5 | 0.2 | 0.1 | 2.0 | 0.1 | 0.0 | 0.1 | 0.0 | 0.8 | 0.0 | 2.0 | 2.4 |
| *Abiotrophia* | 0.0 | 0.0 | 0.0 | 0.0 | 0.0 | 0.0 | 0.0 | 0.1 | 0.0 | 0.0 | 0.0 | 0.7 | 1.4 | 0.3 | 0.3 | 0.0 | 0.0 | 0.0 | 0.2 | 0.0 | 0.0 | 2.0 | 1.0 | 0.0 | 1.2 |
| *Mycolicibacterium* | 0.0 | 0.1 | 0.0 | 0.1 | 0.1 | 0.0 | 0.0 | 0.2 | 0.0 | 0.1 | 0.0 | 0.2 | 0.1 | 0.1 | 0.1 | 0.0 | 0.1 | 0.1 | 0.2 | 0.1 | 0.0 | 0.0 | 0.2 | 0.0 | 0.0 |
| *Enterococcus* | 0.0 | 0.3 | 0.1 | 0.1 | 0.0 | 0.0 | 0.4 | 0.5 | 0.0 | 0.1 | 0.0 | 0.5 | 0.4 | 0.2 | 0.4 | 0.0 | 0.1 | 0.4 | 0.1 | 0.1 | 0.0 | 0.4 | 0.7 | 0.0 | 1.3 |
| *Actinomyces* | 0.0 | 0.0 | 0.0 | 0.0 | 0.0 | 0.0 | 0.1 | 0.2 | 0.0 | 0.4 | 0.0 | 0.1 | 0.7 | 0.1 | 0.2 | 0.0 | 0.0 | 0.1 | 0.0 | 0.2 | 0.0 | 0.4 | 0.1 | 0.0 | 0.5 |
| *Massilia* | 0.0 | 0.0 | 0.0 | 0.1 | 0.1 | 0.0 | 0.2 | 0.4 | 0.9 | 0.0 | 0.0 | 0.0 | 0.0 | 0.0 | 0.0 | 0.0 | 0.0 | 0.0 | 0.1 | 0.0 | 0.0 | 0.0 | 0.0 | 0.0 | 0.0 |
| *Brevundimonas* | 0.0 | 0.0 | 0.0 | 0.0 | 0.0 | 0.0 | 0.0 | 0.8 | 0.4 | 0.0 | 0.0 | 0.1 | 0.0 | 0.0 | 0.0 | 0.0 | 0.1 | 0.0 | 0.0 | 0.0 | 0.0 | 0.0 | 0.0 | 0.0 | 0.0 |
| *Micrococcus* | 0.0 | 0.1 | 0.1 | 0.9 | 0.2 | 0.0 | 0.0 | 1.0 | 0.0 | 0.0 | 0.0 | 0.5 | 0.1 | 0.7 | 0.0 | 0.0 | 0.0 | 0.0 | 0.0 | 0.0 | 0.0 | 0.0 | 0.0 | 0.0 | 0.0 |
| *Kingella* | 0.0 | 0.1 | 0.0 | 0.0 | 0.1 | 0.0 | 0.4 | 0.0 | 0.5 | 0.0 | 0.0 | 0.0 | 0.0 | 0.0 | 0.0 | 0.0 | 0.0 | 0.1 | 0.0 | 0.0 | 0.0 | 0.0 | 0.0 | 0.3 | 0.0 |
| *Dermacoccus* | 0.0 | 0.0 | 0.0 | 0.5 | 0.0 | 0.0 | 0.0 | 0.0 | 0.0 | 0.0 | 0.0 | 0.6 | 0.9 | 1.5 | 0.4 | 0.0 | 0.0 | 0.0 | 0.0 | 0.0 | 0.0 | 0.0 | 0.0 | 0.0 | 0.0 |
| *Leptotrichia* | 0.0 | 0.0 | 0.0 | 0.0 | 0.0 | 0.0 | 0.0 | 0.0 | 0.0 | 0.0 | 0.1 | 0.4 | 1.2 | 0.7 | 0.3 | 0.0 | 0.0 | 0.0 | 0.0 | 0.0 | 0.0 | 1.6 | 0.0 | 0.0 | 0.9 |
| *Fusobacterium* | 0.0 | 0.0 | 0.0 | 0.0 | 0.0 | 0.0 | 0.0 | 0.0 | 0.0 | 0.0 | 0.0 | 0.5 | 0.3 | 0.1 | 0.2 | 0.0 | 0.0 | 0.0 | 0.1 | 0.0 | 0.0 | 1.1 | 0.3 | 0.0 | 0.3 |
| *Eikenella* | 0.0 | 0.0 | 0.1 | 0.0 | 0.0 | 0.0 | 0.6 | 0.0 | 0.4 | 0.0 | 0.0 | 0.0 | 0.0 | 0.0 | 0.0 | 0.0 | 0.0 | 0.0 | 0.1 | 0.0 | 0.0 | 0.0 | 0.0 | 0.3 | 0.1 |
| *Mycobacterium* | 0.1 | 0.1 | 0.0 | 0.1 | 0.1 | 0.0 | 0.0 | 0.1 | 0.0 | 0.2 | 0.0 | 0.0 | 0.1 | 0.1 | 0.0 | 0.0 | 0.0 | 0.1 | 0.1 | 0.1 | 0.0 | 0.0 | 0.0 | 0.0 | 0.0 |
| *Gordonia* | 0.0 | 0.2 | 0.0 | 0.1 | 0.1 | 0.0 | 0.0 | 0.1 | 0.0 | 0.4 | 0.0 | 0.1 | 0.1 | 0.1 | 0.1 | 0.0 | 0.1 | 0.3 | 0.4 | 0.1 | 0.0 | 0.0 | 0.1 | 0.0 | 0.0 |
| *Tessaracoccus* | 0.0 | 0.1 | 0.1 | 0.2 | 0.2 | 0.0 | 0.1 | 0.1 | 0.1 | 0.2 | 0.0 | 0.0 | 0.1 | 0.1 | 0.1 | 0.0 | 0.1 | 0.1 | 0.1 | 0.2 | 0.0 | 0.0 | 0.0 | 0.1 | 0.1 |
| *Rhodococcus* | 0.0 | 0.1 | 0.0 | 0.1 | 0.1 | 0.0 | 0.0 | 0.1 | 0.0 | 0.1 | 0.0 | 0.1 | 0.1 | 0.2 | 0.0 | 0.0 | 0.1 | 0.1 | 0.1 | 0.1 | 0.0 | 0.0 | 0.0 | 0.1 | 0.1 |
| *Metabacillus* | 0.0 | 0.1 | 0.0 | 0.0 | 0.0 | 0.0 | 0.0 | 0.1 | 0.0 | 0.0 | 0.0 | 0.0 | 0.0 | 0.0 | 0.0 | 0.0 | 0.0 | 0.0 | 0.0 | 0.0 | 0.0 | 0.0 | 0.1 | 0.1 | 0.0 |
| *Prevotellamassilia* | 0.0 | 0.0 | 0.0 | 0.0 | 0.0 | 0.0 | 0.0 | 0.0 | 0.0 | 0.0 | 0.0 | 0.0 | 0.0 | 0.0 | 0.4 | 0.0 | 0.0 | 0.0 | 0.0 | 0.0 | 0.0 | 1.1 | 0.7 | 0.0 | 1.1 |
| *Povalibacter* | 0.0 | 0.0 | 0.0 | 0.3 | 0.0 | 0.0 | 0.1 | 0.1 | 0.1 | 0.1 | 0.0 | 0.2 | 0.0 | 0.0 | 0.0 | 0.0 | 0.2 | 0.0 | 0.2 | 0.1 | 0.0 | 0.0 | 0.7 | 0.0 | 0.1 |
| *Loriellopsis* | 0.0 | 0.0 | 0.0 | 0.0 | 0.1 | 0.0 | 0.0 | 0.0 | 0.0 | 0.0 | 0.0 | 0.0 | 0.0 | 0.0 | 0.0 | 0.0 | 0.0 | 0.0 | 0.0 | 0.1 | 0.0 | 0.0 | 0.0 | 0.7 | 0.0 |
| *Microbacterium* | 0.0 | 0.0 | 0.0 | 0.0 | 0.0 | 0.0 | 0.1 | 0.2 | 0.0 | 0.2 | 0.0 | 0.0 | 0.0 | 0.0 | 0.0 | 0.0 | 0.0 | 0.0 | 0.0 | 0.0 | 0.0 | 0.0 | 0.0 | 0.0 | 0.0 |
| *Methylorubrum* | 0.0 | 0.0 | 0.0 | 0.0 | 0.0 | 0.0 | 0.0 | 0.0 | 0.0 | 0.0 | 0.0 | 0.0 | 0.0 | 0.0 | 0.0 | 0.0 | 0.0 | 0.0 | 0.0 | 0.0 | 0.0 | 0.0 | 0.0 | 0.0 | 0.0 |
| *Chryseobacterium* | 0.0 | 0.0 | 0.0 | 0.0 | 0.0 | 0.0 | 0.0 | 0.0 | 0.0 | 0.1 | 0.0 | 0.0 | 0.0 | 0.0 | 0.0 | 0.0 | 0.0 | 0.4 | 0.0 | 0.0 | 0.0 | 0.0 | 0.0 | 0.0 | 0.0 |
| *Brevibacterium* | 0.0 | 0.0 | 0.0 | 0.3 | 0.0 | 0.0 | 0.0 | 0.0 | 0.0 | 0.0 | 0.0 | 0.2 | 0.0 | 0.4 | 0.2 | 0.0 | 0.0 | 0.0 | 0.0 | 0.0 | 0.0 | 0.0 | 0.0 | 0.0 | 0.1 |
| *Nocardioides* | 0.0 | 0.1 | 0.1 | 0.1 | 0.2 | 0.0 | 0.1 | 0.1 | 0.1 | 0.2 | 0.0 | 0.0 | 0.0 | 0.1 | 0.1 | 0.0 | 0.1 | 0.1 | 0.1 | 0.1 | 0.0 | 0.0 | 0.0 | 0.1 | 0.1 |
| *Mammaliicoccus* | 0.0 | 0.3 | 0.0 | 0.1 | 0.0 | 0.0 | 0.4 | 0.2 | 0.0 | 0.1 | 0.0 | 0.1 | 0.1 | 0.0 | 0.0 | 0.0 | 0.0 | 0.1 | 0.0 | 0.0 | 0.0 | 0.2 | 0.2 | 0.1 | 0.1 |
| *Lactobacillus* | 0.0 | 0.0 | 0.0 | 0.0 | 0.0 | 0.0 | 1.7 | 1.8 | 0.0 | 0.0 | 0.0 | 0.0 | 0.0 | 0.1 | 0.0 | 0.0 | 0.0 | 0.0 | 0.0 | 0.0 | 0.0 | 1.9 | 3.2 | 0.0 | 0.7 |
| *Steroidobacter* | 0.0 | 0.0 | 0.0 | 0.2 | 0.0 | 0.0 | 0.1 | 0.1 | 0.1 | 0.0 | 0.0 | 0.2 | 0.0 | 0.0 | 0.0 | 0.0 | 0.1 | 0.0 | 0.1 | 0.1 | 0.0 | 0.0 | 0.7 | 0.0 | 0.0 |
| *Rahnella* | 0.0 | 0.0 | 0.0 | 0.1 | 0.0 | 0.0 | 0.0 | 0.0 | 0.0 | 0.0 | 0.0 | 0.0 | 0.0 | 0.0 | 0.0 | 0.0 | 0.0 | 0.0 | 0.1 | 0.0 | 0.0 | 0.0 | 0.0 | 0.0 | 0.0 |
| *Macrococcus* | 0.0 | 0.2 | 0.1 | 0.1 | 0.0 | 0.0 | 0.2 | 0.1 | 0.0 | 0.1 | 0.0 | 0.0 | 0.1 | 0.0 | 0.0 | 0.0 | 0.1 | 0.1 | 0.0 | 0.0 | 0.0 | 0.2 | 0.5 | 0.2 | 0.1 |
| *Pseudonocardia* | 0.0 | 0.1 | 0.1 | 0.1 | 0.1 | 0.0 | 0.1 | 0.0 | 0.0 | 0.1 | 0.0 | 0.0 | 0.1 | 0.1 | 0.1 | 0.0 | 0.0 | 0.1 | 0.1 | 0.1 | 0.0 | 0.1 | 0.0 | 0.1 | 0.0 |
| *Alysiella* | 0.0 | 0.0 | 0.1 | 0.0 | 0.0 | 0.0 | 0.1 | 0.0 | 0.1 | 0.0 | 0.0 | 0.0 | 0.0 | 0.0 | 0.0 | 0.0 | 0.0 | 0.0 | 0.0 | 0.0 | 0.0 | 0.0 | 0.0 | 0.1 | 0.0 |
| *Dialister* | 0.0 | 0.0 | 0.0 | 0.0 | 0.0 | 0.0 | 0.0 | 0.0 | 0.0 | 0.3 | 0.0 | 0.1 | 0.4 | 0.0 | 0.0 | 0.0 | 0.0 | 0.2 | 0.0 | 0.0 | 0.0 | 0.7 | 0.0 | 0.0 | 0.1 |
| *Psychrobacter* | 0.0 | 0.0 | 0.0 | 0.0 | 0.0 | 0.0 | 0.0 | 0.1 | 0.0 | 0.0 | 0.0 | 0.0 | 0.0 | 0.0 | 0.0 | 0.0 | 0.3 | 0.1 | 0.1 | 0.1 | 0.0 | 0.0 | 0.0 | 0.0 | 0.0 |
| *Pantoea* | 0.0 | 0.0 | 0.0 | 0.0 | 0.0 | 0.0 | 0.0 | 0.0 | 0.0 | 0.0 | 0.0 | 0.0 | 0.0 | 0.0 | 0.0 | 0.0 | 0.0 | 0.0 | 0.0 | 0.0 | 0.0 | 1.9 | 3.9 | 0.0 | 0.1 |
| *Cupriavidus* | 0.0 | 0.0 | 0.0 | 0.0 | 0.0 | 0.0 | 0.0 | 0.1 | 0.0 | 0.1 | 0.0 | 0.0 | 0.0 | 0.0 | 0.0 | 0.0 | 0.1 | 0.2 | 0.0 | 0.0 | 0.0 | 0.1 | 0.1 | 0.0 | 0.0 |
| *Simonsiella* | 0.0 | 0.0 | 0.1 | 0.0 | 0.0 | 0.0 | 0.3 | 0.0 | 0.3 | 0.0 | 0.0 | 0.0 | 0.0 | 0.0 | 0.0 | 0.0 | 0.0 | 0.0 | 0.0 | 0.0 | 0.0 | 0.0 | 0.0 | 0.2 | 0.0 |
| *Lautropia* | 0.0 | 0.1 | 0.6 | 0.0 | 0.0 | 0.0 | 0.0 | 0.1 | 0.0 | 0.1 | 0.0 | 0.5 | 0.4 | 0.0 | 0.8 | 0.0 | 0.0 | 0.0 | 0.0 | 0.0 | 0.0 | 0.0 | 0.0 | 0.0 | 0.5 |
| *Erwinia* | 0.0 | 0.0 | 0.0 | 0.0 | 0.0 | 0.0 | 0.0 | 0.0 | 0.0 | 0.0 | 0.0 | 0.0 | 0.0 | 0.0 | 0.0 | 0.0 | 0.0 | 0.0 | 0.0 | 0.0 | 0.0 | 1.8 | 3.5 | 0.0 | 0.0 |
| *Nigerium* | 0.0 | 0.0 | 0.1 | 0.1 | 0.1 | 0.0 | 0.1 | 0.0 | 0.1 | 0.1 | 0.0 | 0.0 | 0.0 | 0.1 | 0.0 | 0.0 | 0.1 | 0.1 | 0.1 | 0.1 | 0.0 | 0.0 | 0.0 | 0.1 | 0.1 |
| *Aliterella* | 0.0 | 0.0 | 0.0 | 0.0 | 0.1 | 0.0 | 0.0 | 0.0 | 0.1 | 0.0 | 0.0 | 0.0 | 0.0 | 0.0 | 0.1 | 0.0 | 0.0 | 0.0 | 0.0 | 0.0 | 0.0 | 0.0 | 0.0 | 0.5 | 0.0 |
| *Aggregatibacter* | 0.0 | 0.0 | 0.0 | 0.1 | 0.1 | 0.0 | 0.0 | 0.0 | 0.0 | 0.0 | 0.0 | 0.1 | 0.2 | 0.2 | 0.7 | 0.0 | 0.0 | 0.0 | 0.0 | 0.0 | 0.0 | 0.0 | 0.0 | 0.0 | 0.1 |
| *Dietzia* | 0.0 | 0.1 | 0.1 | 0.0 | 0.0 | 0.0 | 0.0 | 0.0 | 0.0 | 0.1 | 0.0 | 0.0 | 0.1 | 0.1 | 0.0 | 0.0 | 0.0 | 0.1 | 0.1 | 0.1 | 0.0 | 0.0 | 0.0 | 0.2 | 0.0 |
| *Bifidobacterium* | 0.0 | 0.0 | 0.0 | 0.0 | 0.1 | 0.0 | 0.0 | 0.0 | 0.0 | 0.0 | 0.0 | 0.0 | 0.0 | 0.0 | 0.0 | 0.0 | 0.0 | 0.0 | 1.1 | 0.0 | 0.0 | 0.0 | 0.0 | 0.1 | 0.0 |
| *Vitreoscilla* | 0.0 | 0.0 | 0.0 | 0.0 | 0.0 | 0.0 | 0.2 | 0.0 | 0.2 | 0.0 | 0.0 | 0.0 | 0.0 | 0.0 | 0.0 | 0.0 | 0.0 | 0.0 | 0.0 | 0.0 | 0.0 | 0.0 | 0.0 | 0.2 | 0.0 |
| *Tsukamurella* | 0.0 | 0.0 | 0.0 | 0.0 | 0.0 | 0.0 | 0.0 | 0.0 | 0.0 | 0.1 | 0.0 | 0.0 | 0.0 | 0.1 | 0.0 | 0.0 | 0.1 | 0.1 | 0.1 | 0.1 | 0.0 | 0.0 | 0.0 | 0.0 | 0.0 |
| *Schaalia* | 0.0 | 0.0 | 0.0 | 0.0 | 0.0 | 0.0 | 0.1 | 0.0 | 0.0 | 0.0 | 0.0 | 0.0 | 0.0 | 0.1 | 0.0 | 0.0 | 0.1 | 0.0 | 0.2 | 0.0 | 0.0 | 0.2 | 0.0 | 0.0 | 0.2 |
| *Gloeocapsopsis* | 0.0 | 0.0 | 0.0 | 0.0 | 0.0 | 0.0 | 0.0 | 0.0 | 0.0 | 0.0 | 0.0 | 0.1 | 0.0 | 0.0 | 0.0 | 0.0 | 0.0 | 0.0 | 0.0 | 0.1 | 0.0 | 0.0 | 0.0 | 0.5 | 0.1 |
| *Klebsiella* | 0.0 | 0.0 | 0.0 | 0.0 | 0.0 | 0.0 | 0.0 | 0.1 | 0.0 | 0.0 | 0.0 | 0.0 | 0.0 | 0.0 | 0.0 | 0.0 | 0.0 | 0.0 | 0.0 | 0.0 | 0.0 | 0.1 | 0.3 | 0.0 | 0.0 |
| *Rhizobium* | 0.0 | 0.0 | 0.0 | 0.0 | 0.0 | 0.0 | 0.0 | 0.1 | 0.0 | 0.0 | 0.0 | 0.0 | 0.0 | 0.0 | 0.0 | 0.0 | 0.0 | 0.0 | 0.0 | 0.0 | 0.0 | 0.0 | 0.0 | 0.0 | 0.0 |
| *Nocardia* | 0.0 | 0.0 | 0.0 | 0.0 | 0.0 | 0.0 | 0.0 | 0.0 | 0.0 | 0.0 | 0.0 | 0.0 | 0.0 | 0.0 | 0.0 | 0.0 | 0.0 | 0.1 | 0.1 | 0.0 | 0.0 | 0.0 | 0.0 | 0.0 | 0.0 |
| *Brachybacterium* | 0.0 | 0.1 | 0.0 | 0.0 | 0.0 | 0.0 | 0.0 | 0.1 | 0.0 | 0.0 | 0.0 | 0.2 | 0.0 | 0.0 | 0.0 | 0.0 | 0.0 | 0.0 | 0.0 | 0.0 | 0.0 | 0.0 | 0.0 | 0.0 | 0.0 |
| *Janibacter* | 0.0 | 0.0 | 0.0 | 0.0 | 0.0 | 0.0 | 0.0 | 0.0 | 0.0 | 0.0 | 0.0 | 0.1 | 0.0 | 0.0 | 0.0 | 0.0 | 0.0 | 0.0 | 0.0 | 0.0 | 0.0 | 0.0 | 0.0 | 0.0 | 0.0 |
| *Lysobacter* | 0.0 | 0.0 | 0.0 | 0.0 | 0.0 | 0.0 | 0.0 | 0.0 | 0.0 | 0.0 | 0.0 | 0.0 | 0.0 | 0.0 | 0.0 | 0.0 | 0.0 | 0.2 | 0.0 | 0.0 | 0.0 | 0.0 | 0.0 | 0.0 | 0.0 |
| *Facklamia* | 0.0 | 0.0 | 0.0 | 0.0 | 0.0 | 0.0 | 0.0 | 0.0 | 0.0 | 0.0 | 0.0 | 0.0 | 0.0 | 0.1 | 0.0 | 0.0 | 0.0 | 0.0 | 0.1 | 0.0 | 0.0 | 0.1 | 0.0 | 0.0 | 0.1 |
| *Sphingopyxis* | 0.0 | 0.0 | 0.0 | 0.0 | 0.0 | 0.0 | 0.0 | 0.0 | 0.0 | 0.0 | 0.0 | 0.0 | 0.0 | 0.0 | 0.0 | 0.0 | 0.0 | 0.0 | 0.0 | 0.0 | 0.0 | 0.0 | 0.0 | 0.0 | 0.0 |
| *Flavobacterium* | 0.0 | 0.0 | 0.0 | 0.0 | 0.0 | 0.0 | 0.0 | 0.0 | 0.0 | 0.0 | 0.0 | 0.0 | 0.0 | 0.0 | 0.0 | 0.0 | 0.0 | 0.0 | 0.0 | 0.0 | 0.0 | 0.1 | 0.3 | 0.0 | 0.0 |
| *Stenotrophomonas* | 0.0 | 0.3 | 0.0 | 0.0 | 0.0 | 0.0 | 0.0 | 0.0 | 0.0 | 0.0 | 0.0 | 0.0 | 0.0 | 0.1 | 0.0 | 0.0 | 0.0 | 0.2 | 0.0 | 0.0 | 0.0 | 0.0 | 0.0 | 0.0 | 0.0 |
| *Afipia* | 0.0 | 0.1 | 0.0 | 0.0 | 0.0 | 0.0 | 0.0 | 0.1 | 0.0 | 0.0 | 0.0 | 0.1 | 0.0 | 0.0 | 0.0 | 0.0 | 0.0 | 0.1 | 0.0 | 0.0 | 0.0 | 0.1 | 0.2 | 0.0 | 0.0 |
| *Pseudoglutamicibacter* | 0.0 | 0.0 | 0.0 | 0.1 | 0.0 | 0.0 | 0.0 | 0.1 | 0.0 | 0.0 | 0.0 | 0.3 | 0.1 | 0.1 | 0.1 | 0.0 | 0.0 | 0.0 | 0.0 | 0.0 | 0.0 | 0.0 | 0.0 | 0.0 | 0.0 |
| *Hydrobacter* | 0.0 | 0.0 | 0.3 | 0.1 | 0.0 | 0.0 | 0.0 | 0.0 | 0.3 | 0.0 | 0.0 | 0.0 | 0.2 | 0.2 | 0.2 | 0.0 | 0.0 | 0.0 | 0.2 | 0.2 | 0.0 | 0.0 | 0.0 | 0.0 | 0.2 |
| *Dermabacter* | 0.0 | 0.0 | 0.0 | 0.0 | 0.0 | 0.0 | 0.1 | 0.0 | 0.0 | 0.0 | 0.0 | 0.2 | 0.0 | 0.0 | 0.0 | 0.0 | 0.0 | 0.0 | 0.0 | 0.0 | 0.0 | 0.0 | 0.0 | 0.0 | 0.0 |
| *Pectobacterium* | 0.0 | 0.0 | 0.0 | 0.0 | 0.0 | 0.0 | 0.0 | 0.0 | 0.0 | 0.0 | 0.0 | 0.0 | 0.0 | 0.0 | 0.0 | 0.0 | 0.0 | 0.0 | 0.0 | 0.0 | 0.0 | 0.1 | 0.2 | 0.0 | 0.0 |
| *Stanieria* | 0.0 | 0.0 | 0.0 | 0.0 | 0.0 | 0.0 | 0.0 | 0.0 | 0.0 | 0.0 | 0.0 | 0.0 | 0.0 | 0.0 | 0.0 | 0.0 | 0.0 | 0.0 | 0.0 | 0.1 | 0.0 | 0.0 | 0.0 | 0.5 | 0.0 |
| *Capnocytophaga* | 0.0 | 0.1 | 0.0 | 0.0 | 0.0 | 0.0 | 0.0 | 0.0 | 0.0 | 0.0 | 0.0 | 0.1 | 0.1 | 0.1 | 0.4 | 0.0 | 0.0 | 0.0 | 0.1 | 0.0 | 0.0 | 0.3 | 0.0 | 0.0 | 0.2 |
| Other genera | 4.5 | 4.2 | 3.0 | 2.4 | 2.0 | 4.3 | 3.9 | 4.5 | 3.5 | 1.5 | 3.4 | 4.9 | 5.3 | 3.8 | 3.4 | 4.4 | 1.2 | 2.2 | 2.5 | 2.2 | 3.9 | 7.1 | 7.7 | 4.0 | 6.9 |

| | Healthy control | | | | | | | | | |
|---|---|---|---|---|---|---|---|---|---|---|
| Taxa | Subject_01 | Subject_02 | Subject_03 | Subject_04 | Subject_05 | Subject_06 | Subject_07 | Subject_08 | Subject_09 | Subject_10 |
| *Cutibacterium* | 28.8 | 42.0 | 61.6 | 10.3 | 31.0 | 49.6 | 55.9 | 48.8 | 45.9 | 35.0 |
| *Staphylococcus* | 6.4 | 10.2 | 2.9 | 6.0 | 12.0 | 15.7 | 12.9 | 11.9 | 12.9 | 10.4 |
| *Corynebacterium* | 4.2 | 26.4 | 14.1 | 3.5 | 14.8 | 11.4 | 1.8 | 7.3 | 14.2 | 16.4 |
| *Streptococcus* | 14.1 | 4.2 | 4.4 | 17.8 | 11.2 | 5.6 | 1.2 | 3.1 | 1.9 | 7.0 |
| *Snodgrassella* | 0.1 | 0.2 | 0.0 | 0.2 | 0.0 | 0.1 | 0.2 | 8.1 | 7.3 | 0.1 |
| *Moraxella* | 1.9 | 0.4 | 2.5 | 8.9 | 0.4 | 2.7 | 6.7 | 0.0 | 5.6 | 3.2 |
| *Finegoldia* | 2.1 | 0.9 | 0.2 | 1.9 | 10.3 | 1.5 | 0.2 | 0.0 | 0.4 | 1.0 |
| *Acinetobacter* | 0.6 | 0.1 | 0.1 | 2.0 | 0.0 | 0.0 | 0.1 | 4.8 | 1.3 | 0.1 |
| *Deinococcus* | 1.3 | 0.0 | 1.2 | 16.8 | 0.3 | 0.6 | 0.4 | 0.5 | 0.2 | 0.3 |
| *Roseomonas* | 2.8 | 1.7 | 0.3 | 0.1 | 0.9 | 0.0 | 0.7 | 0.0 | 0.0 | 0.3 |
| *Neisseria* | 0.4 | 0.8 | 0.0 | 1.6 | 7.6 | 0.4 | 0.0 | 0.5 | 1.1 | 0.0 |
| *Rothia* | 0.3 | 0.1 | 0.4 | 1.2 | 0.1 | 0.4 | 0.1 | 0.0 | 0.0 | 0.2 |
| *Prevotella* | 3.9 | 0.0 | 0.9 | 0.8 | 0.2 | 0.1 | 0.0 | 0.0 | 0.7 | 0.0 |
| *Anaerococcus* | 0.2 | 0.5 | 0.1 | 1.3 | 0.0 | 0.0 | 0.0 | 0.0 | 0.0 | 0.1 |
| *Kocuria* | 0.2 | 0.1 | 2.3 | 0.6 | 0.2 | 0.8 | 0.6 | 2.7 | 0.0 | 0.5 |
| *Haemophilus* | 0.3 | 0.2 | 0.1 | 0.2 | 0.1 | 2.5 | 0.2 | 0.0 | 0.5 | 2.4 |
| *Paracoccus* | 0.3 | 0.2 | 0.0 | 0.2 | 0.0 | 0.1 | 0.0 | 0.0 | 0.0 | 2.0 |
| *Lawsonella* | 1.9 | 0.1 | 0.3 | 0.3 | 0.5 | 0.5 | 0.2 | 0.0 | 0.0 | 1.8 |
| *Bacillus* | 3.8 | 0.0 | 1.1 | 2.8 | 0.2 | 0.0 | 0.0 | 0.0 | 0.0 | 0.4 |
| *Methylobacterium* | 0.0 | 0.0 | 0.0 | 3.3 | 0.1 | 0.2 | 0.2 | 0.7 | 0.2 | 1.0 |
| *Veillonella* | 0.2 | 0.1 | 0.0 | 0.0 | 0.0 | 0.0 | 0.0 | 0.0 | 0.0 | 1.7 |
| *Granulicatella* | 0.1 | 0.1 | 0.0 | 0.2 | 0.1 | 0.2 | 0.6 | 0.0 | 0.2 | 0.0 |
| *Sphingomonas* | 0.4 | 0.8 | 0.1 | 0.5 | 3.2 | 0.2 | 0.1 | 0.4 | 0.1 | 0.4 |
| *Williamsia* | 0.1 | 1.6 | 0.3 | 0.1 | 0.1 | 0.9 | 0.0 | 1.2 | 0.3 | 0.0 |
| *Peptoniphilus* | 0.2 | 0.0 | 0.4 | 0.0 | 0.1 | 0.1 | 0.3 | 0.1 | 0.4 | 0.3 |
| *Arachnia* | 0.1 | 0.8 | 0.0 | 0.1 | 0.0 | 0.0 | 0.2 | 0.0 | 0.0 | 0.0 |
| *Escherichia* | 1.0 | 0.6 | 0.1 | 0.1 | 0.7 | 0.0 | 0.2 | 0.0 | 0.0 | 1.3 |
| *Skermanella* | 1.9 | 0.1 | 0.0 | 0.0 | 0.2 | 0.2 | 4.7 | 0.3 | 0.0 | 0.0 |
| *Microcoleus* | 0.1 | 0.0 | 0.2 | 5.7 | 0.9 | 1.2 | 0.2 | 0.0 | 0.0 | 0.0 |
| *Serratia* | 0.1 | 0.0 | 0.6 | 1.3 | 0.2 | 0.1 | 1.1 | 0.0 | 0.3 | 0.2 |
| *Pseudomonas* | 1.2 | 0.1 | 0.0 | 0.7 | 0.2 | 0.0 | 0.0 | 0.0 | 0.0 | 0.4 |
| *Gemella* | 2.5 | 0.1 | 0.0 | 0.0 | 0.1 | 0.0 | 0.0 | 0.0 | 0.0 | 0.0 |
| *Porphyromonas* | 1.3 | 0.1 | 0.4 | 3.2 | 0.1 | 0.0 | 0.0 | 0.0 | 0.0 | 0.3 |
| *Abiotrophia* | 1.9 | 0.1 | 0.1 | 1.7 | 0.1 | 0.0 | 0.2 | 0.0 | 0.0 | 0.4 |
| *Mycolicibacterium* | 0.9 | 0.7 | 0.1 | 0.0 | 0.1 | 0.1 | 0.2 | 0.1 | 0.1 | 0.5 |
| *Enterococcus* | 1.3 | 0.0 | 0.1 | 0.5 | 0.1 | 0.4 | 1.7 | 0.0 | 0.1 | 0.2 |
| *Actinomyces* | 1.9 | 0.0 | 0.3 | 0.9 | 0.0 | 0.2 | 0.0 | 0.0 | 0.0 | 0.2 |
| *Massilia* | 1.7 | 0.1 | 0.0 | 0.0 | 0.4 | 0.0 | 0.0 | 1.0 | 1.0 | 0.0 |
| *Brevundimonas* | 0.7 | 0.1 | 0.2 | 0.4 | 0.1 | 0.0 | 0.0 | 0.0 | 0.6 | 0.9 |
| *Micrococcus* | 0.1 | 0.1 | 0.5 | 0.3 | 0.0 | 0.4 | 0.0 | 0.0 | 0.0 | 0.0 |
| *Kingella* | 0.1 | 0.0 | 0.0 | 0.1 | 0.1 | 0.0 | 0.0 | 1.4 | 0.5 | 0.0 |
| *Dermacoccus* | 0.1 | 1.3 | 0.3 | 0.2 | 0.0 | 0.0 | 0.0 | 0.0 | 0.0 | 0.0 |
| *Leptotrichia* | 0.9 | 0.0 | 0.2 | 0.0 | 0.1 | 0.0 | 0.0 | 0.0 | 0.0 | 0.6 |
| *Fusobacterium* | 0.3 | 0.1 | 0.1 | 0.6 | 0.1 | 0.0 | 0.3 | 0.0 | 0.1 | 0.3 |
| *Eikenella* | 0.1 | 0.0 | 0.0 | 0.0 | 0.0 | 0.0 | 0.0 | 1.3 | 0.4 | 0.0 |
| *Mycobacterium* | 0.0 | 0.1 | 0.1 | 0.0 | 0.1 | 0.0 | 0.2 | 0.1 | 0.1 | 0.2 |
| *Gordonia* | 0.0 | 0.2 | 0.2 | 0.0 | 0.0 | 0.2 | 0.1 | 0.1 | 0.1 | 0.4 |
| *Tessaracoccus* | 0.1 | 0.0 | 0.2 | 0.1 | 0.1 | 0.0 | 0.3 | 0.1 | 0.2 | 0.2 |
| *Rhodococcus* | 0.1 | 0.2 | 0.2 | 0.1 | 0.1 | 0.0 | 0.2 | 0.1 | 0.1 | 0.4 |
| *Metabacillus* | 0.0 | 0.0 | 0.1 | 0.0 | 0.0 | 0.0 | 0.0 | 0.1 | 0.0 | 0.1 |
| *Prevotellamassilia* | 1.2 | 0.0 | 0.1 | 0.0 | 0.1 | 0.0 | 0.1 | 0.1 | 0.0 | 0.0 |
| *Povalibacter* | 0.1 | 0.4 | 0.1 | 0.1 | 0.0 | 0.3 | 0.0 | 0.5 | 0.2 | 0.2 |
| *Loriellopsis* | 0.9 | 0.1 | 0.0 | 0.0 | 0.1 | 0.0 | 1.0 | 0.1 | 0.1 | 0.0 |
| *Microbacterium* | 0.1 | 0.0 | 0.1 | 0.0 | 0.0 | 0.0 | 0.0 | 0.0 | 0.0 | 0.2 |
| *Methylorubrum* | 0.1 | 0.1 | 0.0 | 0.3 | 0.0 | 0.3 | 0.0 | 0.0 | 0.0 | 0.1 |
| *Chryseobacterium* | 0.1 | 0.2 | 0.0 | 0.2 | 0.1 | 0.0 | 0.1 | 0.1 | 0.1 | 1.5 |
| *Brevibacterium* | 0.1 | 0.2 | 0.0 | 0.0 | 0.0 | 0.0 | 0.0 | 0.0 | 0.0 | 1.7 |
| *Nocardioides* | 0.1 | 0.0 | 0.2 | 0.0 | 0.0 | 0.0 | 0.1 | 0.1 | 0.1 | 0.1 |
| *Mammaliicoccus* | 0.1 | 0.0 | 0.0 | 0.0 | 0.0 | 0.1 | 0.0 | 0.1 | 0.1 | 0.1 |
| *Lactobacillus* | 0.8 | 0.1 | 0.1 | 0.4 | 0.1 | 0.0 | 0.1 | 0.0 | 0.1 | 0.1 |
| *Steroidobacter* | 0.0 | 0.3 | 0.0 | 0.1 | 0.0 | 0.2 | 0.0 | 0.4 | 0.2 | 0.2 |
| *Rahnella* | 0.1 | 0.0 | 0.0 | 0.0 | 0.0 | 0.0 | 0.0 | 0.1 | 0.1 | 0.2 |
| *Macrococcus* | 0.1 | 0.1 | 0.0 | 0.0 | 0.0 | 0.1 | 0.0 | 0.1 | 0.0 | 0.2 |
| *Pseudonocardia* | 0.0 | 0.2 | 0.1 | 0.0 | 0.0 | 0.0 | 0.1 | 0.1 | 0.1 | 0.2 |
| *Alysiella* | 0.1 | 0.1 | 0.0 | 0.0 | 0.0 | 0.1 | 0.1 | 0.3 | 0.1 | 0.2 |
| *Dialister* | 0.1 | 0.8 | 0.0 | 0.0 | 0.6 | 0.2 | 0.0 | 0.1 | 0.0 | 0.2 |
| *Psychrobacter* | 0.1 | 0.0 | 0.0 | 0.2 | 0.0 | 0.1 | 0.2 | 0.1 | 0.1 | 0.1 |
| *Pantoea* | 0.1 | 0.0 | 0.0 | 0.0 | 0.0 | 0.0 | 0.1 | 0.1 | 0.1 | 0.2 |
| *Cupriavidus* | 0.1 | 0.1 | 0.0 | 0.0 | 0.1 | 0.0 | 0.2 | 0.0 | 0.1 | 0.4 |
| *Simonsiella* | 0.1 | 0.0 | 0.0 | 0.0 | 0.1 | 0.1 | 0.0 | 0.6 | 0.3 | 0.0 |
| *Lautropia* | 0.6 | 0.0 | 0.1 | 0.0 | 0.0 | 0.1 | 0.1 | 0.1 | 0.0 | 0.2 |
| *Erwinia* | 0.0 | 0.1 | 0.1 | 0.0 | 0.0 | 0.0 | 0.1 | 0.0 | 0.0 | 0.1 |
| *Nigerium* | 0.1 | 0.0 | 0.2 | 0.0 | 0.0 | 0.0 | 0.1 | 0.0 | 0.1 | 0.1 |
| *Aliterella* | 0.0 | 0.0 | 0.0 | 0.0 | 0.0 | 0.0 | 0.7 | 0.0 | 0.0 | 0.2 |
| *Aggregatibacter* | 0.1 | 0.5 | 0.0 | 0.0 | 0.0 | 0.7 | 0.0 | 0.0 | 0.0 | 0.0 |
| *Dietzia* | 0.0 | 0.1 | 0.2 | 0.0 | 0.1 | 0.1 | 0.0 | 0.1 | 0.0 | 0.2 |
| *Bifidobacterium* | 0.1 | 0.1 | 0.1 | 0.0 | 0.1 | 0.0 | 2.8 | 0.2 | 0.1 | 0.0 |
| *Vitreoscilla* | 0.1 | 0.0 | 0.1 | 0.0 | 0.0 | 0.1 | 0.0 | 1.0 | 0.3 | 0.0 |
| *Tsukamurella* | 0.0 | 0.0 | 0.2 | 0.0 | 0.0 | 0.0 | 0.1 | 0.1 | 0.1 | 0.1 |
| *Schaalia* | 0.2 | 0.0 | 0.1 | 0.1 | 0.2 | 0.2 | 0.0 | 0.1 | 0.1 | 0.4 |
| *Gloeocapsopsis* | 0.1 | 0.0 | 0.0 | 0.0 | 0.0 | 0.0 | 0.6 | 0.0 | 0.1 | 0.0 |
| *Klebsiella* | 0.1 | 0.0 | 0.1 | 0.0 | 0.0 | 0.0 | 0.0 | 0.1 | 0.1 | 0.0 |
| *Rhizobium* | 0.0 | 0.0 | 0.0 | 0.1 | 0.1 | 0.0 | 0.2 | 0.0 | 0.0 | 0.3 |
| *Nocardia* | 0.1 | 0.1 | 0.0 | 0.0 | 0.0 | 0.0 | 0.0 | 0.0 | 0.0 | 0.2 |
| *Brachybacterium* | 0.1 | 0.0 | 0.1 | 0.0 | 0.1 | 0.0 | 0.0 | 0.1 | 0.0 | 0.1 |
| *Janibacter* | 0.1 | 0.0 | 0.0 | 0.3 | 0.0 | 0.0 | 0.0 | 0.0 | 0.0 | 0.0 |
| *Lysobacter* | 0.0 | 0.0 | 0.0 | 0.0 | 0.1 | 0.0 | 0.0 | 0.0 | 0.0 | 0.0 |
| *Facklamia* | 0.1 | 0.8 | 0.0 | 0.1 | 0.1 | 0.1 | 0.1 | 0.1 | 0.0 | 0.0 |
| *Sphingopyxis* | 0.1 | 0.1 | 0.1 | 0.0 | 0.1 | 0.1 | 0.0 | 0.1 | 0.1 | 0.0 |
| *Flavobacterium* | 0.0 | 0.0 | 0.0 | 0.0 | 0.1 | 0.1 | 0.0 | 0.1 | 0.0 | 0.0 |
| *Stenotrophomonas* | 0.0 | 0.0 | 0.1 | 0.0 | 0.1 | 0.1 | 0.0 | 0.0 | 0.0 | 0.0 |
| *Afipia* | 0.1 | 0.0 | 0.1 | 0.0 | 0.1 | 0.1 | 0.0 | 0.0 | 0.1 | 0.2 |
| *Pseudoglutamicibacter* | 0.0 | 0.0 | 0.0 | 0.1 | 0.0 | 0.0 | 0.0 | 0.0 | 0.0 | 0.0 |
| *Hydrobacter* | 0.2 | 0.0 | 0.0 | 0.5 | 0.1 | 0.3 | 0.0 | 0.0 | 0.0 | 0.1 |
| *Dermabacter* | 0.2 | 0.0 | 0.1 | 0.1 | 0.0 | 0.1 | 0.1 | 0.1 | 0.1 | 0.4 |
| *Pectobacterium* | 0.1 | 0.1 | 0.0 | 0.1 | 0.0 | 0.0 | 0.0 | 0.1 | 0.1 | 0.0 |
| *Stanieria* | 0.0 | 0.0 | 0.0 | 0.0 | 0.0 | 0.0 | 0.8 | 0.0 | 0.1 | 0.0 |
| *Capnocytophaga* | 0.2 | 0.0 | 0.0 | 0.3 | 0.1 | 0.1 | 0.1 | 0.1 | 0.0 | 0.0 |
